# Discovery of a New Chalcone-Trimethoxycinnamide Hybrid with Antimitotic Effect: Design, Synthesis, and Structure—Activity Relationship Studies

**DOI:** 10.3390/ph16060879

**Published:** 2023-06-14

**Authors:** Joana Moreira, Patrícia M. A. Silva, Matilde Barros, Lucília Saraiva, Madalena Pinto, Hassan Bousbaa, Honorina Cidade

**Affiliations:** 1 Laboratory of Organic and Pharmaceutical Chemistry, Department of Chemical Sciences, Faculty of Pharmacy, University of Porto, Rua de Jorge Viterbo Ferreira 228, 4050-313 Porto, Portugal; up201302558@edu.ff.up.pt (J.M.); madalena@ff.up.pt (M.P.); 2 Interdisciplinary Centre of Marine and Environmental Research (CIIMAR), University of Porto, Edifício do Terminal de Cruzeiros do Porto de Leixões, Avenida General Norton de Matos, S/N, 4450-208 Matosinhos, Portugal; 3 UNIPRO—Oral Pathology and Rehabilitation Research Unit, University Institute of Health Sciences (IUCS), CESPU, Rua Central de Gandra, 1317, 4585-116 Gandra, Portugal; patricia.silva@cespu.pt; 4 TOXRUN—Toxicology Research Unit, University Institute of Health Sciences, CESPU, CRL, 4585-116 Gandra, Portugal; 5 LAQV/REQUIMTE, Laboratory of Microbiology, Department of Biological Sciences, Faculty of Pharmacy, University of Porto, Rua Jorge Viterbo Ferreira, 228, 4050-313 Porto, Portugal; up201604459@edu.ff.up.pt (M.B.); lucilia.saraiva@ff.up.pt (L.S.)

**Keywords:** chalcones, diarylpentanoids, hybrids, antitumor activity, apoptosis, p53, mitosis

## Abstract

In this work, the design and synthesis of a new chalcone-trimethoxycinnamide hybrid (**7**) based on the combination of subunits of two promising antiproliferative compounds (**CM-M345** (**1**) and **BP-M345** (**2**)), previously obtained by our research group, are reported. In order to expand the structure–activity relationship (SAR) knowledge, a new series of **7**-analogues was also designed and synthetized. All the compounds were evaluated for their antitumor activity against melanoma (A375-C5), breast adenocarcinoma (MCF-7), and colorectal carcinoma (HCT116) cell lines, as well as non-tumor HPAEpiC cells. Three of the newly synthesized compounds (**6**, **7**, and **13**) exhibited potent antiproliferative activity, mainly on colorectal tumor cells (GI_50_ = 2.66–3.26 μM), showing hybrid **7** selectivity for tumor cells. We performed molecular mechanism studies to evaluate the potential interference of compounds with the p53 pathway, namely, p53–MDM2 interaction and mitosis in HCT116 cells. The antiproliferative activities of compounds were shown to be p53-independent. Compound **7** emerged as an antimitotic agent by inducing the mitotic arrest of colorectal tumor cells, and subsequently, cell death.

## 1. Introduction

**Figure 1:**
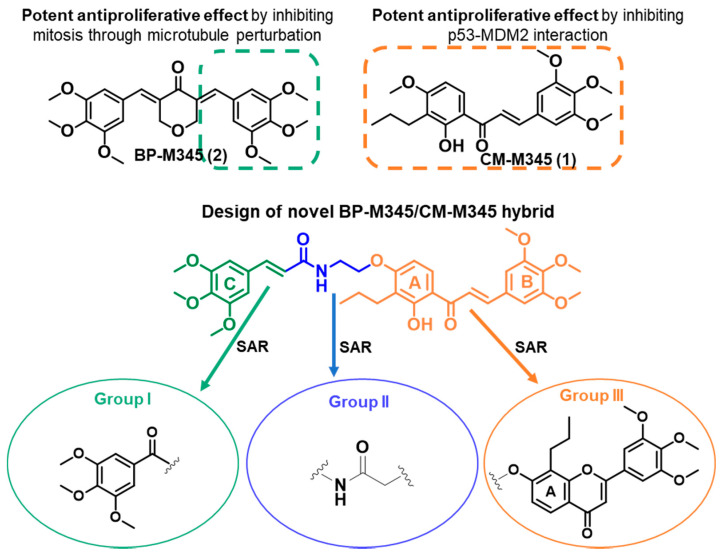
Molecular framework of designed novel hybrid derivatives.

Chalcones, as well as their analogues, diarylpentanoids, have gained high recognition among medicinal chemists due to their wide range of pharmacological potential [1,2,3,4,5]. The antitumor activities of these compounds have been recognized through their interference with several biological targets [6,7,8,9]. Our research group has reported several chalcones and diarylpentanoids with notable growth inhibitory activity in human tumor cell lines [10,11,12,13,14,15]. Particularly, chalcone **CM-M345** (**1**, Figure 1) showed potent antiproliferative activity (2.1 ≤ GI50 ≤ 3.4 μM) in a panel of human tumor cell lines (breast adenocarcinoma MCF-7, non-small cell lung cancer NCI-H460, gastric adenocarcinoma AGS, and colorectal cancer HCT116). It also behaved as an inhibitor of the interaction between the tumor suppressor protein p53 and its negative endogenous regulator, MDM2, in yeast-based assays [16]. More recently, the diarylpentanoid **BP-M345** (**2**, Figure 1) also displayed a potent growth inhibitory effect (0.17 ≤ GI50 ≤ 0.45 μM) on MCF-7 and NCI-H460 cells, as well as on melanoma (A375-C5), showing low cytotoxicity in non-tumor cell lines (fibroblasts, HFF-1, and pulmonary alveolar epithelial HPAEpiC cells) [12,17]. A study of the mechanisms involved in the antiproliferative effect of **BP-M345** revealed that this compound exerts its antiproliferative activity by inhibiting mitosis through microtubule perturbation and cell death induction [17]. Docking studies in the binding site of α,β-tubulin (PDB: 4O2B [18]) suggested that **BP-M345** occupies the colchicine binding site of α,β-tubulin, with one of its 3,4,5-trimethoxyphenyl groups having the same position as the trimethoxyphenyl group (A-ring) of colchicine [17]. These results were in accordance to what was expected, since the presence of a 3,4,5-trimethoxyphenyl group in some microtubule-targeting agents (MTAs), such as colchicine, combretastatin A4, and podophyllotoxin, has been considered as being important for the interaction with tubulin [19].

Molecular hybridization is a useful approach in medicinal chemistry for the development of new therapeutic agents. This strategy proposes the design of a new hybrid molecule with two or more scaffolds connected in a single molecular framework to improve the pharmaceutical profile [20]. Currently, several studies have exploited hybrid molecules integrating scaffolds of diverse small molecules to be screened for a desired pharmacological activity with a good drug profile, particularly as antiproliferative agents [21].

Taking these into account, herein, we report on the design and synthesis of a novel molecular hybrid based on the combination of the subunit 3,4,5-trimethoxycinnamoyl present in **BP-M345** (**2**) and the chalcone **CM-M345** (**1**), as shown in Figure 1. An amide linker was selected as the spacer subunit, considering that cinnamides have been previously reported for their promising antitumor effect [22]. To expand knowledge of the structure–activity relationship (SAR), several analogues of the novel chalcone-trimethoxycinnamide hybrid were planned. The molecular modification strategy involved (i) replacing 3,4,5-trimethoxycinnamoyl with a 3,4,5-trimethoxyphenyl group to evaluate the impact of the enone moiety in the antiproliferative activity (Figure 1), (ii) isosteric substitution of the amide linker with a retro-amide (Figure 1), and (iii) cyclization of the 3-carbon chain present in the chalcone scaffold to evaluate the impact of molecular rigidification (Figure 1). The potential antitumor effects of all synthesized compounds were screened in three human tumor cell lines (A375-C5, MCF-7, and HCT116), and the most promising compounds were selected, aiming to evaluate the mechanism of action related with their cytotoxicity, namely, regarding the p53–MDM2 interaction inhibition, apoptosis induction, and mitotic arrest.

## 2. Results

### 2.1. Synthesis of Chalcone-Trimethoxycinnamide Hybrid **7**

**Scheme 1:**
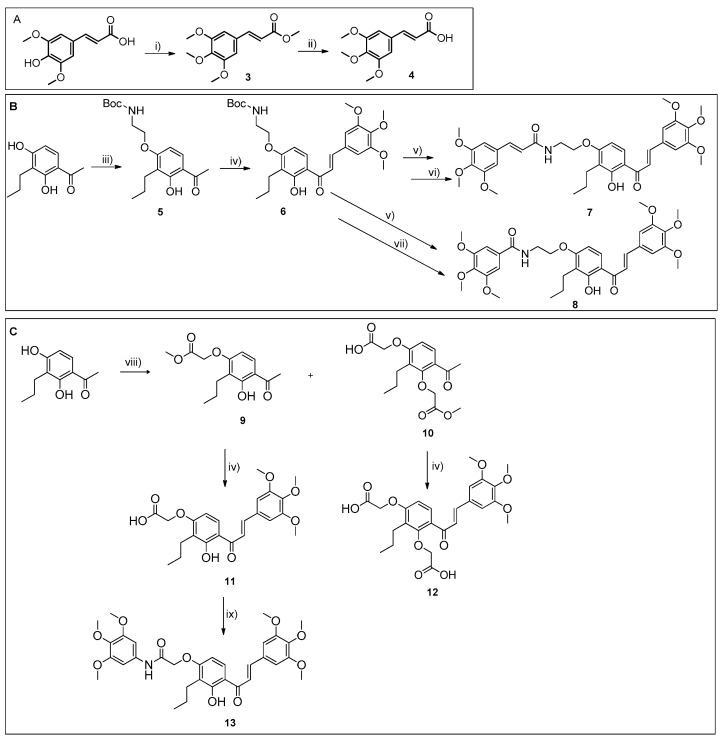
General procedure for the synthesis of chalcone-trimethoxycinnamide (**7**) and chalcone-trimethoxyphenylamide (**8** and **13**) hybrids. (**A**) Synthesis of 3,4,5-trimethoxycinnamnic acid (**4**); (**B**) Synthesis of chalcone-trimethoxycinnamide (**7**) and chalcone-trimethoxyphenylamide (**8**) hybrids; (**C**) Synthesis of chalcone-trimethoxyphenylamide hybrid (**13**). Reaction conditions: (i) (CH_3_)_2_SO_2_, K_2_CO_3_, anhydrous acetone, 65 °C; (ii) HCl:H_2_O, THF, 70 °C; (iii) anhydrous acetone, K_2_CO_3_, 2-(Boc-amino)ethyl bromide; (iv) 3,4,5-trimethoxybenzaldehyde, NaOH, MeOH; (v) TFA; (vi) Hünig’s base, COMU, (*E*)-3-(3,4,5- trimethoxyphenyl)acrylic acid (**4**); (vii) 3,4,5-trimethoxybenzoic acid, Hünig’s base, COMU; (viii) anhydrous acetone, K_2_CO_3_, methyl bromoacetate; (ix) 3,4,5-trimethoxyaniline, Hünig’s base, COMU.

The synthetic approach planned for the synthesis of chalcone-trimethoxycinnamide hybrid **7** consisted of an amidation reaction between a methylated derivative of sinapic acid and the amine derivative of chalcone **CM-M345** (**1**), as highlighted in Scheme 1A,B. The methylated derivative of sinapic acid (**4**) was obtained using a two-step synthesis involving the methylation of sinapic acid to afford methyl (*E*)-3-(3,4,5-trimethoxyphenyl) acrylate (**3**), followed by hydrolysis of the ester group to obtain 3,4,5-trimethoxycinnamic acid (**4**) (Scheme 1A). These two steps were accomplished with good yields (95% and 80%, respectively). The synthesis of the amine derivative of the chalcone **CM-M345** (**1**) was conducted in three steps (Scheme 1B). In the first step, 1-(2,4-dihydroxy-3-propylphenyl)ethan-1-one was submitted to a nucleophilic substitution reaction with 2-(Boc-amino) ethyl bromide in the presence of K_2_CO_3_ and anhydrous acetone, giving rise to the acetophenone derivative *tert*-butyl (2-(4-acetyl-3-hydroxy-2-propylphenoxy)ethyl)carbamate (**5**) with a good yield (90%). After that, chalcone *tert*-butyl (*E*)-(2-(3-hydroxy-2-propyl-4-(3-(3,4,5-trimethoxyphenyl)acryloyl)phenoxy)ethyl)carbamate (**6**) was synthesized via aldol condensation with 3,4,5-trimethoxybenzaldehyde, with a moderate yield (20%). The synthesis of amino chalcone was carried out through the deprotection of the BOC-protected amine through carbamate hydrolysis in acidic conditions with trifluoroacetic acid (TFA) in the presence of dichloromethane (DCM), followed by the reaction of amino chalcone with 3,4,5-trimethoxycinnamic acid (**4**) using the coupling reagent 1-[(1-(cyano-2-ethoxy-2-oxoethylideneaminooxy)-dimethylamino-morpholinomethylene)]methanaminium hexafluorophosphate (COMU) to obtain chalcone-trimethoxycinnamide hybrid **7** with 20% yield.

#### 2.1.1. Synthesis of Compound **7** Analogues with a Chalcone Scaffold (**8** and **13**)

The chalcone-trimethoxyphenylamide hybrid, **8** (analogue of Group I), was obtained using the same synthetic approach as for **7** (Scheme 1B), except for the last step of the reaction. The amidation occurred between amino chalcone and 3,4,5-trimethoxybenzoic acid, using the coupling reagent COMU, giving rise to **8** with 20% yield.

The synthesis of another chalcone-trimethoxyphenylamide hybrid, **13** (an analogue of Group II), resulting from the isosteric substitution of the amide linker by a retro-amide, was conducted using a three-steps pathway (Scheme 1C). Firstly, methyl 2-(4-acetyl-3-hydroxy-2-propylphenoxy)acetate (**9**) was synthetized via a nucleophilic substitution reaction of 1-(2,4-dihydroxy-3-propylphenyl)ethan-1-one with methyl bromoacetate in the presence of K_2_CO_3_ and anhydrous acetone as a solvent with 45% yield. The compound dimethyl 2,2’-((4-acetyl-2-propyl-1,3-phenylene)bis(oxy))diacetate (**10**) was also obtained as a by-product of the reaction in 25% yield. This reaction was followed by an aldol condensation between each synthesized acetophenone (**9** or **10**) with 3,4,5-trimethoxybenzaldehyde. In these cases, the aldol condensation reaction in basic medium also allowed for the hydrolysis of the ester group to obtain (*E*)-2-(3-hydroxy-2-propyl-4-(3-(3,4,5-trimethoxyphenyl)acryloyl)phenoxy)acetic acid (**11**) and (*E*)-2,2’-((2-propyl-4-(3-(3,4,5-trimethoxyphenyl)acryloyl)-1,3-phenylene)bis(oxy))diacetic acid (**12**), respectively, with moderate yields (30% and 35%, respectively). Lastly, chalcone **11** gave rise to hybrid **13** through the reaction with 3,4,5-trimethoxyaniline in the presence of the coupling reagent COMU, with 30% yield.

#### 2.1.2. Synthesis of Compound **7** Analogues with a Flavone Scaffold (**15**, **16**, and **17**)

**Scheme 2:**
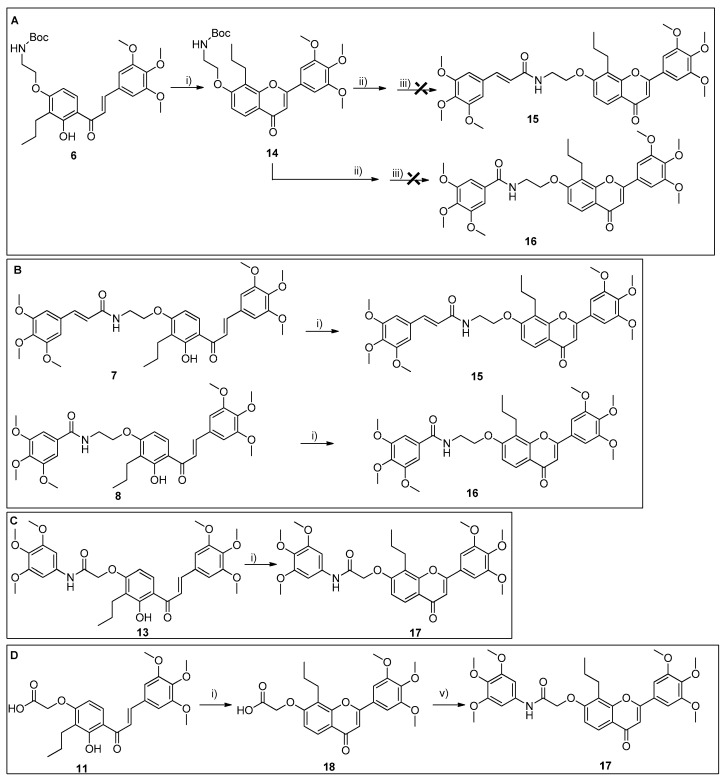
General procedure for the synthesis of flavone-trimethoxycinnamide (**15**) and flavone-trimethoxyphenylamide (**16** and **17**) hybrids. (**A**) Method A for the synthesis of flavone-trimethoxycinnamide (**15**) and flavone-trimethoxyphenylamide (**16**) hybrids. (**B**) Method B for the synthesis of flavone-trimethoxycinnamide (**15**) and flavone-trimethoxyphenylamide (**16**) hybrids. (**C**) Method A for the synthesis of flavone-trimethoxyphenylamide hybrid (**17**). (**D**) Method B for the synthesis of flavone-trimethoxyphenylamide hybrid (**17**). Reaction conditions: (i) I_2_ (cat.), DMSO, reflux, N_2_; (ii) TFA; (iii) 3,4,5-trimethoxycinnamic acid, Hünig’s base, COMU; (v) 3,4,5-trimethoxyaniline, Hünig’s base, COMU.

The synthesis of compound **7** analogues with a flavone scaffold **15** and **16** (analogues of Group III) was firstly planned using chalcone **6** (previously synthesized as described in Section 2.1.1, Scheme 1B) as a building block via a two-step pathway (Method A, Scheme 2A). Firstly, flavone **14** was synthetized via the cyclization of **6** using DMSO/I_2_ with a 30% yield_._ Subsequently, the deprotection of the BOC-protected amine of flavone **14** was accomplished using TFA, and the synthesis of flavone-trimethoxycinnamide **15** and flavone-3,4,5-trimethoxyphenylamide **16** was attempted via a coupling reaction between flavone, and 3,4,5-trimethoxycinnamic acid (**4**) or 3,4,5-trimethoxybenzoic acid, respectively. However, the expected products were not obtained. Therefore, an alternative synthetic pathway was carried out (Method B, Scheme 2B). In this strategy (Scheme 2B), hybrids **15** and **16** were obtained in a one-step synthesis from **7** and **8** hybrids, previously synthesized as described in Section 2.1.1 (Scheme 2B). The cyclization of chalcones into corresponding flavones was carried out using DMSO/I_2_ as a catalyst, giving rise to **15** and **16**, with 35 and 33% yield, respectively.

Considering that the synthesis of **15** and **16** was only accomplished via the direct cyclization of the corresponding chalcone hybrids, the flavone-3,4,5-trimethoxyphenylamide hybrid **17**, with a retro-amide as linker, was also planned using the same strategy (Method A, Scheme 2C), through the direct cyclization of **13** (previously synthesized as described in Section 2.1.1, Scheme 1C), using DMSO/I_2_ as a catalyst, giving rise to **17** with a moderate yield (25%). For comparison purposes, the synthesis of **17** was also conducted in a two-step pathway (Method B, Scheme 2D), consisting of the cyclization of **11** (previously synthesized as described in Section 2.1.1, Scheme 1C) using DMSO/I_2_, affording flavone **18**, which was then submitted to a reaction with 3,4,5-trimethoxyaniline in the presence of the coupling agent COMU, giving rise to **17** with a slightly lower overall yield (17.5%) when compared to Method A. The overall results allowed us to conclude that the synthesis of flavone hybrids was most favorable if the cyclization of chalcone hybrids was the last step of the synthetic pathway.

The structure elucidation of compounds **3**, **4**, and **9** was established using ^1^H and ^13^C nuclear magnetic resonance (NMR) techniques (Figures S1, S2 and S7, Supplementary Materials), and data were in accordance with those previously reported [23,24]. The structural elucidation of new compounds **5**–**8** and **10**–**18** was established on the basis of NMR (Figures S3–S6 and S8–S16—Supplementary Materials) and high-resolution mass spectrometry (HRMS) (Figures S17–S29, Supplementary Materials) techniques. ^13^C NMR assignments were determined using 2D heteronuclear single quantum correlation (HSQC) and heteronuclear multiple bond correlation (HMBC) experiments.

### 2.2. In Vitro Growth Inhibitory Activity of Compounds in Tumor Cell Lines

A sulforhodamine B (SRB) assay was performed to evaluate the in vitro growth inhibitory activity of all synthesized hybrids, as well as intermediates in the human tumor cell lines of melanoma (A375-C5), breast adenocarcinoma (MCF-7), and colorectal carcinoma (HCT116), as well as on the non-tumor Human Pulmonary Alveolar Epithelial cell line (HPAEpiC). The concentration inducing 50% cell growth inhibition (GI_50_), was calculated. Among all of the tested compounds, three (**6**, **7**, and **13**) showed a high growth arrest in all cell lines, and **7** displayed potent growth inhibitory activity against the colorectal tumor HCT116 cell line (Table 1). Interestingly, the three compounds displayed a selective antiproliferative effect on the colorectal tumor cell line, with a GI_50_ ranging from 2.66 μM to 3.26 μM. This suggested that this line may harbor intrinsic characteristics that make it more sensitive to the compounds. Notably, when comparing the GI_50_ values in tumor cells with those in non-tumor cells (HPAEpiC), **7** showed high selectivity for tumor cells, and even higher than that obtained for two previously reported hit compounds, **CM-345** (**1**) and **BP-M345** (**2**). The overall results prompt us to further investigate the mechanism behind the cytotoxic activities of compounds **6**, **7**, and **13** in colorectal tumor cells. Moreover, in silico studies were performed, aiming to screen for drug-likeness.

**Figure 2:**
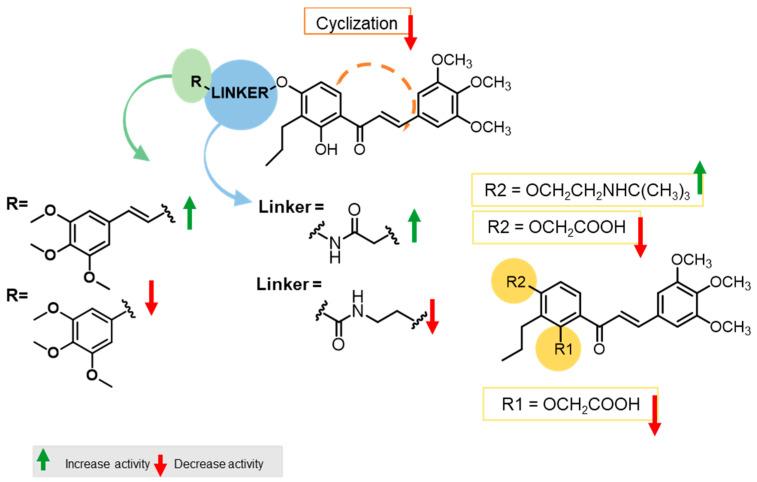
Putative SARs for synthesized compounds with antiproliferative activities.

Considering the antiproliferative effects on tumor cells of all the studied compounds (Table 1), some SAR considerations may be undertaken (Figure 2). Comparing hybrids with the same linker, for chalcone derivatives, the replacement of the 3,4,5-trimethoxycinnamoyl group (**7**) by a 3,4,5-trimethoxyphenyl group (**8**) is associated with a reduction in activity, suggesting that the α,β-unsaturated ketone moiety on the linker is an important feature in the enhancement of the antiproliferative activity (**7**: GI_50_ = 3.26 µM vs. **8**: GI_50_ = 13.49 µM). Nevertheless, this effect is not observed for the flavone derivatives **15** and **16**. Concerning the isosteric substitution of the amide linker by a retro-amide, for chalcone derivatives (**8** vs. **13**), the results suggested that the presence of the carbonyl of the amide group adjacent to the 3,4,5-trimethoxyphenyl ring was less favorable for the activity (**8**: GI_50_ = 13.49 µM vs. **13**: GI_50_ = 2.66 µM), but for flavone derivatives, this isosteric substitution has no effect on the activity. Interestingly, the cyclization of chalcones, resulting in flavone-hybrids (**15**, **16**, and **17**, GI_50_ = 24.68–>75 µM) led to the loss of antiproliferative activity, compared with chalcone-hybrids (**7**, **8**, and **13**, GI_50_ = 2.66–3.26 µM). Additionally, with the screening of the synthetic intermediates, some conclusions can also be inferred (Figure 2). When comparing the antiproliferative activities of chalcone derivatives **11** and **12** with **CM-M345** (**1**), it seems that the substitution of the hydroxyl group by a carboxylic acid side chain in the 4′ position of the chalcone scaffold (**11**) is associated with a remarkable decrease in the growth inhibitory effect (a GI_50_ between 26.87 and 58.34 µM), compared with the compound **CM-M345** (**1**, a GI_50_ between 2.3 and 2.6 µM), and the substitution of both hydroxyl groups at 2′ and 4′ is associated with a complete loss of activity (**12**, GI_50_ > 75 µM). However, the presence of a BOC-protected amine in the 4′ position of the chalcone scaffold is tolerated (**6**, a GI_50_ between 2.81 and 39.81 µM).

### 2.3. Effect of Compounds **6**, **7**, and **13** on the p53–MDM2 Interaction

Since the antiproliferative activity of **CM-M345** (**1**) was associated with a p53–MDM2 inhibitory effect, and compounds **6**, **7**, and **13** displayed a preferential antiproliferative effect on HCT116 p53^+/+^ cells, they were also tested in HCT116 cells, in which p53 has been knocked out (HCT116 p53^−/−^), in order to confirm the contribution of the p53 pathway to the antiproliferative activity (Table 2).

Compounds **6**, **7**, and **13** presented similar antiproliferative activities in both cell lines, suggesting that the growth inhibitory effect of compounds was p53-independent. However, since **CM-M345** (**1**) has been previously reported as a p53-MDM2 inhibitor by our research group [16], the effect of 10 μM of compounds **6**, **7**, and **13** on the p53–MDM2 interaction was analyzed using yeast cell-based assays. The results obtained showed that none of the tested compounds significantly reversed the negative inhibitory growth effect of MDM2 on p53 (data not shown).

### 2.4. Effects of Compounds **6**, **7**, and **13** on HCT116 Cellular Apoptosis

**Figure 3:**
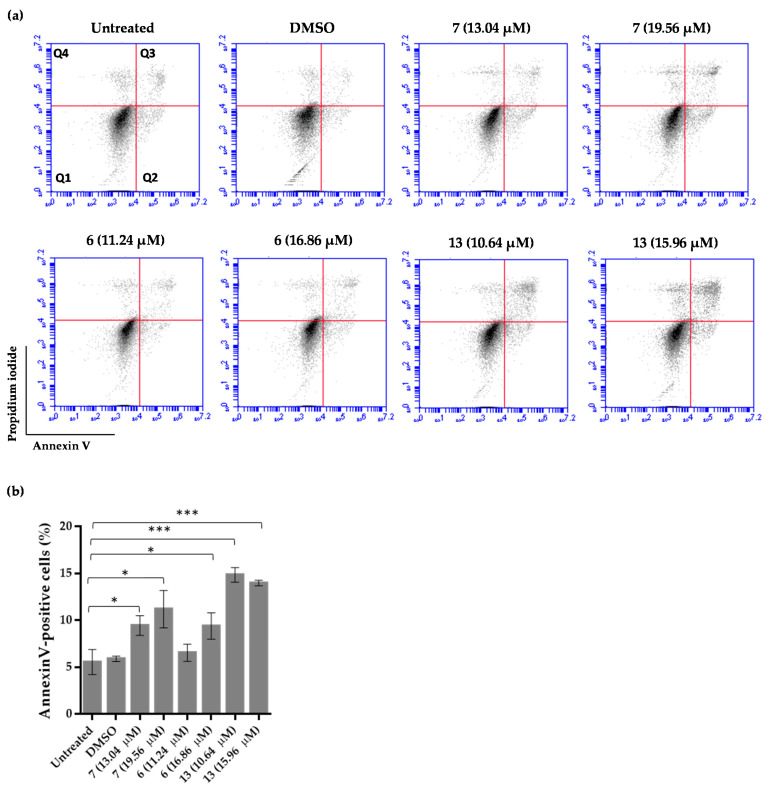
Treatment with **6**, **7**, and **13** induces apoptotic tumor cell death. (**a**) Representative flow cytometry histogram of propidium iodide (PI) and Annexin V (FITC-A) staining in untreated cells and tumor cells treated with **6**, **7**, and **13** at the indicated concentrations, for 48 h. DMSO was used as a control. The quadrants Q were defined as Q1 = live cells (Annexin V-negative/PI-negative), Q2 = early stage of apoptosis (Annexin V-positive/PI-negative), Q3 = late stage of apoptosis (Annexin V-positive/PI-positive) and Q4 = necrosis (Annexin V-negative/PI-positive). (**b**) Quantification of Annexin V-positive cells (early and late apoptosis) from the data shown in (**a**). * *p* < 0.05 and *** *p* < 0.001 via unpaired t-test from three independent experiments.

Double labeling with annexin V-FITC and propidium iodide dyes was used to detect cells going through apoptotic events, using a flow cytometer in order to obtain insights into the mechanism by which compounds **6**, **7**, and **13** generate antiproliferative activity against colorectal carcinoma cells. For that, HCT116 cells were exposed to each compound for 48 h. As shown in Figure 3, the compounds induced apoptosis, when compared to untreated or DMSO-treated control cells. The data suggest that these compounds inhibited the growth of HCT116 tumor cells through apoptosis.

### 2.5. Effect of Compound 7 on HCT116 Mitotic Arrest

**Figure 4:**
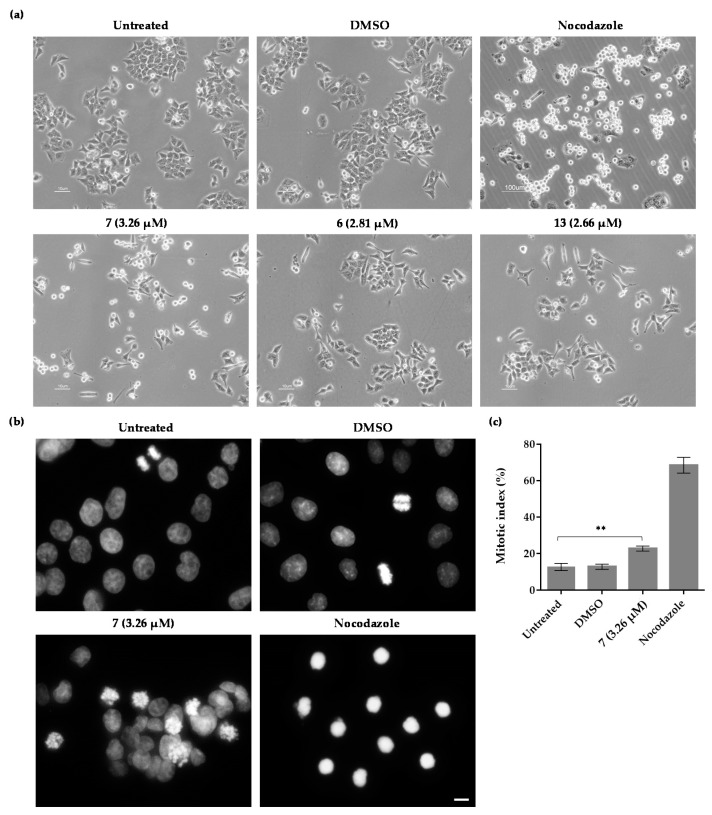
Screening of antimitotic activities of **6**, **7**, and **13**. (**a**) Representative phase-contrast microscopy images of untreated HCT116 cells and cells treated with the indicated compounds for 24 h, showing accumulation of rounded and bright cells after treatment with **7**. Cells treated with DMSO and Nocodazole were used as controls. Bar, 10 µm. (**b**) Representative DAPI staining images of untreated and **7**-treated tumor cells for 24 h, confirming the presence of mitotic cells via DNA condensation. DMSO and Nocodazole were used as controls. Bar, 5 µm. (**c**) Bar graph showing the mitotic index after exposure to **7** for 24 h. ** *p* < 0.01 via unpaired t-test from three independent experiments.

Disruption of cell cycle progression is considered one of the most common cytotoxic mechanisms of anticancer drugs [25]. Moreover, the antimitotic effect of **BP-M345** (**2**) has been previously reported [17]. Therefore, compounds **6**, **7**, and **13** were tested for their capability to induce cell cycle arrest. HCT116 cells were exposed for 24 h to the compounds and then analyzed using phase contrast microscopy for morphological changes. Untreated and dimethyl sulfoxide (DMSO; solvent)-treated cells were used as negative controls. Cell exposure to nocodazole, an antimitotic agent, was used as a positive control. We found an accumulation of bright and rounded cells, reminiscent of mitotic cells, under **7** treatment, which was not observed under **6** or **13** treatment (Figure 4a). DAPI staining of **7**-treated cells to visualize the DNA, confirmed the accumulation of mitotic cells through the observation of condensed chromosomes (Figure 4b), while untreated and DMSO-treated cells showed only a few cells in mitosis, as expected. We thus calculated the mitotic index (MI), and observed a significant increase in MI in **7**-treated cells (22.76 ± 4.30%) compared to the untreated (12.68 ± 1.98%) or DMSO-treated (12.81 ± 1.45%) (Figure 4c). Interestingly, the MI after treatment with **7**, used at GI_50_, was of the same magnitude as the previously reported MI (29.8 ± 5.6%) induced by the parent compound **BP-M345** (**5**) used at 2×GI_50_ [17].

These results suggest that compound **7** exerts its antiproliferative activity on colorectal tumor cells by inducing apoptotic cell death, probably by acting as an antimitotic agent.

### 2.6. Effect of Compound 7 on Mitotic Spindle Morphology

**Figure 5:**
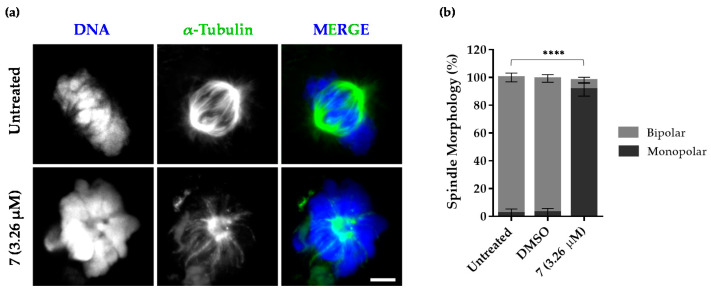
Compound **7** affects mitotic spindle morphology. (**a**) Representative immunofluorescence images of untreated cells (upper panel) with a normal bipolar spindle and compound **7**-treated cells (lower panel) showing a collapsed spindle. Spindle microtubules (green) were stained with anti-α-tubulin antibodies, and DNA (blue) with DAPI. Bar, 5 µm. (**b**) Percentage of bipolar and monopolar spindles in untreated/DMSO and compound **7**-treated cells. **** *p* < 0.0001 untreated monopolar vs. compound **7**-treated monopolar and untreated bipolar vs. compound **7**-treated bipolar via two-way ANOVA with Tukey multiple comparison test from three independent experiments.

To understand the mechanism behind the induction of mitotic arrest after treatment with the compound **7**, we treated HCT116 cells with the GI_50_ of the compound. Twenty-four hours later, we performed an immunofluorescence assay using an anti-α-tubulin antibody to visualize the spindle morphology. Under fluorescence microscopy, we observed that most compound **7**-treated cells showed collapsed mitotic spindles (91.41 ± 4.87%), contrasting with untreated or DMSO-treated cells (2.20 ± 3.13% and 2.86 ± 2.85%, respectively) (Figure 5). Likewise, the percentage of bipolar spindles (the normal morphology of a mitotic spindle) found in compound **7**-treated cells (6.57 ± 2.13%) was significantly lower than in untreated or DMSO-treated cells (97.80 ± 3.13% and 96.43 ± 2.72%, respectively) (Figure 5). This finding indicates that compound **7** induces mitotic delay by interfering with mitotic spindle assembly by generating monopolar-like spindles.

### 2.7. In Silico Prediction of Drug-Likeness of Compounds 6, 7, and 13

The most promising compounds (6, 7, and 13) were screened for drug-likeness properties using in silico models. The drug-likeness was evaluated according to the following chemical features: (i) flexibility, inferred by the number of rotatable bonds (RBs); (ii) unsaturation, inferred by the fraction of carbon sp3 (Fsp3); (iii) solubility, inferred by log S; (iv) polarity, inferred by the polar surface area (PSA); and (v) lipophilicity, inferred by the log P, using the SwissADME web server (SwissADME, http://www.swissadme.ch/, (accessed on 12 May 2023)). For each compound, the above-mentioned molecular descriptors/physicochemical properties were calculated (Table S1, Supporting Material). For each chemical feature, the obtained mean values were compared with the range of values preconized by the drug-likeness guidelines. Based on Table S1, the range of values of MW for compounds **6**, **7**, and **13** (515.60–635.70 g mol^−1^) were higher than the recommended range of values according to the drug-likeness guidelines (150 < MW < 500), as well as the mean MW value (313 g mol^−1^) reported for orally bioavailable cancer drugs [26].

The number of RBs and aromatic character, inferred through unsaturation (Fsp3), and the fraction of aromatic heavy atoms (Far = AHA/HA) are frequently used to infer molecular flexibility. Analyzing these molecular descriptors for all compounds, it was observed that while the number of Fsp3 respects the values preconized by the drug-likeness guidelines, the RB value obtained did not (0.25 < Fps3 < 1; 0 < RB < 9).

The molecular polarity can be determined by the number of HBA, the number of HBD, and the PSA values. Regarding these values (Table S1), it was observed that for compounds **6**, **7**, and **13**, the values of HBD and PSA were in agreement with the literature for synthetic drugs (a mean HBA of 4.8; a mean HBD of 1.6; 20 < PSA < 125 Å^2^) [27].

Lipophilicity and solubility were inferred by log P and Log S, respectively, using different methods (Table S1). Considering the range of values of Log P preconized by the drug-likeness guidelines (0 < Log P < 5) [26], all compounds respect preconized limits, showing that compounds **6**, **7**, and **13** have a higher mean Log P value (4.61 < mean Log P value < 5.15) than anticancer drugs (2.35 < mean Log P value < 3.52). Considering the range of values of Log S preconized by the drug-likeness guidelines (Log S > −4) [28], all compounds might face problems of solubility (−8.35 < mean Log S value < −6.93).

Therefore, taking into account the overall results, in the near future, the optimization of compounds **6**, **7**, and **13** should also consider the improvement of their drug-likeness profile, namely through nanotechnology [29]. In fact, this strategy has been proven to be effective for improving the oral bioavailability of anticancer drugs acting as MTAs with a low solubility, such as paclitaxel [30] and docetaxel [31], and some nanodelivery systems are now under clinical trial.

## 3. Materials and Methods

### 3.1. Chemistry

All reactions were monitored using thin-layer chromatography (TLC). The purification of compounds via flash column chromatography (CC) and preparative TLC was performed using Macherey-Nagel silica gel 60 (0.04–0.063 mm) and Macherey-Nagel silica gel 60 (GF254) plates, respectively. Melting points were obtained under a Köfler microscope and are uncorrected. ^1^H and ^13^C NMR spectra were taken in CDCl_3_, DMSO, or THF at room temperature on Bruker Avance 300 and 400 instruments (300.13 MHz or 400.13 MHz for ^1^H, and 75.47 MHz or 100.63 MHz for ^13^C). Chemical shifts are expressed in δ (ppm) values relative to tetramethylsilane (TMS), used as an internal reference; ^13^C NMR assignments were made using 2D (HSQC and HMBC) NMR experiments (long-range ^13^C-^1^H coupling constants were optimized to 7 Hz). The spectral treatment was executed using the MestReNova v6.0.2-5475 software. The HRMS mass spectra of compounds were performed on an LTQ Orbitrap XL hybrid mass spectrometer (Thermo Fisher Scientific, Bremen, Germany), controlled by LTQ Tune Plus 2.5.5 and Xcalibur 2.1.0. at CEMUP—University of Porto, Portugal. Ions were generated using a Combi MALDI electrospray ionization (ESI) source. All reagents were purchased from Sigma Aldrich. The following materials were synthesized and purified using the described procedures. The HPLC system consisted of a Thermo Scientific SpectraSystem P4000 pump equipped with a degasser, a Thermo Scientific SpectraSystem AS3000 autosampler fitted with a maximum volume of a 100 µL loop, and a Thermo Scientific SpectraSystem UV8000 DAD detector (USA). Data acquisition was performed using ChromQuest 5.0 software, version 3.2.1. The column used in this study was ACE-C18 (150 × 4.6 mm I.D., particle size 5 µm) manufactured by Advanced Chromatography Technologies Ltd. (Aberdeen, Scotland, UK). The mobile phase composition was acetonitrile and water (70:30 *v/v*, for compounds **5**–**10**, and **13**–**18**) or acetonitrile and water (50:50 *v/v*; 0.1% CH_3_CO_2_H, for compounds **11** and **12**, all were HPLC grade solvents obtained from Merck Life Science S.L.U. (Darmstadt, Germany). The flow rate was 1.0 mL/min and the UV detection wavelength was set at 254 nm. Analyses were performed at room temperature in an isocratic mode for 30 min. The peak purity index was determined using a total peak UV-Vis spectra of between 210 and 650 nm, with a step of 4 nm.

#### 3.1.1. Synthesis of Chalcone-Trimethoxycinnamide Hybrid **7**

##### Synthesis of Methyl (*E*)-3-(3,4,5-Trimethoxyphenyl) Acrylate (**3**) 3,4,5-Trimetoxycinnamic Acid (**4**)

Methyl (*E*)-3-(3,4,5-trimethoxyphenyl) acrylate (**3**) and 3,4,5-trimetoxycinnamic acid (**4**) were synthesized (95% and 80% yield, respectively) and characterized according to the described procedure [24]. An experimental description of compounds **3** and **4** is presented in the Supplementary Materials.

##### Synthesis of *tert*-Butyl (2-(4-acetyl-3-hydroxy-2-propylphenoxy)ethyl)carbamate (**5**)

1-(2,4-dihydroxy-3-propylphenyl)ethan-1-one (1 g, 1 Eq, 5 mmol) was dissolved in 15 mL of anhydrous acetone. Then, K_2_CO_3_ (4 g, 5 Eq, 30 mmol) and 2-(Boc-amino)-ethyl bromide (1 g, 1.2 Eq, 6 mmol) were added. The reaction proceeded for 24 h at 70 °C with gentle stirring. After cooling, the reaction mixture was poured onto ice and neutralized with HCl. The obtained solid was filtrated, washed with water, and dried. A white solid (90% yield) corresponding to *tert*-butyl (2-(4-acetyl-3-hydroxy-2-propylphenoxy)ethyl)carbamate (**5**) was obtained.

***tert*-butyl (2-(4-acetyl-3-hydroxy-2-propylphenoxy)ethyl)carbamate** (**5**): 99.3% purity; **mp** 175–177 °C (acetone); **^1^H NMR** (CDCl_3_, 300.13 MHz) *δ*: 12.74 (s, 1H, 2-OH), 7.58 (d, *J* = 8.9 Hz, 1H, H-6), 6.42 (d, *J* = 8.9 Hz, 1H, H-5), 4.91 (sl, 1H, H-3″), 4.09 (t, *J* = 5.2 Hz, 2H, H-1″), 3.56 (q, *J* = 5.6 Hz, 2H, H-2″), 2.63 (t, *J* = 7.5 Hz, 1H, H-1′), 1.58–1.50 (m, 2H, H-2′), 2.56 (s, 3H, 1-COCH_3_), 1.45 (s, 9H, H-6″), 0.94 (t, *J* = 7.4 Hz, 3H, H-3′) ppm. ^**13**^**C NMR** (CDCl_3_. 75.47 MHz) *δ*: 203.2 (1-COCH_3_), 162.5 (C-4), 162.3 (C-2), 156.0 (C-4″), 130.2 (C-6), 118.5 (C-3), 114.6 (C-1), 102.9 (C-5), 79.8 (C-5″), 67.6 (C-1″), 40.2 (C-2″), 28.5 (C-6″), 26.5 (1-COCH_3_), 24.5 (C-1′), 22.1 (C-2′), 14.3 (C-3′) ppm; **HRMS** (ESI^+^): m/z Anal. Calc. for C_18_H_27_NO_5_ (M+Na^+^) 360.17814., found 360.17821.

##### Synthesis of *tert*-Butyl (*E*)-(2-(3-hydroxy-2-propyl-4-(3-(3,4,5-trimethoxyphenyl)acryloyl)phenoxy)ethyl)carbamate (**6**)

To a solution of *tert*-butyl (2-(4-acetyl-3-hydroxy-2-propylphenoxy)ethyl)carbamate (**5**, 200 mg, 1 Eq, 593 µmol) in methanol was added an aqueous solution of sodium hydroxide (40%) to pH 13–14. Then, a solution of 3,4,5-trimethoxybenzaldehyde (349 mg, 3 Eq, 1.78 mmol) in methanol was slowly added to the reaction mixture. The reaction was refluxed for 4 days. After cooling, the reaction was poured onto crushed ice, and the pH was adjusted to 6–8 with a 5 M HCl solution. The obtained solid was filtrated, washed with water, and purified via flash CC (SiO_2_; hexane:ethyl acetate, 7:3). A yellow solid (20% yield) corresponding to tert-butyl(*E*)-(2-(3-hydroxy-2-propyl-4-(3-(3,4,5-trimethoxyphenyl)acryloyl)phenoxy)ethyl)carbamate (**6**) was obtained.

***tert*-butyl(*E*)-(2-(3-hydroxy-2-propyl-4-(3-(3,4,5-trimethoxyphenyl)acryloyl)phenoxy)ethyl)carbamate** (**6**): 98.9% purity; **mp** 196–198 °C (ethyl acetate); **^1^H NMR** (DMSO, 400.13 MHz) *δ*: 13.68 (s, 1H, 2-OH), 8.27 (d, *J* = 9.2 Hz, 1H, H-6), 7.98 (d, *J* = 15.4 Hz, 1H, H-β), 7.79 (d, *J* = 15.4 Hz, 1H, H-α), 7.26 (s, 2H, H-2‴, -6‴), 7.00 (t, *J* = 5.7 Hz, 1H, H-3″), 6.69 (d, *J* = 9.2 Hz, 1H, H-5), 4.10 (t, *J* = 5.6 Hz, 2H, H-1″), 3.87 (s, 6H, 3‴-, 5‴-OCH_3_), 3.72 (s, 3H, 4‴-OCH_3_), 3.35 (t, *J* = 5.6 Hz, 2H, H-2″), 2.59 (t, *J* = 7.5 Hz, 1H, H-1′), 1.52–1.45 (m, 2H, H-2′), 1.39 (s, 9H, H-6″), 0.89 (t, *J* = 7.3 Hz, 3H, H-3″) ppm; ^**13**^**C NMR** (DMSO, 100.63 MHz) *δ*: 192.4 (C=O), 162.7 (C-2), 162.6 (C-4), 155.7 (C-4″), 153.1 (C-3‴,-5‴), 144.7 (C-β), 140.0 (C-4‴), 130.6 (C-6), 130.1 (C-1‴), 120.2 (C-α), 117.1 (C-3), 113.9 (C-1), 106.9 (C-2‴, -6‴), 103.3 (C-5), 77.7 (C-5″), 67.0 (C-1″), 60.2 (4‴-OCH_3_), 56.2 (3‴-, 5‴-OCH_3_), 40.0 (C-2″), 28.2 (C-6″), 24.3 (C-1′), 21.9 (C-2′), 14.0 (C-3′) ppm; **HRMS** (ESI^+^): m/z Anal. Calc. for C_28_H_37_NO_8_ (M+Na^+^) 538.24114., found 538.24191.

##### Synthesis of (*E*)-*N*-(2-(3-Hydroxy-2-propyl-4-((*E*)-3-(3,4,5-trimethoxyphenyl)acryloyl)phenoxy)ethyl)-3-(3,4,5-trimethoxyphenyl)acrylamide (**7**)

*Tert*-butyl(*E*)-(2-(3-hydroxy-2-propyl-4-(3-(3,4,5-trimethoxyphenyl)acryloyl)phenoxy)ethyl)carbamate (**6**, 150 mg, 1 Eq, 291 µmol) was dissolved in 5 mL of dichloromethane, and then 5 mL of TFA was added to the mixture. The reaction proceeded for 26 h with gentle stirring. After completion, the reaction mixture was diluted with dichloromethane, and then a saturated sodium hydrogen carbonate solution was added. The solution was then extracted with ethyl acetate (3 × 50 mL). The organic layers were collected, washed with water, dried over anhydrous sodium sulfate, and concentrated under reduced pressure. The crude obtained was reserved. Then, in a round-bottomed flask, (*E*)-3-(3,4,5-trimethoxyphenyl)acrylic acid (80 mg, 1 Eq, 0.34 mmol) was dissolved in 5 mL of anhydrous dichloromethane and *N*-ethyl-*N*-isopropylpropan-2-amine (87 mg, 0.12 mL, 2 Eq, 0.67 mmol) was added. Then, 50% of the crude material was added to the mixture. After 10 min, COMU (0.22 g, 1.5 Eq, 0.50 mmol) was also added to the mixture. The reaction proceeded for 20 h at room temperature with stirring. After completion, the reaction mixture was poured into ice. The solution was subsequently washed with dichloromethane (3 × 80 mL), with a 1 M HCl solution and saturated NaHCO_3_ solution. The organic phase was dried over anhydrous sodium sulfate, filtered, and solvent evaporated under reduced pressure. The reaction mixture obtained was dissolved in chloroform and purified using TLC (SiO_2_; chloroform:methanol, 95:5). A yellow solid (20% yield) corresponding to (*E*)-*N*-(2-(3-hydroxy-2-propyl-4-((*E*)-3-(3,4,5-trimethoxyphenyl)acryloyl)phenoxy)ethyl)-3-(3,4,5-trimethoxyphenyl)acrylamide (**7**) was obtained.

(*E*)-*N*-(2-(3-hydroxy-2-propyl-4-((*E*)-3-(3,4,5-trimethoxyphenyl)acryloyl)phenoxy)ethyl)-3-(3,4,5-trimethoxyphenyl)acrylamide (7): 98.9% purity; mp 205–207 °C (chloroform); ^1^H NMR (CDCl_3_, 300.13 MHz) *δ*: 13.68 (s, 1H, 2-OH), 8.32–8.27 (m, 1H, 3″-NH-, H-6), 7.99 (d, *J* = 15.4 Hz, 1H, H-β), 7.79 (d, *J* = 15.3 Hz, 1H, H-α), 7.28 (s, 2H, H-2‴, -6‴), 6.89 (s, 2H, H-6″, -10″), 6.87 (d, *J* = 15.8 Hz, 1H, H-β’), 6.62 (d, *J* = 15.8 Hz, 1H, H-α’), 6.73 (d, *J* = 9.2 Hz, 1H, H-5), 4.21 (t, *J* = 5.5 Hz, 2H, H-1″), 3.87 (s, 6H, 3‴-, 5‴-OCH_3_), 3.80 (s, 6H, 7″-, 9″-OCH_3_), 3.72 (s, 3H, 4‴-OCH_3_), 3.78 (sl, 2H, H-2″), 3.67 (s, 3H, 8″-OCH_3_), 2.60 (t, *J* = 7.5 Hz, 1H, H-1′), 1.54–1.44 (m, 2H, H-2′), 0.88 (t, *J* = 7.4 Hz, 3H, H-3″) ppm; ^13^C NMR (CDCl_3_, 75.47 MHz) *δ*: 192.5 (C=O), 165.4 (C-4″), 162.7 (C-2), 162.6 (C-4), 153.2 (C-3‴,-5‴), 153.1 (C-7″, -9″), 144.8 (C-β), 140.0 (C-4‴), 139.1 (C-4″), 130.5 (C-6), 130.5 (C-1‴), 130.1 (C-5″), 121.3 (C-α’), 120.4 (C-α), 117.2 (C-3), 114.0 (C-1), 106.9 (C-2‴, -6‴), 104.9 (C-6″, -10″), 103.6 (C-β’), 103.4 (C-5), 66.9 (C-1″), 60.2 (4‴-OCH_3_), 60.1 (8″-OCH_3_), 56.2 (3‴-, 5‴-OCH_3_), 56.0 (7″-, 9″-OCH_3_), 38.7 (C-2″), 24.0 (C-1′), 21.6 (C-2′), 14.1 (C-3′) ppm; HRMS (ESI^+^): m/z Anal. Calc. for C_35_H_41_NO_10_ (M+Na^+^) 658.26227., found 658.26343.

#### 3.1.2. Synthesis of Compound **7** Analogues with a Chalcone Scaffold (**8** and **13**)

##### Synthesis of (*E*)-*N*-(2-(3-Hydroxy-2-propyl-4-(3-(3,4,5-trimethoxyphenyl)acryloyl)phenoxy)ethyl)-3,4,5-trimethoxybenzamide (**8**)

*Tert*-butyl(*E*)-(2-(3-hydroxy-2-propyl-4-(3-(3,4,5-trimethoxyphenyl)acryloyl)phenoxy)ethyl)carbamate (**6**, 150 mg, 1 Eq, 291 µmol) was dissolved in 5 mL of dichloromethane. Then, 5 mL of TFA was added to the mixture. The reaction proceeded for 26 h with gentle stirring. After completion, the reaction mixture was diluted with dichloromethane, and then a saturated sodium hydrogen carbonate solution was added. The solution was then extracted with ethyl acetate (3 × 50 mL). The organic layers were collected, washed with water, dried over anhydrous sodium sulfate, and concentrated under reduced pressure. The crude obtained was reserved. Then, in a round-bottomed flask, 3,4,5-trimethoxybenzoic acid (85 mg, 1 Eq, 0.40 mmol) was dissolved in 5 mL of anhydrous dichloromethane and *N*-ethyl-*N*-isopropylpropan-2-amine (0.10 g, 0.14 mL, 2 Eq, 0.80 mmol) was added. Then, 50% of the crude material was added to the mixture. After 10 min, COMU (0.26 g, 1.5 Eq, 0.60 mmol) was also added to the mixture. The reaction proceeded for 3 h at room temperature with stirring. After completion, the reaction mixture was poured onto ice. The solution was subsequently washed with 1M HCl solution and saturated NaHCO_3_ solution. The organic phase was dried over anhydrous sodium sulfate, filtered, and solvent evaporated under reduced pressure. The reaction mixture obtained was purified via TLC (SiO_2_; dichloromethane:methanol; 97:3). A light-yellow solid (20% yield) corresponding to (*E*)-*N*-(2-(3-hydroxy-2-propyl-4-(3-(3,4,5-trimethoxyphenyl)acryloyl)phenoxy)ethyl)-3,4,5-trimethoxybenzamide (**8**) was obtained.

**(*E*)-*N*-(2-(3-hydroxy-2-propyl-4-(3-(3,4,5-trimethoxyphenyl)acryloyl)phenoxy)ethyl)-3,4,5-trimethoxybenzamide** (**8**): 97.8% purity; **mp** 201–203 °C (chloroform); **^1^H NMR** (DMSO, 400.13 MHz) *δ*: 13.67 (s, 1H, 2-OH), 8.63 (t, *J* = 5.5 Hz, 1H, 3″-NH-), 8.28 (d, *J* = 9.1 Hz, 1H, H-6), 7.98 (d, *J* = 15.4 Hz, 1H, H-β), 7.29 (d, *J* = 15.3 Hz, 1H, H-α), 7.26 (s, 2H, H-2‴, -6‴), 7.20 (s, 2H, H-6″, -10″), 6.76 (d, *J* = 9.0 Hz, 1H, H-5), 4.28 (t, *J* = 5.6 Hz, 2H, H-1″), 3.87 (s, 6H, 3‴-, 5‴-OCH_3_), 3.82 (s, 6H, 7″-, 9″-OCH_3_), 3.72 (s, 3H, 4‴-OCH_3_), 3.69–3.66 (m, 2H, H-2″), 3.68 (s, 3H, 8″-OCH_3_), 2.58 (t, *J* = 7.3 Hz, 1H, H-1′), 1.49–1.40 (m, 2H, H-2′), 0.82 (t, *J* = 7.3 Hz, 3H, H-3′) ppm; ^**13**^**C NMR** (DMSO, 100.63) *δ*: 192.4 (C=O), 166.0 (C-4″), 162.7 (C-2), 162.6 (C-4), 153.1 (C-3‴,-5‴), 152.5 (C-7″, -9″), 144.7 (C-β), 140.0 (C-4‴), 140.0 (C-4″), 130.6 (C-6), 130.6 (C-1‴), 129.5 (C-5″), 120.2 (C-α), 117.1 (C-3), 113.9 (C-1), 106.9 (C-2‴, -6‴), 104.8 (C-6″, -10″), 103.4 (C-5), 66.5 (C-1″), 60.2 (4‴-OCH_3_), 60.1 (8″-OCH_3_), 56.2 (3‴-, 5‴-OCH_3_), 56.0 (7″-, 9″-OCH_3_), 38.9 (C-2″), 23.9 (C-1′), 21.0 (C-2′), 14.0 (C-3′) ppm; **HRMS** (ESI^+^): m/z Anal. Calc. for C_33_H_39_NO_10_ (M+Na^+^) 632.24662, found 632.24594.

##### Synthesis of Methyl 2-(4-Acetyl-3-hydroxy-2-propylphenoxy)acetate (**9**) and Dimethyl 2,2’-((4-acetyl-2-propyl-1,3-phenylene)bis(oxy))diacetate (**10**)

1-(2,4-dihydroxy-3-propylphenyl)ethan-1-one (1 g, 1 Eq, 5.149 mmol) was dissolved in 5 mL of anhydrous acetone. Then, K_2_CO_3_ (711.5 mg, 1 Eq, 5.149 mmol) and methyl 2-bromoacetate (945.1 mg, 584.9 µL, 1.2 Eq, 6.178 mmol) were added. The reaction proceeded for 24 h at 70 °C with gentle stirring. After cooling, the reaction mixture was poured onto ice and neutralized with HCl. The obtained solid was filtrated, washed with water, and dried. A white solid (45% yield) corresponding to methyl 2-(4-acetyl-3-hydroxy-2-propylphenoxy)acetate (**9**) was obtained. The aqueous phase was then extracted with dichloromethane (3 × 50 mL). The organic layers were collected, washed with water, dried over anhydrous sodium sulfate, concentrated under reduced pressure, and purified using flash CC (SiO_2_; n-hexane:ethyl acetate, 7:3). A gum (25% yield) corresponding to dimethyl 2,2’-((4-acetyl-2-propyl-1,3-phenylene)bis(oxy))diacetate (**10**) was obtained.

The experimental description of compound **9** is presented in the Supplementary Materials.

**Dimethyl 2,2’-((4-acetyl-2-propyl-1,3-phenylene)bis(oxy))diacetate** (**10**): 99.7% purity; **mp** N.D. (gum); **^1^H NMR** (CDCl_3_, 300.13 MHz) *δ*: 7.50 (d, *J* = 8.7 Hz, 1H, H-6), 6.53 (d, *J* = 8.7 Hz, 1H, H-5), 4.69 (s, 2H, H-1″), 4.47 (s, 2H, H-1‴), 3.81 (s, 3H, H-3″), 3.79 (s, 3H, H-3‴), 2.69 (t, *J* = 7.8 Hz, 2H, H-1′), 1.63–1.53 (m, 2H, H-2′), 2.56 (s, 3H, 1-COCH_3_), 0.97 (t, *J* = 7.4 Hz, 3H, H-3′) ppm; **^13^C NMR** (CDCl_3_. 75.47 MHz) *δ*: 198.9 (1-COCH_3_), 169.2 (C-2‴), 168.9 (C-2″), 160.1 (C-4), 156.8 (C-2), 129.2 (C-6), 126.8 (C-1), 126.4 (C-3), 107.1 (C-5), 71.8 (C-1‴), 65.3 (C-1″), 52.4 (C-3‴), 52.2 (C-3″), 30.0 (1-COCH_3_), 26.0 (C-1′), 22.9 (C-2′), 14.5 (C-3′) ppm; **HRMS** (ESI^+^): m/z Anal. Calc. for C_17_H_22_O_7_ (M+Na^+^) 361.12577, found 361.12594.

##### Synthesis of (*E*)-2-(3-Hydroxy-2-propyl-4-(3-(3,4,5-trimethoxyphenyl)acryloyl)phenoxy)acetic Acid (**11**)

To a solution of methyl 2-(4-acetyl-3-hydroxy-2-propylphenoxy)acetate (**9**, 200 mg, 1 Eq, 751 µmol) in methanol was added an aqueous solution of 40% sodium hydroxide to pH 13–14. Then, a solution of 3,4,5-trimethoxybenzaldehyde (442 mg, 3 Eq, 2.25 mmol) in methanol was slowly added to the reaction mixture. The reaction was refluxed for 2 days. After cooling, the reaction was poured into crushed ice and neutralized with a 5 M HCl solution. The solution was then extracted with ethyl acetate (3 × 50 mL). The organic layers were collected, washed with water, and dried over anhydrous sodium sulfate. The solvent was evaporated to dryness, and the obtained reaction mixture was purified via flash CC (SiO_2_; chloroform:methanol, 95:5). A yellow solid (30% yield) corresponding to (*E*)-2-(3-hydroxy-2-propyl-4-(3-(3,4,5-trimethoxyphenyl)acryloyl)phenoxy)acetic acid (**11**) was obtained.

**(*E*)-2-(3-hydroxy-2-propyl-4-(3-(3,4,5-trimethoxyphenyl)acryloyl)phenoxy)acetic acid** (**11**): 98.7% purity; **mp** 200–202 °C (chloroform); **^1^H NMR** (DMSO, 400.13 MHz) *δ*: 13.39 (s, 1H, 2-OH), 12.73 (s, 1H, 2″-OH), 7.79 (d, *J* = 15.6 Hz, 1H, H-β), 7.75 (d, *J* = 9.2 Hz, 1H, H-6), 7.56 (d, *J* = 8.8 Hz, 1H, H-5), 7.45 (d, *J* = 15.4 Hz, 1H, H-α), 6.86 (s, 2H, H-2‴, -6‴), 4.72 (sl, 2H, H-1″), 3.92 (s, 6H, 3‴-, 5‴-OCH_3_), 3.90 (s, 3H, 4‴-OCH_3_), 2.73–2.66 (m, 2H, H-1′), 1.63–1.53 (m, 2H, H-2′), 0.94 (t, *J* = 7.7 Hz, 3H, H-3′) ppm; ^**13**^**C NMR** (DMSO, 100.63 MHz) *δ*: 192.4 (C=O), 172.8 (C-2″), 163.7 (C-2), 162.5 (C-4), 153.7 (C-3‴,-5‴), 145.0 (C-β), 140.8 (C-4‴), 130.1 (C-5), 130.4 (C-1‴), 129.0 (C-6), 119.7 (C-α), 119.4 (C-3), 115.3 (C-1), 106.1 (C-2‴, -6‴), 65.1 (C-1″), 61.2 (4‴-OCH_3_), 56.4 (3‴-, 5‴-OCH_3_), 24.7 (C-1′), 22.0 (C-2′), 14.4 (C-3′) ppm; **HRMS** (ESI^+^): m/z Anal. Calc. for C_23_H_26_O_10_ (M+H^+^) 431.17004, found 431.16961.

##### Synthesis of (*E*)-2,2’-((2-Propyl-4-(3-(3,4,5-trimethoxyphenyl)acryloyl)-1,3-phenylene)bis(oxy))diacetic Acid (**12**)

To a solution of methyl 2-(4-acetyl-3-hydroxy-2-propylphenoxy)acetate (**10**, 200 mg, 1 Eq, 751 µmol) in methanol was added an aqueous solution of 40% sodium hydroxide to pH 13–14. Then, a solution of 3,4,5-trimethoxybenzaldehyde (442 mg, 3 Eq, 2.25 mmol) in methanol was slowly added to the reaction mixture. The reaction was refluxed for 4 days. After cooling, the reaction was poured onto crushed ice and the pH was adjusted to 6–8, with a 5 M HCl solution. The obtained solid was filtrated, washed with water, dried, and purified via flash CC (SiO_2_; chloroform:methanol, 9:1). A yellow solid (35% yield) corresponding to (*E*)-2,2’-((2-propyl-4-(3-(3,4,5-trimethoxyphenyl)acryloyl)-1,3-phenylene)bis(oxy))diacetic acid (**12**) was obtained.

**(*E*)-2,2’-((2-propyl-4-(3-(3,4,5-trimethoxyphenyl)acryloyl)-1,3-phenylene)bis(oxy))diacetic acid** (**12**): 98.7% purity; **mp** 212–214 °C (chloroform); **^1^H NMR** (DMSO, 400.13 MHz) *δ*: 7.55–7.52 (m, 3H, H-α,-β, -6), 7.10 (s, 2H, H-2‴’, -6‴’), 6.79 (d, *J* = 8.8 Hz, 1H, H-5), 4.80 (s, 2H, H-1″), 4.35 (s, 2H, H-1‴), 3.84 (s, 6H, 3‴’-, 5‴’-OCH_3_), 3.70 (s, 3H, 4‴’-OCH_3_), 2.69 (t, *J* = 7.4 Hz, 1H, H-1′), 1.60–1.51 (m, 2H, H-2′), 0.93 (t, *J* = 7.4 Hz, 3H, H-3′) ppm; ^**13**^**C NMR** (DMSO, 100.63 MHz) *δ*: 190.3 (C=O), 170.0 (C-2″), 169.5 (C-2‴), 159.6 (C-4), 156.3 (C-2), 153.1 (C-3‴’, -5‴’), 144.0 (C-β), 139.6 (C-4‴’), 130.1 (C-1‴’), 129.0 (C-6), 125.4 (C-α), 125.1 (C-1), 124.5 (C-3), 107.4 (C-5), 106.2 (C-2‴’, -6‴’), 71.4 (C-1‴), 65.0 (C-1″), 60.1 (4‴’-OCH_3_), 56.0 (3‴’-, 5‴’-OCH_3_), 25.4 (C-1′), 22.3 (C-2′), 14.3 (C-3′) ppm; **HRMS** (ESI^+^): m/z Anal. Calc. for C_25_H_28_O_10_ (M+Na^+^) 511.15747, found 511.15885.

##### Synthesis of (*E*)-2-(3-Hydroxy-2-propyl-4-(3-(3,4,5-trimethoxyphenyl)acryloyl)phenoxy)-*N*-(3,4,5-trimethoxyphenyl)acetamide (**13**)

(*E*)-2-(3-hydroxy-2-propyl-4-(3-(3,4,5-trimethoxyphenyl)acryloyl)phenoxy)acetic acid (**11**, 85 mg, 1 Eq, 0.20 mmol) was dissolved in 5 mL of anhydrous dichloromethane and *N*-ethyl-*N*-isopropylpropan-2-amine (51 mg, 69 µL, 2 Eq, 0.39 mmol) was added. Then, 3,4,5-trimethoxyaniline (43 mg, 1.2 Eq, 0.24 mmol) was added to the mixture. After 10 min, COMU (0.13 g, 1.5 Eq, 0.30 mmol) was also added to the mixture. The reaction proceeded for 3 h at room temperature with stirring. After completion, the reaction mixture was poured onto ice. The solution was subsequently washed with a 1 M HCl solution and a saturated NaHCO_3_ solution, and dried over anhydrous sodium sulfate. The solvent was evaporated to dryness, and the obtained reaction mixture was purified via TLC (SiO_2_; dichloromethane:methanol; 97:3). A yellow solid (30% yield) corresponding to (*E*)-2-(3-hydroxy-2-propyl-4-(3-(3,4,5-trimethoxyphenyl)acryloyl)phenoxy)-*N*-(3,4,5-trimethoxyphenyl)acetamide (**13**) was obtained.

**(*E*)-2-(3-hydroxy-2-propyl-4-(3-(3,4,5-trimethoxyphenyl)acryloyl)phenoxy)-N-(3,4,5-trimethoxyphenyl)acetamide** (**13**)**:** 95.9% purity; **mp** 199–201 °C (chloroform); **^1^H NMR** (DMSO, 400.13 MHz) *δ*: 13.66 (s, 1H, 2-OH), 10.18 (s, 1H, 3″-NH-), 8.27 (d, *J* = 9.2 Hz, 1H, H-6), 7.95 (d, *J* = 15.4 Hz, 1H, H-β), 7.80 (d, *J* = 15.3 Hz, 1H, H-α), 7.25 (s, 2H, H-2‴, -6‴), 7.01 (s, 2H, H-5″, -9″), 6.61 (d, *J* = 9.2 Hz, 1H, H-5), 4.90 (s, 2H, H-1″), 3.86 (s, 6H, 3‴-, 5‴-OCH_3_), 3.74 (s, 6H, 6″-, 8″-OCH_3_), 3.72 (s, 3H, 4‴-OCH_3_), 3.62 (s, 3H, 7″-OCH_3_), 2.66 (t, *J* = 7.6 Hz, 1H, H-1′), 1.58–1.51 (m, 2H, H-2′), 0.94 (t, *J* = 7.4 Hz, 3H, H-3″) ppm; ^**13**^**C NMR** (DMSO, 100.63 MHz) *δ*: 192.4 (C=O), 165.8 (C-2″), 162.6 (C-2), 162.1 (C-4), 153.1 (C-3‴,-5‴), 152.8 (C-6″, -8″), 145.0 (C-β), 140.0 (C-4‴), 134.6 (C-4″), 133.7 (C-7″), 130.5 (C-6), 130.5 (C-1‴), 120.2 (C-α), 117.4 (C-3), 114.2 (C-1), 106.8 (C-2‴, -6‴), 103.4 (C-5), 97.0 (C-5″, -9″), 67.2 (C-1″), 60.1 (4″-, 4‴-OCH_3_), 56.2 (3‴-, 5‴-OCH_3_), 55.7 (6″-, 8″-OCH_3_), 24.2 (C-1′), 21.4 (C-2′), 14.2 (C-3′) ppm; **HRMS** (ESI^+^): m/z Anal. Calc. for C_32_H_37_NO_10_ (M+Na^+^) 618.23097, found 618.23023.

#### 3.1.3. Synthesis of Compound **7** Analogues with a Flavone Scaffold (**15**, **16**, and **18**)

##### Synthesis of *tert*-Butyl (2-((4-oxo-8-propyl-2-(3,4,5-trimethoxyphenyl)-4*H*-chromen-7-yl)oxy)ethyl)carbamate (**14**)

To a solution of *tert*-butyl (*E*)-(2-(3-hydroxy-2-propyl-4-(3-(3,4,5-trimethoxyphenyl)acryloyl)phenoxy)ethyl)carbamate (**6**, 190 mg, 1 Eq, 369 µmol) in DMSO (10mL/mmol of chalcone), I_2_ (935 µg, 0.01 Eq, 3.69 µmol) was added. The mixture was heated at reflux for 24 h. Then, the mixture was poured into ice and a sodium thiosulfate solution (10%) was added. The obtained mixture was extracted with ethyl acetate, washed with water, and then dried over anhydrous sodium sulfate. The solvent was evaporated to dryness, and the obtained reaction mixture was purified via TLC (SiO_2_, chloroform:methanol; 95:5). A white solid (30% yield) corresponding to *tert*-butyl (2-((4-oxo-8-propyl-2-(3,4,5-trimethoxyphenyl)-4*H*-chromen-7-yl)oxy)ethyl)carbamate (14) was obtained.

***tert*-butyl (2-((4-oxo-8-propyl-2-(3,4,5-trimethoxyphenyl)-4*H*-chromen-7-yl)oxy)ethyl)carbamate** (14): 98.9% purity; **mp** 191–193 °C (ethyl acetate); **^1^H NMR** (DMSO, 400.14 MHz) *δ*: 7.88 (d, *J* = 8.9 Hz, 1H, H-5), 7.34 (s, 2H, H-2‴, -6‴), 7.20 (d, *J* = 8.9 Hz, 1H, H-6), 7.07 (s, 1H, H-3), 7.02 (t, *J* = 5.8 Hz, 1H, 3″-NH-), 4.18 (t, *J* = 5.5 Hz, 2H, H-1″), 3.91 (s, 6H, 3‴-, 5‴-OCH_3_), 3.90–3.67 (m, 2H, H-2″), 3.76 (s, 3H, 4‴-OCH_3_), 2.74 (t, *J* = 7.1 Hz, 2H, H-1′), 1.68–1.61 (m, 2H, H-2′), 1.38 (s, 9H, H-6″), 0.97 (t, *J* = 7.3 Hz, 3H, H-3′) ppm; **^13^C NMR** (DMSO, 100.63 MHz) *δ*: 177.0 (C=O), 161.8 (C-2), 160.6 (C-7), 155.7 (C-4″), 154.4 (C-8a), 153.3 (C-3‴, -5‴), 140.4 (C-4‴), 126.8 (C-1‴), 123.8 (C-5), 118.2 (C-4a), 117.2 (C-8), 110.4 (C-6), 106.1 (C-3), 103.5 (C-2‴, -6‴), 77.7 (C-5″), 67.6 (C-1″), 60.2 (4‴-OCH_3_), 56.3 (C-2″), 56.1 (3‴-, 5‴-OCH_3_), 28.2 (6″-CH_3_), 24.8 (C-1′), 22.1 (C-2′), 14.2 (C-3′) ppm; **HRMS** (ESI^+^): m/z Anal. Calc. for C_28_H_35_NO_8_ (M+H^+^) 514.24354, found 514.24311.

##### Synthesis of (*E*)-*N*-(2-((4-oxo-8-Propyl-2-(3,4,5-trimethoxyphenyl)-4*H*-chromen-7-yl)oxy)ethyl)-3-(3,4,5-trimethoxyphenyl)acrylamide (**15**)

To a solution of (*E*)-*N*-(2-(3-hydroxy-2-propyl-4-((*E*)-3-(3,4,5-trimethoxyphenyl)acryloyl)phenoxy)ethyl)-3-(3,4,5-trimethoxyphenyl)acrylamide (**7**, 10 mg, 1 Eq, 16 μmol) in DMSO (10mL/mmol of chalcone), I_2_ (40 μg, 0.01 Eq, 0.16 μmol) was added. The mixture was heated at reflux for 2 h. Then, the mixture was poured into ice and a sodium thiosulfate solution (10%) was added. The obtained mixture was extracted with ethyl acetate, washed with water, and then dried over anhydrous sodium sulfate. The solvent was evaporated to dryness, and the obtained reaction mixture was purified via TLC (SiO_2_, chloroform:methanol; 95:5). A white solid (35% yield) corresponding to (*E*)-*N*-(2-((4-oxo-8-propyl-2-(3,4,5-trimethoxyphenyl)-4*H*-chromen-7-yl)oxy)ethyl)-3-(3,4,5-trimethoxyphenyl)acrylamide (15) was obtained.

**(*E*)-*N*-(2-((4-oxo-8-propyl-2-(3,4,5-trimethoxyphenyl)-4*H*-chromen-7-yl)oxy)ethyl)-3-(3,4,5-trimethoxyphenyl)acrylamide** (15): 98.4% purity; **mp** 198–199 °C (chloroform); **^1^H NMR** (CDCl_3_, 400.13 MHz) *δ*: 8.09 (d, *J* = 8.9 Hz, 1H, H-5), 7.59 (d, *J* = 15.8 Hz, 1H, H-β), 7.15 (s, 2H, H-2‴, -6‴), 7.02 (d, *J* = 8.9 Hz, 1H, H-6), 6.73 (s, 2H, H-6″, -10″), 6.72 (s, 1H, H-3), 6.32 (d, *J* = 15.5 Hz, 1H, H-α), 5.97 (t, *J* = 5.7 Hz, 1H, 3″-NH-), 4.28 (t, *J* = 5.2 Hz, 2H, H-1″), 3.95 (s, 6H, 3‴-, 5‴-OCH_3_), 3.94 (s, 3H, 4‴-OCH_3_), 3.88 (s, 6H, 7″-, 9″-OCH_3_), 3.87 (s, 3H, 8″-OCH_3_), 3.95–3.90 (m, 2H, H-2″), 2.98 (t, *J* = 7.5 Hz, 1H, H-1′), 1.78–1.72 (m, 2H, H-2′), 1.06 (t, *J* = 7.4 Hz, 3H, H-3′) ppm; ^**13**^**C NMR** (CDCl_3_, 100.63 MHz) *δ*: 178.9 (C=O), 166.2 (C-4″), 162.9 (C-2), 160.6 (C-7), 155.4 (C-8a), 153.8 (C-3‴,-5‴), 153.6 (C-7″, -9″), 142.1 (C-β), 140.0 (C-4‴), 139.2 (C-8″), 130.4 (C-5″), 127.4 (C-1‴), 124.7 (C-5), 119.4 (C-4a), 119.3 (C-α), 118.7 (C-8), 109.9 (C-6), 106.6 (C-3), 105.2 (C-2‴, -6‴), 103.5 (C-6″, -10″), 67.8 (C-1″), 61.2 (4‴-OCH_3_), 61.1 (4″-OCH_3_), 56.4 (3‴-, 5‴-OCH_3_), 56.3 (7″-, 9″-OCH_3_), 39.5 (C-2″), 25.7 (C-1′), 22.8 (C-2′), 14.7 (C-3′) ppm; **HRMS** (ESI^+^): m/z Anal. Calc. for C_35_H_39_NO_10_ (M+CH_3_OHH^+^) 666.29089, found 666.29022.

##### Synthesis of 3,4,5-Trimethoxy-*N*-(2-((4-oxo-8-propyl-2-(3,4,5-trimethoxyphenyl)-4*H*-chromen-7-yl)oxy)ethyl)benzamide (**16**)

To a solution of (*E*)-*N*-(2-(3-hydroxy-2-propyl-4-(3-(3,4,5-trimethoxyphenyl)acryloyl)phenoxy)ethyl)-3,4,5-trimethoxybenzamide (**8**, 10 mg, 1 Eq, 16 μmol) in DMSO (10mL/mmol of chalcone), I_2_ (42 μg, 0.01 Eq, 0.16 μmol) was added. The mixture was heated at reflux for 2 h. Then, the mixture was poured into ice and a sodium thiosulfate solution (10%) was added. The obtained mixture was extracted with ethyl acetate, washed with water, and then dried over anhydrous sodium sulfate. The solvent was evaporated to dryness and the reaction mixture was purified via TLC (SiO_2_, chloroform:methanol; 95:5). A white solid (33% yield) corresponding to 3,4,5-trimethoxy-*N*-(2-((4-oxo-8-propyl-2-(3,4,5-trimethoxyphenyl)-4H-chromen-7-yl)oxy)ethyl)benzamide (**16**) was obtained.

**3,4,5-trimethoxy-*N*-(2-((4-oxo-8-propyl-2-(3,4,5-trimethoxyphenyl)-4*H*-chromen-7-yl)oxy)ethyl)benzamide** (**16**): 95.8% purity; **mp** 196–199 °C (chloroform); **^1^H NMR** (DMSO, 400.13 MHz) *δ*: 8.64 (t, *J* = 5.6 Hz, 1H, 3″-NH-), 7.90 (d, *J* = 8.8 Hz, 1H, H-5), 7.33 (s, 2H, H-2‴, -6‴), 7.27 (d, *J* = 8.9 Hz, 1H, H-6), 7.07 (s, 2H, H-6″, -10″), 7.07 (s, 1H, H-3), 4.02 (t, *J* = 5.2 Hz, 2H, H-1″), 3.90 (s, 6H, 3‴-, 5‴-OCH_3_), 3.81 (s, 6H, 7″-, 9″-OCH_3_), 3.75 (s, 3H, 4‴-OCH_3_), 3.69 (s, 3H, 8″-OCH_3_), 3.51–3.46 (m, 2H, H-2″), 2.89 (t, *J* = 7.4 Hz, 2H, H-1′), 1.68–1.59 (m, 2H, H-2′), 0.89 (t, *J* = 7.4 Hz, 3H, H-3′) ppm; **^13^C NMR** (DMSO, 100.63 MHz) *δ*: 177.4 (C=O), 166.1 (C-4″), 162.5 (C-2), 160.2 (C-7), 155.9 (C-8a), 154.5 (C-3‴,-5‴), 154.4 (C-7″, -9″), 140.4 (C-4‴), 140.2 (C-8″), 129.8 (C-5″), 126.8 (C-1‴), 124.3 (C-5), 119.8 (C-4a), 117.3 (C-8), 109.7 (C-6), 106.0 (C-3), 104.7 (C-6″, -10″), 103.5 (C-2‴, -6‴), 67.6 (C-1″), 61.4 (4‴-OCH_3_), 60.0 (8″-OCH_3_), 56.1 (3‴-, 5‴-OCH_3_), 55.9 (7″-, 9″-OCH_3_), 38.9 (C-2″), 30.7 (C-1′), 22.1 (C-2′), 14.1 (C-3′) ppm; **HRMS** (ESI^+^): m/z Anal. Calc. for C_33_H_37_NO_10_ (M+H^+^) 608.24902, found 608.24725.

##### Synthesis of 2-((4-oxo-8-Propyl-2-(3,4,5-trimethoxyphenyl)-4*H*-chromen-7-yl)oxy)-N-(3,4,5-trimethoxyphenyl)acetamide (**17**)

**Method A:** To a solution of (*E*)-2-(3-hydroxy-2-propyl-4-(3-(3,4,5-trimethoxyphenyl)acryloyl)phenoxy)-*N*-(3,4,5-trimethoxyphenyl)acetamide (**13**, 100 mg, 1 Eq, 168 µmol) in DMSO (10 mL/mmol of chalcone), I_2_ (427 µg, 0.01 Eq, 1.68 µmol) was added. The mixture was heated at reflux for 24 h. Then, the mixture was poured into ice and a sodium thiosulfate solution (10%) was added. The obtained mixture was extracted with ethyl acetate, washed with water, and then dried over anhydrous sodium sulfate. The solvent was evaporated to dryness and the obtained mixture was purified via TLC (SiO_2_; chloroform:methanol; 95:5). A yellow solid (30% yield) corresponding to (2-((4-oxo-8-propyl-2-(3,4,5-trimethoxyphenyl)-4*H*-chromen-7-yl)oxy)-*N*-(3,4,5-trimethoxyphenyl)acetamide (**17**) was obtained.

**Method B:** 2-((4-oxo-8-propyl-2-(3,4,5-trimethoxyphenyl)-4*H*-chromen-7-yl)oxy)acetic acid (**18**, 100 mg, 1 Eq, 233 µmol) was dissolved in 5 mL of anhydrous dichloromethane and *N*-ethyl-*N*-isopropylpropan-2-amine (60.3 mg, 81.3 µL, 2 Eq, 467 µmol), and 3,4,5-trimethoxyaniline (51.3 mg, 1.2 Eq, 280 µmol) was added to the mixture. After 10 min, COMU (150 mg, 1.5 Eq, 350 µmol) was also added to the mixture. The reaction proceeded for 5 h at room temperature with stirring. After completion, the reaction mixture was poured onto ice. The solution was subsequently washed with 1M HCl solution and saturated NaHCO_3_ solution, and dried over anhydrous sodium sulfate. The solvent was evaporated to dryness and the obtained reaction mixture was purified via TLC (SiO_2_; chloroform:methanol; 95:5). A yellow solid (17.5% yield) corresponding to (2-((4-oxo-8-propyl-2-(3,4,5-trimethoxyphenyl)-4*H*-chromen-7-yl)oxy)-*N*-(3,4,5-trimethoxyphenyl)acetamide (**17**) was obtained.

**(2-((4-oxo-8-propyl-2-(3,4,5-trimethoxyphenyl)-4*H*-chromen-7-yl)oxy)-*N*-(3,4,5-trimethoxyphenyl)acetamide (17)**: 96.4% purity; **mp** 195–197 °C (chloroform); **^1^H NMR** (DMSO, 400.13 MHz) *δ*: 10.15 (s,1H, 3″-NH-), 7.90 (d, *J* = 9.1 Hz, 1H, H-5), 7.36 (s, 2H, H-2‴, -6‴), 7.00 (s, 2H, H-5″, -9″), 7.14 (d, *J* = 9.0 Hz, 1H, H-6), 7.10 (s, 1H, H-3), 4.94 (s, 2H, H-1″), 3.92 (s, 6H, 3‴-, 5‴-OCH_3_), 3.76 (s, 3H, 4‴-OCH_3_), 3.76 (s, 6H, 6″-, 8″-OCH_3_), 3.62 (s, 3H, 7″-OCH_3_), 3.07 (t, *J* = 7.3 Hz, 1H, H-1′), 1.71–1.67 (m, 2H, H-2′), 1.01 (t, *J* = 7.3 Hz, 3H, H-3′) ppm; ^**13**^**C NMR** (DMSO, 100.63 MHz) *δ*: 177.0 (C=O), 166.9 (C-2″), 162.9 (C-2), 160.1 (C-7), 154.6 (C-8a), 153.0 (C-3‴, -5‴), 152.8 (C-6″, -8″), 140.0 (C-4‴), 134.6 (C-7″), 133.8 (C-4″), 126.7 (C-1‴), 123.7 (C-5), 119.5 (C-8), 118.5 (C-4a), 110.4 (C-6), 106.1 (C-3), 103.8 (C-2‴, -6‴), 97.0 (C-5″, -9″), 67.9 (C-1″), 60.3 (4‴-OCH_3_), 60.1 (7″-OCH_3_), 56.1 (3‴-, 5‴-OCH_3_), 55.7 (6″-, 8″-OCH_3_), 24.1 (C-1′), 22.3 (C-2′), 14.5 (C-3′) ppm; **HRMS** (ESI^+^): m/z Anal. Calc. for C_33_H_35_NO_10_ (M+K^+^) 632.18925, found 632.18769.

##### 2-((4-oxo-8-Propyl-2-(3.4.5-trimethoxyphenyl)-4*H*-chromen-7-yl)oxy)acetic acid (**18**)

To a solution of (*E*)-2-(3-hydroxy-2-propyl-4-(3-(3,4,5-trimethoxyphenyl)acryloyl)phenoxy)acetic acid (**11**, 100 mg, 1 Eq, 232 µmol) in DMSO (10mL/mmol of chalcone), I_2_ (590 µg, 0.01 Eq, 2.32 µmol) was added. The mixture was heated at reflux for 24 h. Then, the mixture was poured into ice and a sodium thiosulfate solution (10%) was added. The obtained mixture was extracted with ethyl acetate, washed with water, and then dried over anhydrous sodium sulfate. The solvent was evaporated to dryness, and the obtained oil was purified via TLC (SiO_2_, n-hexane: ethyl acetate:formic acid (3:7:0.1%). A white solid (35% yield) corresponding to 2-((4-oxo-8-propyl-2-(3,4,5-trimethoxyphenyl)-4*H*-chromen-7-yl)oxy)acetic acid (**18**) was obtained.

**2-((4-oxo-8-propyl-2-(3.4.5-trimethoxyphenyl)-4*H*-chromen-7-yl)oxy)acetic acid** (**18**): 95.9% purity; **mp** 199–201 °C (chloroform); **^1^H NMR** (THF, 400.13 MHz) *δ*: 7.92 (d, *J* = 8.8 Hz, 1H, H-5), 7.38 (s, 2H, H-2‴, -6‴), 7.10 (d, *J* = 8.9 Hz, 1H, H-6), 7.01 (s, 1H, H-3), 4.85 (s, 2H, H-1″), 3.94 (s, 6H, 3‴-, 5‴-OCH_3_), 3.79 (s, 3H, 4‴-OCH_3_), 3.04 (t, *J* = 7.7 Hz, 2H, H-1′), 1.73–1.72 (m, 2H, H-2′), 1.04 (t, *J* = 7.4 Hz, 3H, H-3′) ppm; ^**13**^**C NMR** (THF, 100.63 MHz) *δ*: 178.1 (C=O), 170.8 (C-2″), 163.2 (C-2), 161.1 (C-7), 156.1 (C-8a), 154.9 (C-3‴, -5‴), 142.2 (C-4‴), 128.3 (C-1‴), 124.9 (C-5), 119.8 (C-8), 119.2 (C-4a), 111.1 (C-6), 107.3 (C-3), 104.7 (C-2‴, -6‴), 66.4 (C-1″), 61.0 (4‴-OCH_3_), 57.0 (3‴-, 5‴-OCH_3_), 26.4 (C-1′), 23.5 (C-2′), 15.1 (C-3′) ppm; **HRMS** (ESI^+^): m/z Anal. Calc. for C_23_H_24_O_8_ (M+H^+^) 429.15439, found 429.15658.

### 3.2. Biological Activity

#### 3.2.1. Cell Culture

A375-C5 (melanoma) and MCF-7 (breast adenocarcinoma) cell lines were obtained from the European Collection of Cell Culture, UK, and were grown in RPMI-1640 medium (Roswell Park Memorial Institute, Biochrom, Cambridge, UK) with 5% inactivated FBS (fetal bovine serum, Biochrom). HCT-116 (colon adenocarcinoma) cells were grown in DMEM medium (Dulbecco’s Modified Eagle’s, Biochrom) with 5% inactivated FBS. The non-tumor human pulmonary alveolar epithelial cells (HPAEpiC) were obtained from ScienCell Research Laboratories and grown in DMEM medium supplemented with 10% FBS and 1% non-essential amino acids (Sigma-Aldrich Co., Saint Louis, MO, USA). All cell lines were maintained in a humidified incubator (Hera Cell, Heraeus, Hanau, Germany) at 37 °C and with 5% CO_2_, and cell viability was regularly monitored using the exclusion trypan blue dye.

#### 3.2.2. Compounds

All tested compounds were dissolved to 60 µM in sterile DMSO (Sigma-Aldrich Co., Ltd., Gillingham, UK). To preserve the activities of compounds, several aliquots were prepared and stored at −20 °C. Immediately prior to use, compounds were diluted in fresh culture medium at the required concentrations.

#### 3.2.3. Sulforodamine B (SRB) Assay

A total of 5.0 × 10^4^ A375-C5, MCF-7, and HCT-116 cells, or 6.0 × 10^4^ HPAEpiC cells were seeded in 96-well plates and incubated at 37 °C and 5% CO_2_ to allow for cell attachment. After 24 h, cells were treated with two-fold serial dilutions of test compounds (0–150 µM), or with DMSO as the compound solvent. In parallel, cells in a time-zero (T0) plate, without treatment, were fixed with 50% (*m*/*v*) trichloroacetic acid (TCA, Merck Millipore, Darmstadt, Germany), for 1 h at 4 °C, and then washed with distilled water and left to dry. Forty-eight hours after compound treatment, cells were fixed with 50% TCA and stained with SRB (Sigma-Aldrich Co., Ltd., Gillingham, UK) for 30 min at room temperature, followed by 5 rounds of washing with 1% (*v*/*v*) acetic acid (Merck Milli-pore, Darmstadt, Germany). SRB–protein complexes were then solubilized with 10 µM Tris buffer (Sigma-Aldrich Co., Ltd., Gillingham, UK) for 30 min, and the absorbance was measured at 515 nm in a microplate reader (Biotek Synergy 2, BioTek Instruments, Inc., Winooski, VT, USA). A dose-response curve was obtained for each cell line, and the concentration that caused 50% cell growth inhibition (GI_50_) was calculated.

#### 3.2.4. Yeast Targeted Screening Assay

*Saccharomyces cerevisiae* (strain CG379) expressing human *wt* p53 alone and combined with human MDM2 were obtained in previous works [32,33]. For the expression of human proteins (routinely grown in minimal selective medium), cells were diluted to 0.05 OD600 in selective induction medium containing 2% (*w*/*w*) galactose, 1% (*w*/*w*) raffinose, 0.7% (*w*/*w*) yeast nitrogen base without amino acids from Difco (Quilaban, Sintra, Portugal), and all the amino acids required for yeast growth (50 g/mL) except leucine and tryptophan. Yeast cells were incubated at 30 °C under continuous orbital shaking (200 rpm) with 10 µM compounds or 0.1% DMSO only, for approximately 42 h (time required by the control yeast, co-transformed with the empty vectors and incubated with DMSO only, to achieve 0.4 OD600). Yeast growth was analyzed by counting the number of colony-forming units (CFU) after 2 days of incubation at 30 °C on Sabouraud Dextrose Agar from Liofilchem (Frilabo, Porto, Portugal).

#### 3.2.5. Apoptosis Detection via Flow Cytometry

To evaluate apoptotic cell death, the “Annexin V-FITC Apoptosis Detection Kit” (eBioscience, Vienna, Austria) was used according to the manufacturer’s instructions. Briefly, 1.2 × 10^5^ cells/well were seeded into 6-well plates, and 24 h later, cells were treated with compounds **6**, **7**, and **13**. After 48 h, floating and adherent cells were collected, pelleted via centrifugation (1000 rpm, 5 min), and suspended in binding buffer. Annexin V-FITC was added and incubated for 10 min, protected from light. Cells were washed and resuspended in binding buffer, and 20 μg/mL of propidium iodide (PI) was added. Fluorescence was assessed using the BD Accuri™ C6 Plus flow cytometer (BD Biosciences, Qume Drive, San Jose, CA, USA), and the data were analyzed with the BD Accuri TM C6 Plus software (version 1.0.27.1, San Jose, CA, USA). At least 20,000 events per sample were collected.

#### 3.2.6. DAPI Staining

A total of 1.2 × 10^5^ cells were grown on poly-L-lysine-coated coverslips for 24 h. Then, cells were treated with **6**, **7**, and **13**, and fixed with methanol (Sigma-Aldrich, Co., Ltd., Gillingham, UK) for 10 min at −20 °C and washed 3 times with PBS for 5 min. DNA was stained with 2 µg/mL 40,6-diamidino-2-phenylindole (DAPI, Sigma-Aldrich) diluted in Vectashield mounting medium (Vector, H-1000, Burlingame, CA, USA).

#### 3.2.7. Mitotic Index Determination

A total of 1.2 × 10^5^ HCT-116 cells were grown in six-well dishes and treated for 24 h with 3.26 µM of compound **7**. Cells treated with 1 µM nocodazole were used as a positive control for antimitotic activity. Untreated cells and cells treated with DMSO were included to exclude the cytotoxicity of the compound solvent. Mitotic index, and the percentage of mitotic cells over the total cell population were determined via cell-rounding under phase-contrast microscopy from at least ten random microscope fields.

#### 3.2.8. Immunofluorescence

Cells were treated as for DAPI staining. After methanol fixation, cells were blocked with 10% FBS in PBST (0.05% Tween-20 in PBS) for 30 min at room temperature, followed by 1 h incubation with a primary antibody (mouse anti-α-tubulin, 1:2500, Sigma-Aldrich Co., Ltd., Gillingham, UK) diluted in 5% FBS in PBST. Then, cells were washed three times in PBST and incubated with an Alexa Fluor 488 conjugated secondary antibody (1:1500, Molecular Probes, Eugene, OR, USA). DNA was stained with DAPI.

#### 3.2.9. Image Acquisition and Processing

For phase contrast microscopy images, a Nikon TE 2000-U microscope (Nikon, Amsterdam, The Netherlands) with a 10× objective was used, coupled to a DXM1200F digital camera with Nikon ACT-1 software (Melville, NY, USA). For fluorescence images, an Axio Observer Z.1 SD microscope (Carl Zeiss, Germany) was used, coupled to an AxioCam MR3, with the Plan Apochromatic 63×/NA 1.4 objective. The images were processed using ImageJ, version 1.51.

#### 3.2.10. Statistical Analysis

All assays were performed in triplicate with at least three independent experiments. Data were expressed as mean ± standard deviation (SD), statistical analysis was carried out in GraphPad Prism Software Inc. v6 using an unpaired t-test, and values of * *p* < 0.05, ** *p* < 0.01, *** *p* < 0.001, and **** *p* < 0.0001 were considered as statistically significant.

### 3.3. In Silico Prediction of Drug-Likeness

The selected compounds were further investigated for drug-likeness properties, namely, molecular descriptors/physicochemical properties, by SwissADME (http://www.swissadme.ch/, (accessed on 12 May 2023).

## 4. Conclusions

Inspired by **CM-M345** (**1**) and **BP-M345** (**2**), a new series of chalcone-trimethoxycinnamide/trimethoxyphenylamide hybrids have been designed, synthesized, and evaluated for their antitumor activity against melanoma (A375-C5), breast adenocarcinoma (MCF-7), and colorectal carcinoma (HCT116) cell lines, as well as non-tumor HPAEpiC cells. Among these, compounds **6**, **7**, and **13** demonstrated potent antiproliferative activity in the studied human tumor cell lines, with this effect being particularly intensified against colorectal tumor cells, with a relevant degree of selectivity when compared to non-tumor cells for compound **7**. The three referred compounds exert antitumor activity against colorectal tumor cells through apoptosis. Mechanistically, compound **7** emerged as an antimitotic agent that, through its antimicrotubule activity, induces mitotic delay in colorectal tumor cells, and consequently, cell death. Notably, compound **7** affected colorectal tumor cells more severely than the non-tumor cells, indicating a high degree of selectivity, as suggested by the 17-fold higher GI_50_ value of the compound on non-tumor cells. This highlights the potential of **7** as a hit compound, since the discovery of compounds with selective cytotoxicities that are capable of distinguishing between normal and tumor cells is a major challenge in tumor therapy. Considering the results obtained through in silico drug-likeness prediction, in the near future, the optimization of this compound should also consider the improvement of its drug-likeness profile, namely through nanotechnology.

## Figures and Tables

**Table 1:** Tumor cell growth inhibitory activities of compounds **1**, **2**, **6–8**, and **11–18**.

	GI_50_ (μM) ^1^			
	A375-C5	MCF-7	HCT116	HPAEpiC
**6**	3.61 ± 1.11	39.81 ± 2.11	2.81 ± 0.65	9.60 ± 0.94
**7**	32.07 ± 1.27	>75	3.26 ± 1.39	57.17 ± 8.68
**8**	>75	>75	13.49 ± 2.46	>75
**11**	29.36 ± 1.05	58.34 ± 8.37	26.87 ± 1.60	>75
**12**	>75	>75	>75	>75
**13**	9.37 ± 0.90	18.80 ± 3.46	2.66 ± 0.87	10.83 ± 0.23
**14**	>75	>75	>75	>75
**15**	>75	>75	>75	>75
**16**	>75	>75	28.57 ± 5.05	>75
**17**	>75	>75	24.68 ± 3.04	>75
**18**	>75	>75	>75	>75
**CM-M345** (**1**)	5.57 ± 0.06	2.3 ± 0.1 ^2^	2.6 ± 0.2 ^2^	6.10 ± 0.66
**BP-M345** (**2**)	0.24 ± 0.01 ^3^	0.45 ± 0.06 ^3^	0.17 ± 0.01 ^4^	1.07± 0.16 ^3^
Doxorubicin	0.028 ± 0.002	0.083 ± 0.003	0.085 ± 0.008	0.091 ± 0.013

^1^ GI_50_ represents the concentration that causes 50% cell growth inhibition at 48 h. Doxorubicin was used as positive control. The results are expressed as mean ± standard deviation from three independent experiments; ^2^ Hit compound previously reported by Pereira et al. [16]; **^3^** Hit compound previously reported by Novais et al. [17]; ^4^ Results previously reported by Moreira et al. [12].

**Table 2:** Tumor cell growth inhibitory activities of compounds **6**, **7**, and **13** in human tumor HCT116 p53^+/+^ and HCT116 p53^−/−^ cells.

GI_50_ (μM) ^1^			
	**HCT116 p53^+/+^**	**HCT116 p53^−/−^**	SI ^2^
**6**	2.81 ± 0.65	1.9 ± 0.3	0.68
**7**	3.26 ± 1.39	1.3 ± 0.09	0.40
**13**	2.66 ± 0.87	4.1 ± 0.5	1.54
**CM-M345** (**1**) ^3^	2.6 ± 0.2 ^2^	3.4 ± 0.1 ^2^	1.31

^1^ GI_50_ represents the concentration that causes 50% cell growth inhibition at 48 h. ^2^ Selective index (GI_50_ of HCT116 p53^−/−^/GI_50_ of HCT116 p53^+/+^). Etoposide was used as positive control: GI_50_ (HCT116 p53^+/+^) = 0.71 ± 0.09 µM. ^3^ Hit compound previously reported by Pereira et al. [16]. The results are expressed as mean ± standard deviation from 3–5 independent experiments.

## Data Availability

Data is contained within the article.

## Supplementary Materials

The following are available online https://www.mdpi.com/article/10.3390/ph16060879/s1. **Figure S1**. ^1^H and ^13^C NMR of compound **3**, **Figure S2**. ^1^H and ^13^C NMR of compound **4**, **Figure S3**. ^1^H and ^13^C NMR of compound **5**, **Figure S4**. ^1^H and ^13^C NMR of compound **6**, **Figure S5**. ^1^H and ^13^C NMR of compound **7**, **Figure S6**. ^1^H and ^13^C NMR of compound **8**, **Figure S7**. ^1^H and ^13^C NMR of compound **9**, **Figure S8**. ^1^H and ^13^C NMR of compound **10**, **Figure S9**. ^1^H and ^13^C NMR of compound **11**, **Figure S10**. ^1^H and ^13^C NMR of compound **12**, **Figure S11**. ^1^H and ^13^C NMR of compound **13**, **Figure S12**. ^1^H and ^13^C NMR of compound **14**, **Figure S13**. ^1^H and ^13^C NMR of compound **15**, **Figure S14**. ^1^H and ^13^C NMR of compound **16**, **Figure S15**. ^1^H and ^13^C NMR of compound **17**, **Figure S16**. ^1^H and ^13^C NMR of compound **18**, **Figure S17**. ^1^H and ^13^C NMR of compound **19**, **Figure S18**. ^1^H and ^13^C NMR of compound **20**, **Figure S17**. HRMS of compound **5**, **Figure S18**. HRMS of compound **6**, **Figure S19**. HRMS of compound **7**, **Figure S20**. HRMS of compound **8**, **Figure S21**. HRMS of compound **10**, **Figure S22**. HRMS of compound **11**, **Figure S23**. HRMS of compound **12**, **Figure S24**. HRMS of compound **13**, **Figure S25**. HRMS of compound **14**, **Figure S26**. HRMS of compound **15**, **Figure S27**. HRMS of compound **16**, **Figure S28**. HRMS of compound **17**, **Figure S29**. HRMS of compound **18**, **Figure S30**. Peak purity of compound **5**, **Figure S31**. Peak purity of compound **6**, **Figure S32**. Peak purity of compound **7**, **Figure S33**. Peak purity of compound **8**, **Figure S34**. Peak purity of compound **9**, **Figure S35**. Peak purity of compound **10**, **Figure S36**. Peak purity of compound **11**, **Figure S37**. Peak purity of compound **12**, **Figure S38**. Peak purity of compound **13**, **Figure S39**. Peak purity of compound **14**, **Figure S40**. Peak purity of compound **15**, **Figure S41**. Peak purity of compound **16**, **Figure S42**. Peak purity of compound **17**, **Figure S43**. Peak purity of compound **18**.
