# Supplementary material for: Discovery of a New Chalcone-Trimethoxycinnamide Hybrid with Antimitotic Effect: Design, Synthesis, and Structure—Activity Relationship Studies
Source: Pharmaceuticals (Basel). 2023 Jun 14;16(6):879. doi: 10.3390/ph16060879 (PMC10303900; doi:10.3390/ph16060879)
Supplement: Supplementary file 1 [file pharmaceuticals-16-00879-s001.zip › pharmaceuticals-2388054-supplementary.pdf]

Supplementary Materials

Discovery of a New Chalcone-Trimethoxycinnamide Hybrid with Antimitotic Effect: Design, Synthesis and Structure-Activity Relationship Studies

Joana Moreira [1,2,†], Patrícia M. A. Silva [3,4,†], Matilde Barros [5], Lucília Saraiva [5], Madalena Pinto [1,2], Hassan Bousbaa [3,*] and Honorina Cidade [1,2,*]

1 Laboratory of Organic and Pharmaceutical Chemistry, Department of Chemical Sciences, Faculty of Pharmacy, University of Porto, Rua de Jorge Viterbo Ferreira 228, 4050-313 Porto, Portugal; up201302558@edu.ff.up.pt (J.M.); madalena@ff.up.pt (M.P.).
2 Interdisciplinary Centre of Marine and Environmental Research (CIIMAR), University of Porto, Edifício do Terminal de Cruzeiros do Porto de Leixões, Avenida General Norton de Matos, S/N, 4450-208 Matosinhos, Portugal.
3 UNIPRO—Oral Pathology and Rehabilitation Research Unit, University Institute of Health Sciences (IUCS), CESPU, Rua Central de Gandra, 1317, 4585-116 Gandra, Portugal; patricia.silva@cespu.pt (P.M.A.S.)
4 TOXRUN – Toxicology Research Unit, University Institute of Health Sciences, CESPU, CRL, 4585-116 Gandra, Portugal.
5 LAQV/REQUIMTE, Laboratory of Microbiology, Department of Biological Sciences, Faculty of Pharmacy, University of Porto, Rua Jorge Viterbo Ferreira, 228, 4050-313 Porto, Portugal; up201604459@edu.ff.up.pt (M.B.); lucilia.saraiva@ff.up.pt (L.S.)
† Authors contributed equally to this work
* Correspondence: hcidade@ff.up.pt (H.C.); Tel.: +351-220428688 (H.C.); hassan.bousbaa@iucs.cespu.pt (H.B.) Tel.: +351- 224157186 (H.B.)

Table of Contents

List of Figures 2

Experimental description of compounds 3, 4 and 9 4

Synthesis of Methyl (*E*)-3-(3,4,5-trimethoxyphenyl) acrylate (3) 4

Synthesis of (*E*)-3-(3,4,5-trimethoxyphenyl) acrylic acid (4) 5

Synthesis of methyl 2-(4-acetyl-3-hydroxy-2-propylphenoxy)acetate (9) 5

NMR spectra 7

HRMS spectra 23

HPLC analysis 36

List of Figures

Figure S1. ^{1}H and ^{13}C NMR spectra of compound **3**. 7

Figure S2. ^{1}H and ^{13}C NMR spectra of compound **4**. 8

Figure S3. ^{1}H and ^{13}C NMR spectra of compound **5**. 9

Figure S4. ^{1}H and ^{13}C NMR spectra of compound **6**. 10

Figure S5. ^{1}H and ^{13}C NMR spectra of compound **7**. 11

Figure S6. ^{1}H and ^{13}C NMR spectra of compound **8**. 12

Figure S7. ^{1}H and ^{13}C NMR spectra of compound **9**. 13

Figure S8. ^{1}H and ^{13}C NMR spectra of compound **10**. 14

Figure S9. ^{1}H and ^{13}C NMR spectra of compound **11**. 15

Figure S10. ^{1}H and ^{13}C NMR spectra of compound **12**. 16

Figure S11. ^{1}H and ^{13}C NMR spectra of compound **13**. 17

Figure S12. ^{1}H and ^{13}C NMR spectra of compound **14**. 18

Figure S13. ^{1}H and ^{13}C NMR spectra of compound **15**. 19

Figure S14. ^{1}H and ^{13}C NMR spectra of compound **16.** 20

Figure S15. ^{1}H and ^{13}C NMR of spectra compound **17**. 21
Figure S16. ^{1}H and ^{13}C NMR spectra of compound **18**. 22
Figure S17. HRMS of compound **5**. 23
Figure S18. HRMS of compound **6**. 24
Figure S19. HRMS of compound **7**. 25
Figure S20. HRMS of compound **8**. 26
Figure S21. HRMS of compound **10**. 27
Figure S22. HRMS of compound **11**. 28
Figure S23. HRMS of compound **12**. 29
Figure S24. HRMS of compound **13**. 30
Figure S25. HRMS of compound **14**. 31
Figure S26. HRMS of compound **15**. 32
Figure S27. HRMS of compound **16.** 33
Figure S28. HRMS of compound **17**. 34
Figure S29. HRMS of compound **18**. 35
Figure S30. Peak purity of compound **5**. 36
Figure S31. Peak purity of compound **6**. 37
Figure S32. Peak purity of compound **7**. 38
Figure S33. Peak purity of compound **8**. 39
Figure S34. Peak purity of compound **9**. 40
Figure S35. Peak purity of compound **10**. 41
Figure S36. Peak purity of compound **11**. 42
Figure S37. Peak purity of compound **12**. 43
Figure S38. Peak purity of compound **13**. 44
Figure S39. Peak purity of compound **14**. 45
Figure S40. Peak purity of compound **15**. 46
Figure S41. Peak purity of compound **16**. 47
Figure S42. Peak purity of compound **17**. 48
Figure S43. Peak purity of compound **18**. 49

In silico prediction of drug-likeness

Table S1. Drug-likeness prediction of compounds **6**, **7**, and **13** (molecular descriptors and physicochemical properties).

Molecular descriptor	6	7	13
**Formula**	$C_{28}H_{37}NO_8$	$C_{35}H_{41}NO_{10}$	$C_{32}H_{37}NO_{10}$
**MW**	515.60	635.70	595.64
**Nº HA**	37	46	43
**Nº AHA**	12	18	18
**Far**	0.32	0.39	0.42
**Fsp^3**	0.43	0.31	0.31
**RB**	15	18	16
**Nº HBA**	8	10	10
**Nº HBD**	2	2	2
**TPSA** [(a)]	112.55	131.01	131.01
**Log P (iLOGP)**[(a)]	4.83	5.25	4.64
**Log P (XLOGP3)**[(a)]	5.81	6.42	5.93
**Log P (WLOGP)**[(a)]	5.06	5.28	5.01
**Log P (MLOGP)**[(a)]	2.12	1.61	1.40
**Log P (SILICOS-IT)**[(a)]	5.57	7.17	6.08
**Log P (Consensus)**[(a)]	4.68	5.15	4.61
**Mean of Log P values**	4.68	5.15	4.61
**Log S (ESOL)**[(a)]	-5.95	-6.93	-6.52
**Log S (Ali)**[(a)]	-7.94	-8.96	-8.46
**Log S (SILICOS-IT)**[(a)]	-7.26	-9.15	-8.71
**Mean of Log S values**	-7.05	-8.35	-7.90

Experimental description of compounds 3, 4 and 9

*Synthesis of Methyl (E)-3-(3,4,5-trimethoxyphenyl) acrylate (**3**)*

To a solution of sinapic acid (2 g, 1 Eq, 9 mmol) in anhydrous acetone was added K_2CO_3 (4 g, 3.5 Eq, 0.03 mol). Dimethyl sulfate (3 g, 3 mL, 3 Eq, 0.03 mol) was added dropwise and the mixture was allowed to stir (24 h) at reflux and under nitrogen atmosphere. After completion, the reaction mixture was poured into ice. The solution was subsequently washed with saturated $NaHCO_3$ and 1M HCl solution. The organic phase was dried with anhydrous sodium sulfate, filtered and solvent was evaporated under reduced pressure. The reaction mixture obtained was purified by crystallization with ethyl acetate. A white solid (95% yield) corresponding to Methyl (*E*)-3-(3,4,5-trimethoxyphenyl) acrylate (**3**) was obtained.

Methyl (*E*)-3-(3,4,5-trimethoxyphenyl) acrylate (**3**): **^{1}H NMR** ($CDCl_3$, 300 MHz): δ 7.61 (d, *J* = 15.9 Hz, 1H, H-3''), 6.75 (s, 2H, H-5'', -9''), 6.35 (d, *J* = 15.9 Hz, 1H, H-2''), 3.88 (s, 6H, H-6'', -8''), 3.87 (s, 3H, H-7''), 3.80 (s,

3H, H-1′′) ppm; **^{13}C NMR** ($CDCl_3$, 75.47 MHz): δ 167.5 (C-1′′), 153.5 (C-6′′, -8′′), 145.0 (C-3′′), 140.2 (C-7′′), 130.0 (C-4′′), 117.1 (C-2′′), 105.3 (C-5′′, -9′′), 61.1 (7′′-OCH_3), 56.3 (6′′-, 8′′-OCH_3), 51.9 (1′′-OCH_3) ppm.

*Synthesis of (E)-3-(3,4,5-trimethoxyphenyl) acrylic acid (**4**)*

Methyl (E)-3-(3,4,5-trimethoxyphenyl)acrylate (2 g, 1 Eq, 8 mmol) was dissolved in THF. Then, 100 mL of H_2O and 100 mL of 37% HCl were added into the mixture. The reaction was allowed to proceed for 24 hours at 70 °C with gentle stirring. After completion, the reaction mixture was poured into ice, and saturated $NaHCO_3$ solution was added. The solution was then extracted with chloroform (3 x 50 mL). The organic phase was collected and further rinsed with water, dried over with anhydrous sodium sulfate and concentrated under reduced pressure. The reaction mixture was purified by flash column chromatography (SiO_2, chloroform : acetone; 95:5). A white solid (80% yield) corresponding to (*E*)-3-(3,4,5-trimethoxyphenyl)acrylic acid (**4**) was obtained.

(*E*)-3-(3,4,5-trimethoxyphenyl) acrylic acid (**4**): **^{1}H NMR** ($CDCl_3$, 300 MHz): δ 7.71 (d, *J* = 15.9 Hz, 1H, H-3′′), 6.78 (s, 2H, H-5′′, -9′′), 6.36 (d, *J* = 15.9 Hz, 1H, H-2′′), 3.90 (s, 6H, H-6′′, -8′′), 3.89 (s, 3H, H-7′′) ppm; **^{13}C NMR** ($CDCl_3$, 75.47 MHz): δ 172.4 (C-1′′), 153.6 (C-6′′, -8′′), 147.2 (C-3′′), 140.6 (C-7′′), 129.6 (C-4′′), 116.6 (C-2′′), 105.6 (C-5′′, -9′′), 61.1 (7′′-OCH_3), 56.3 (6′′-OCH_3, 8′′-OCH_3) ppm.

*Synthesis of methyl 2-(4-acetyl-3-hydroxy-2-propylphenoxy)acetate (**9**)*

1-(2,4-dihydroxy-3-propylphenyl)ethan-1-one (1 g, 1 Eq, 5.149 mmol) was dissolved in 5 mL of anhydrous acetone. Then, K_2CO_3 (711.5 mg, 1 Eq, 5.149 mmol) and methyl 2-bromoacetate (945.1 mg, 584.9 µL, 1.2 Eq, 6.178 mmol) were added. The reaction proceeded for 24 hours at 70 °C with gentle stirring. After cooling, the reaction mixture was poured into ice and neutralized with HCl. The obtained solid was filtrated, washed with water, and dried. A white solid (45% yield) corresponding to Methyl 2-(4-acetyl-3-hydroxy-2-propylphenoxy)acetate (**9**) was obtained.

Methyl 2-(4-acetyl-3-hydroxy-2-propylphenoxy)acetate (**99**): 99.7% purity; **mp** 153-154 ^{0}C (acetone); **^{1}H NMR** ($CDCl_3$, 300.13 MHz) *δ*: 12.74 (s, 1H, 2-OH), 7.57 (d, *J* = 9.0 Hz, 1H, H-6), 6.28 (d, *J* = 9.0 Hz, 1H, H-5), 4.72 (s, 2H, H-1′′), 3.80 (s, 3H, H-3′′), 2.69 (t, *J* = 7.6 Hz, 1H, H-1′), 2.56 (s, 3H, 1-COC$\underline{H_3}$), 1.64-1.53 (m, 2H, H-2′), 0.96 (t, *J* = 7.4 Hz, 3H, H-3′′) ppm; **^{13}C NMR** ($CDCl_3$, 75.47 MHz) *δ*: 203.2 (1-$\underline{C}OCH_3$), 169.0 (C-2′′),

162.5 (C-2), 161.6 (C-4), 130.0 (C-6), 119.3 (C-3), 115.0 (C-1), 102.4 (C-5), 65.2 (C-1′′), 52.5 (C-3′′), 26.5 (1-COC$\underline{H}_3$), 24.6 (C-1′), 22.0 (C-2′), 14.3 (C-3′) ppm.

NMR spectra

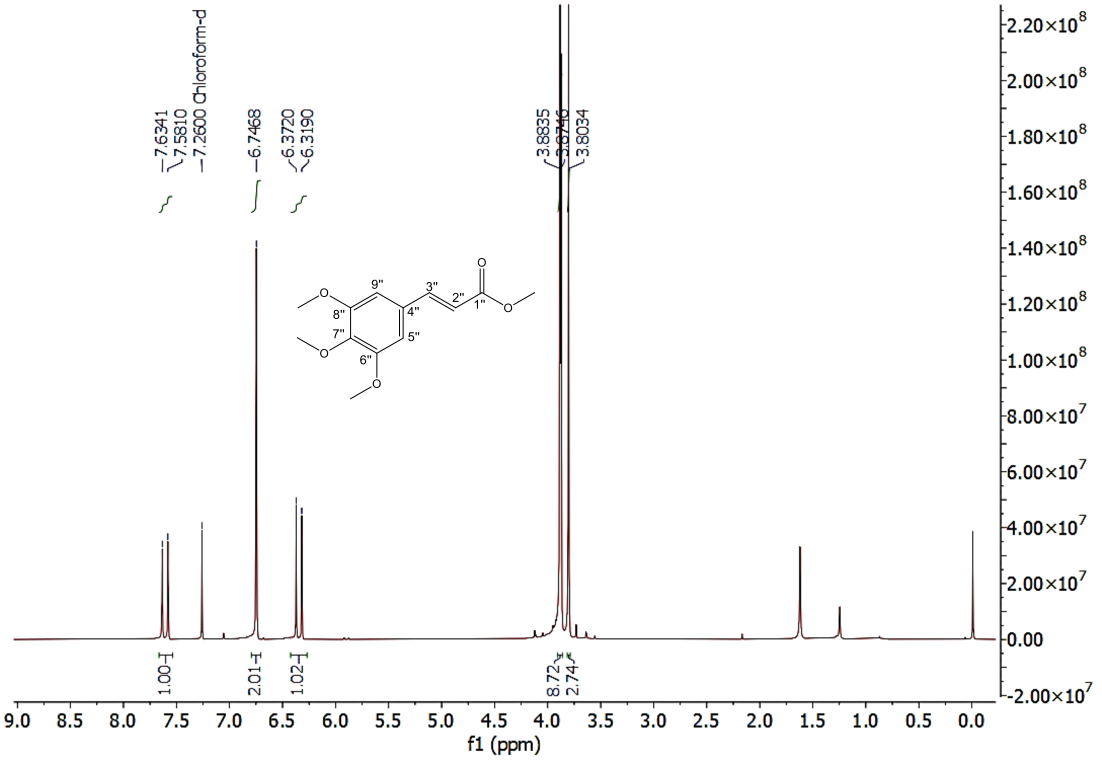

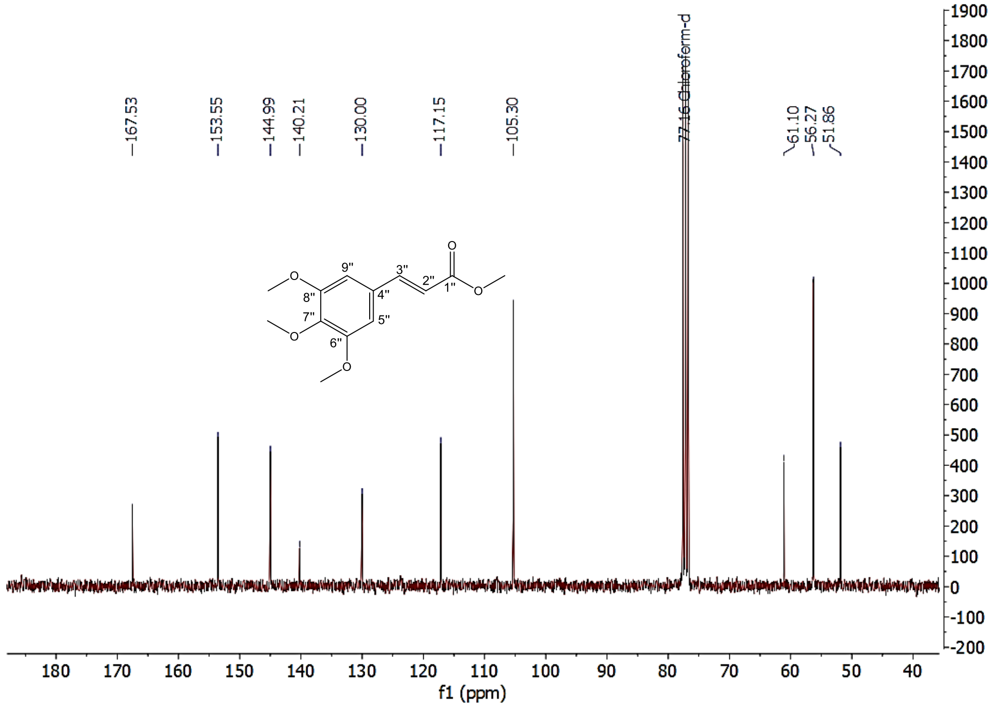

Figure S1. ^{1}H and ^{13}C NMR spectra of compound **3**.

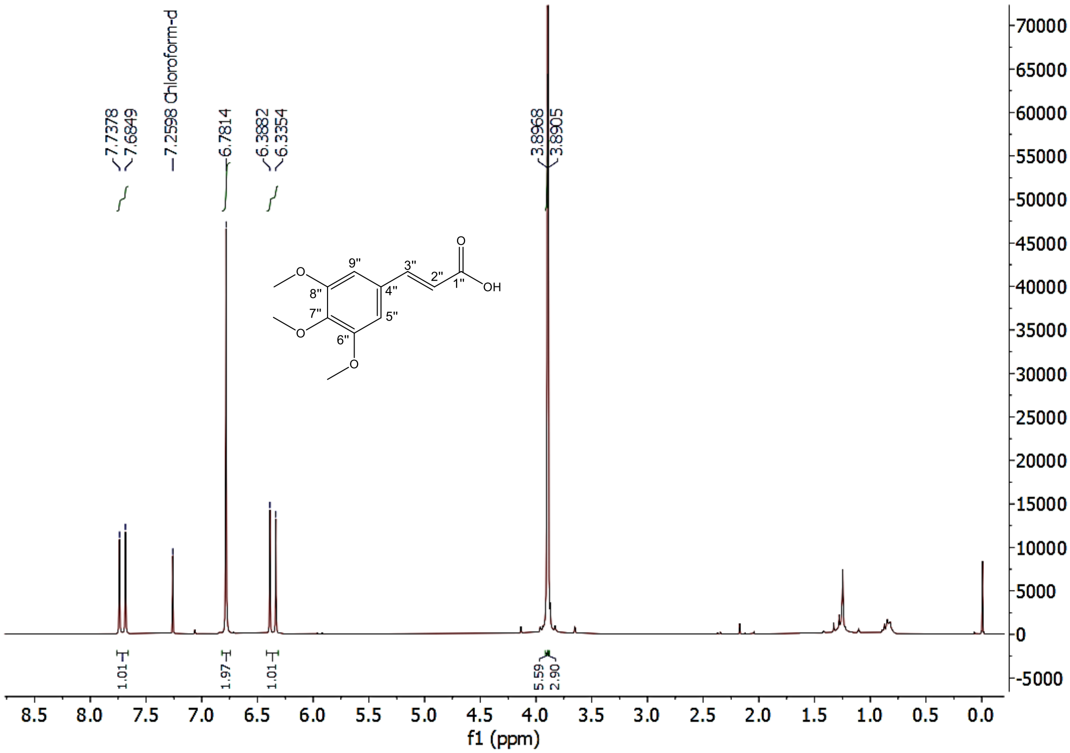

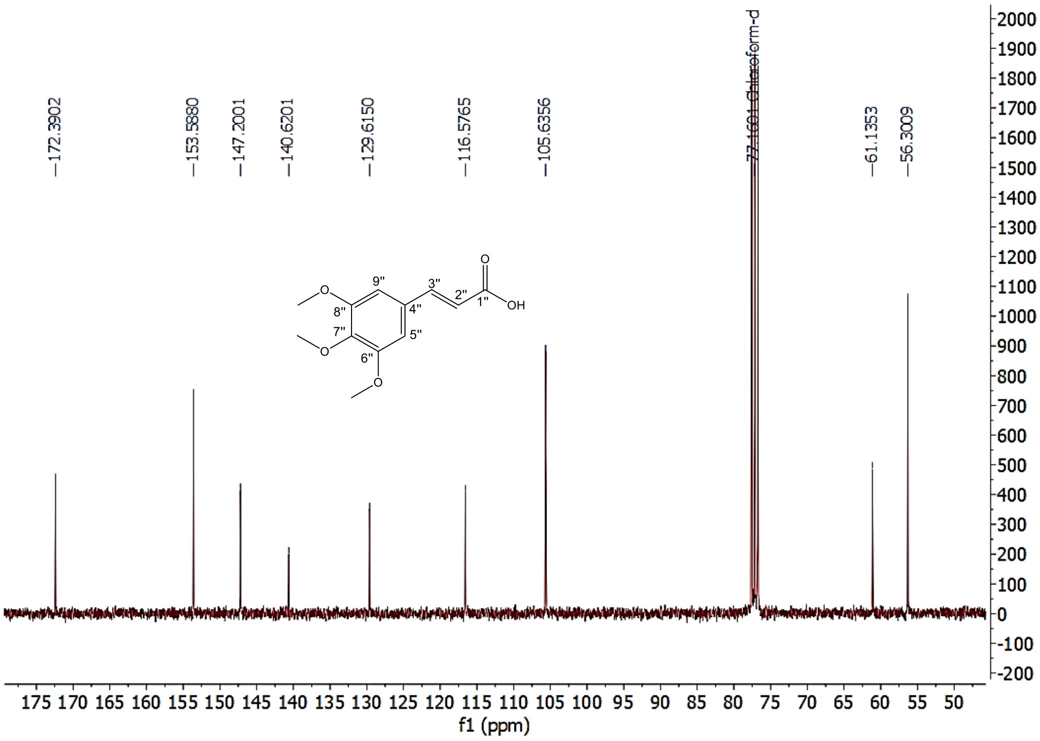

Figure S2. ^{1}H and ^{13}C NMR spectra of compound **4**.

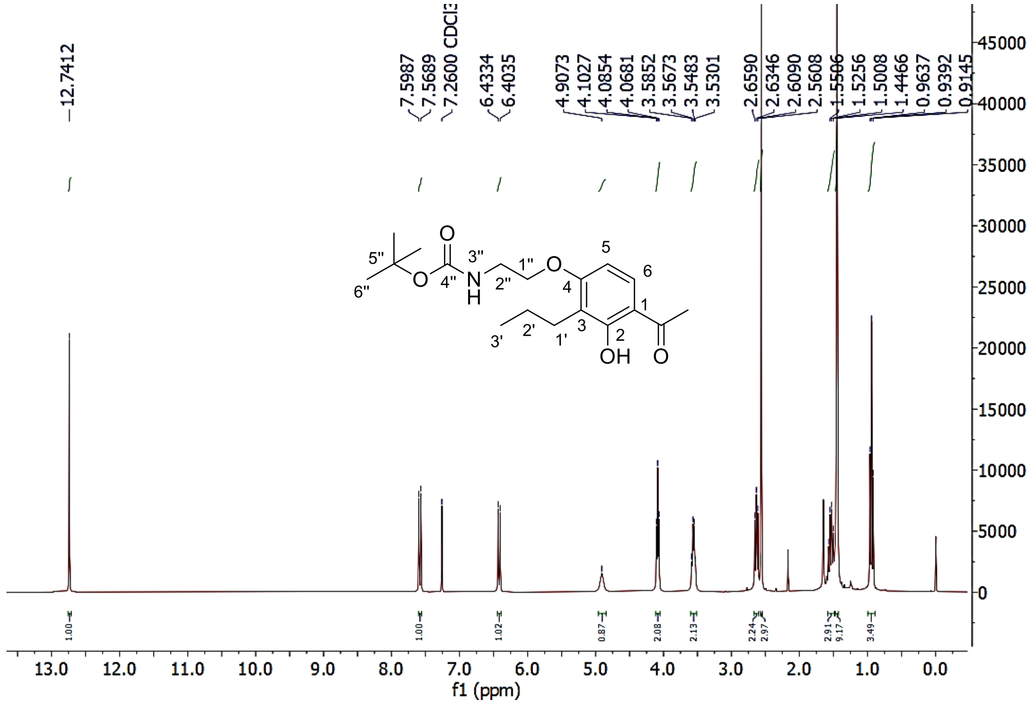

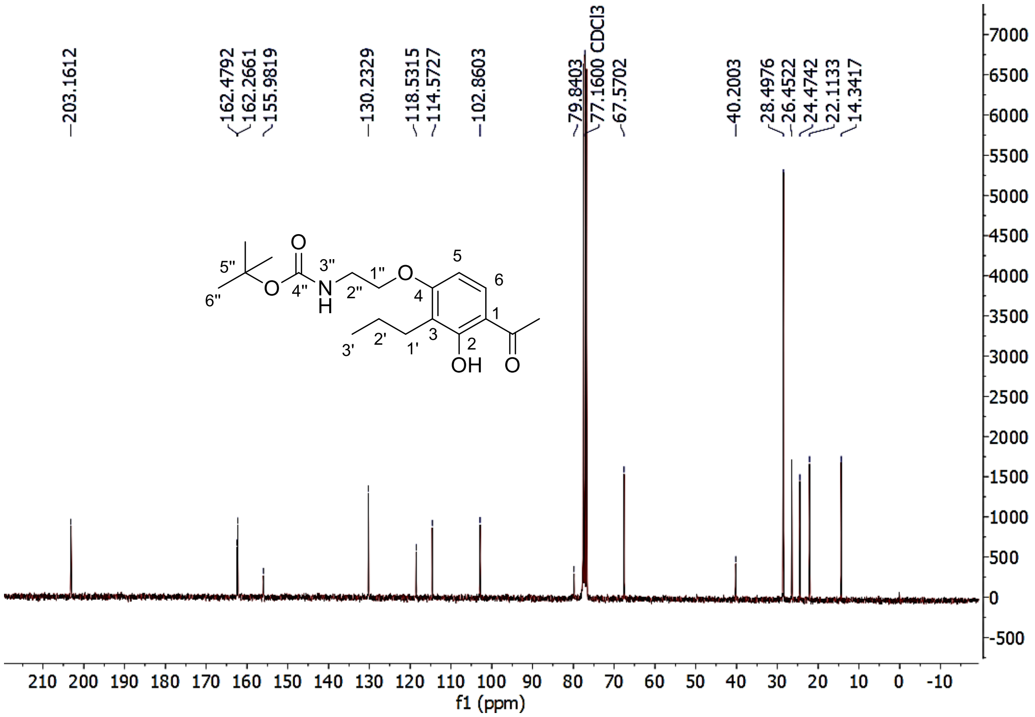

Figure S3. ^{1}H and ^{13}C NMR spectra of compound **5**.

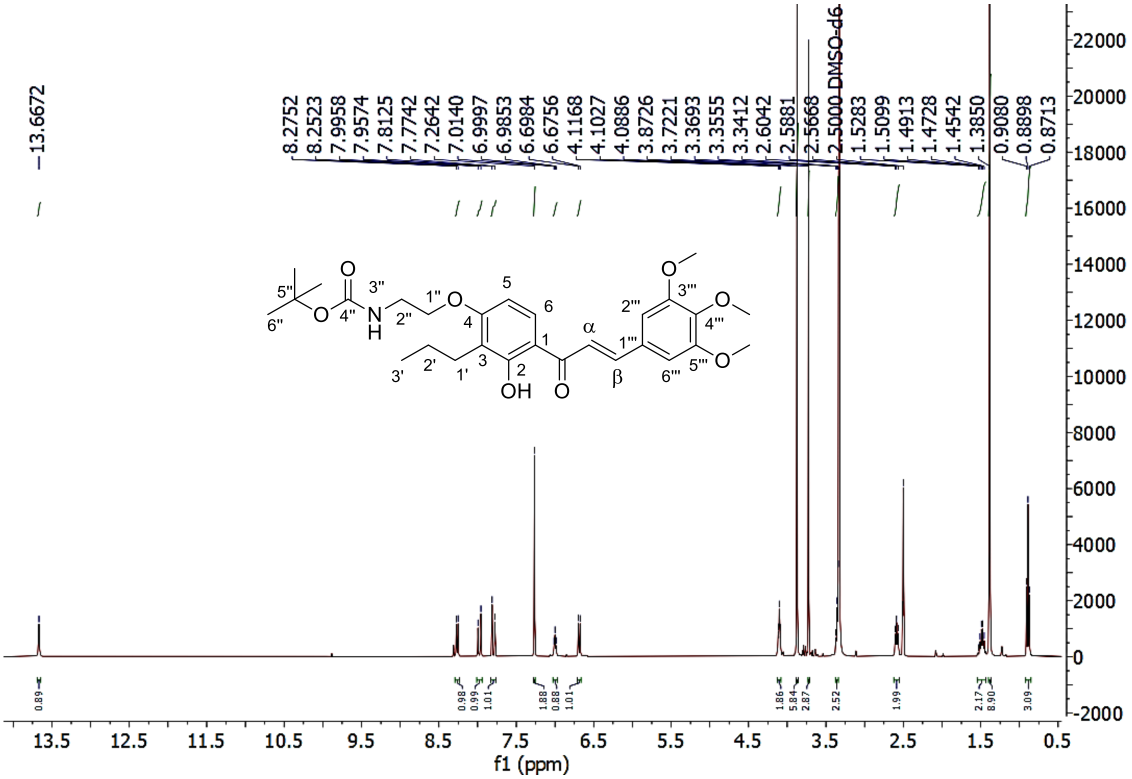

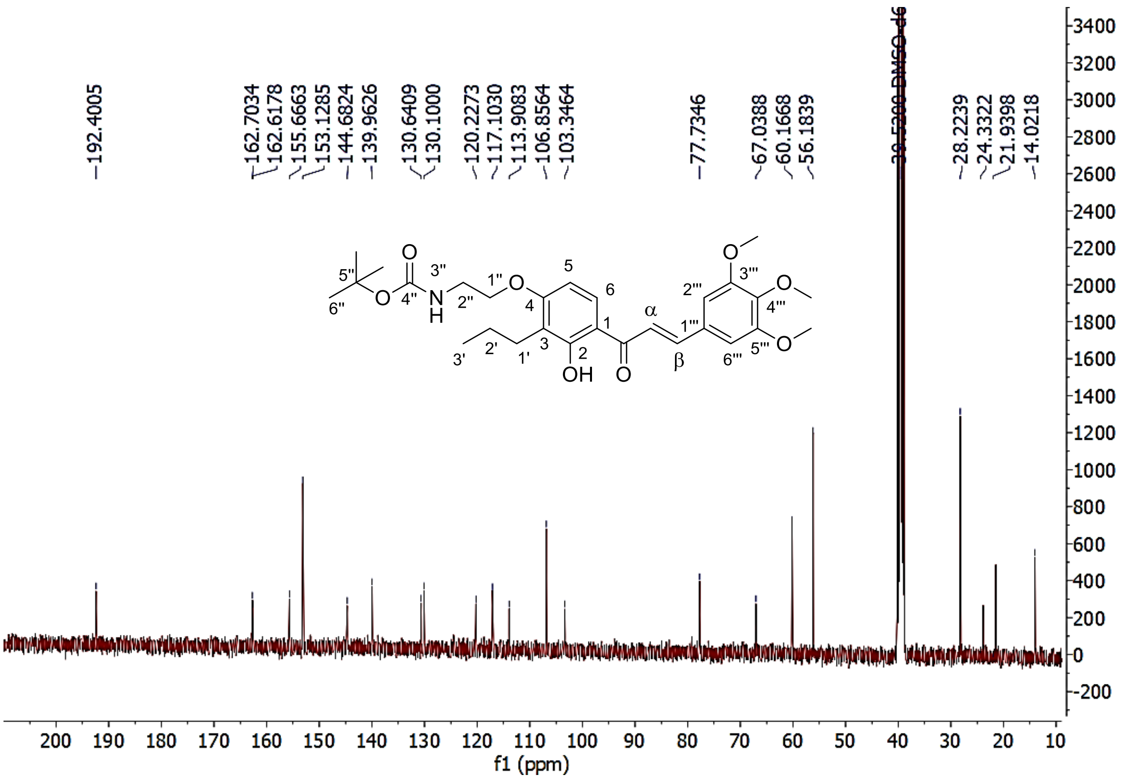

Figure S4. ^{1}H and ^{13}C NMR spectra of compound **6**.

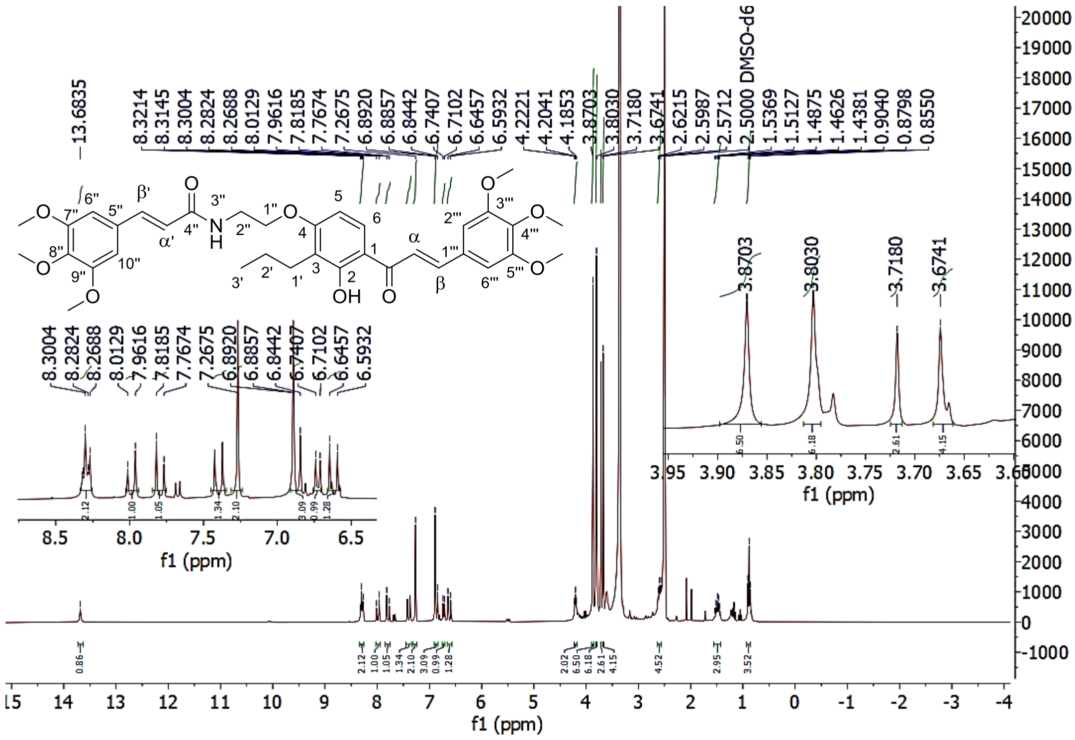

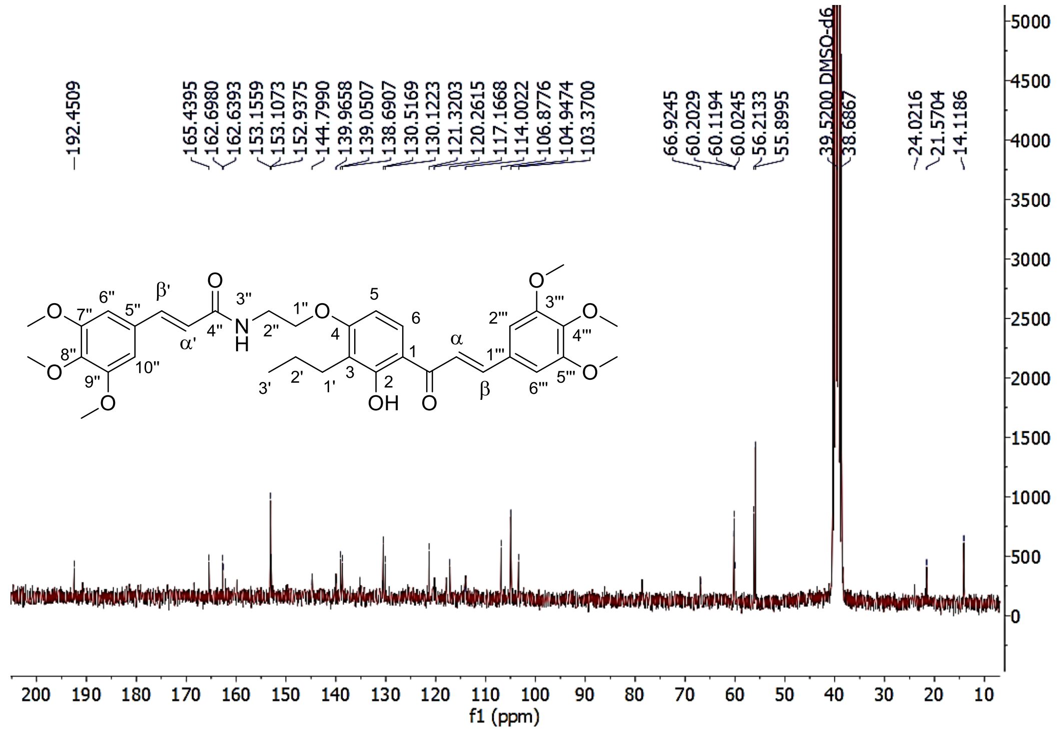

Figure S5. ^{1}H and ^{13}C NMR spectra of compound **7**.

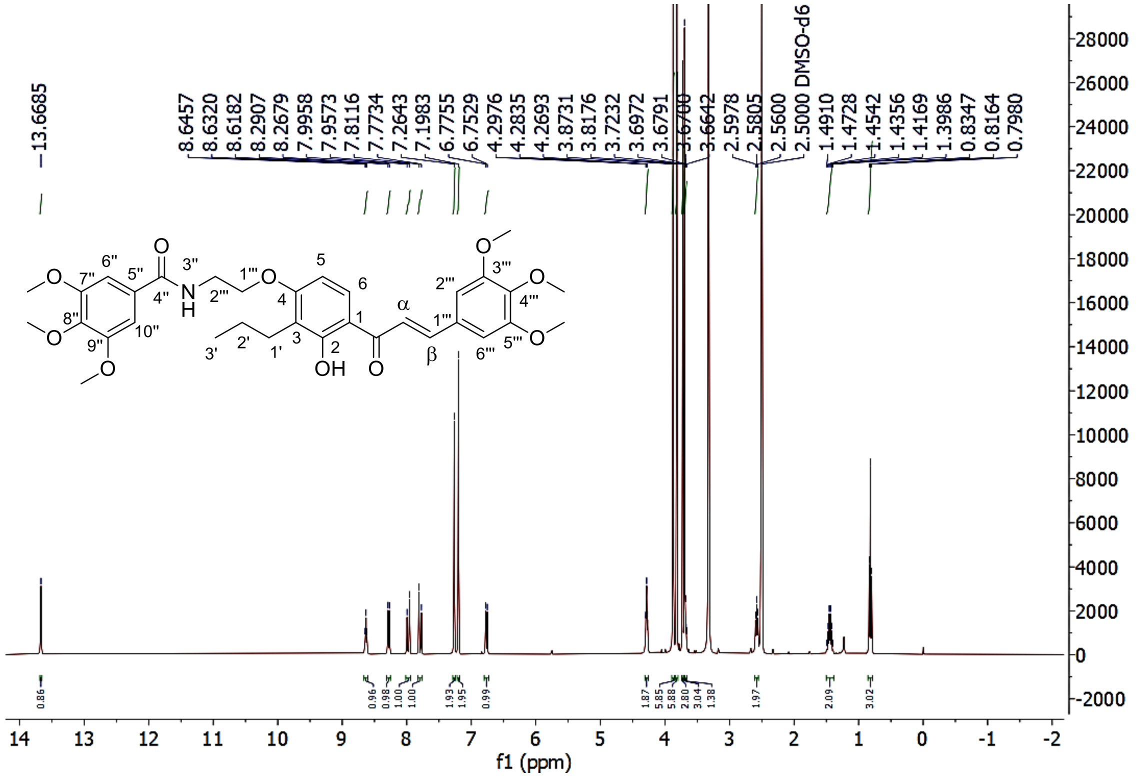

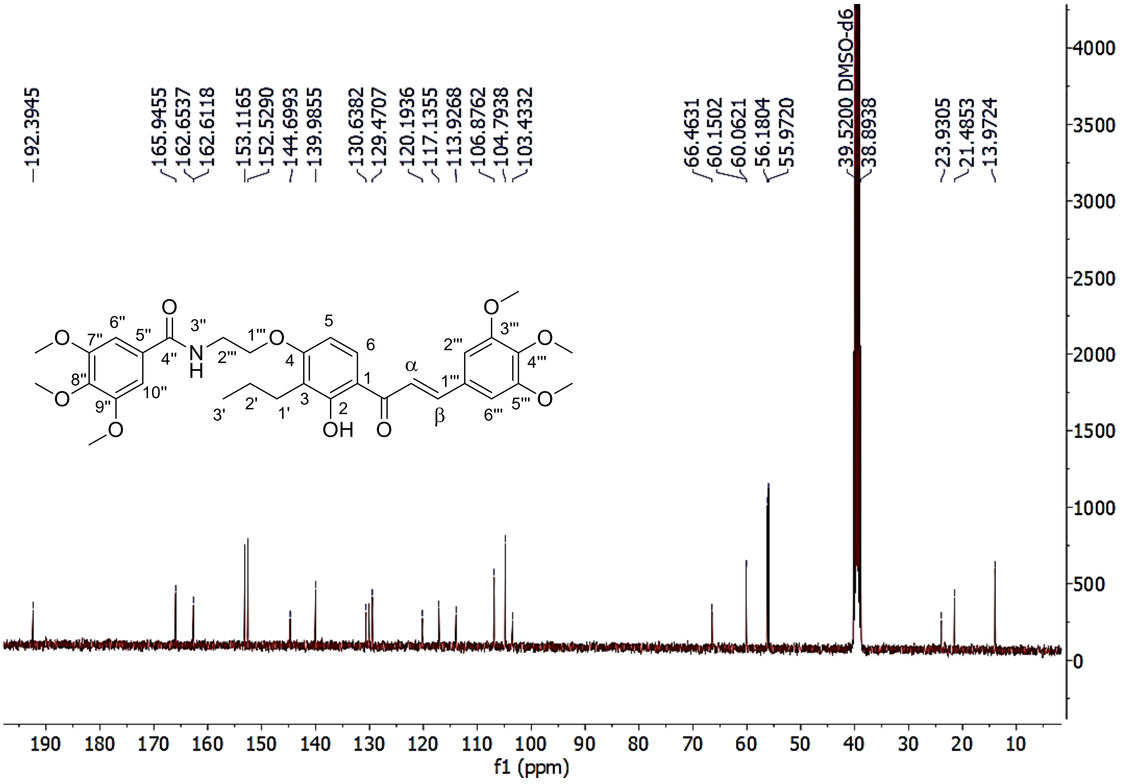

Figure S6. ^{1}H and ^{13}C NMR spectra of compound **8**.

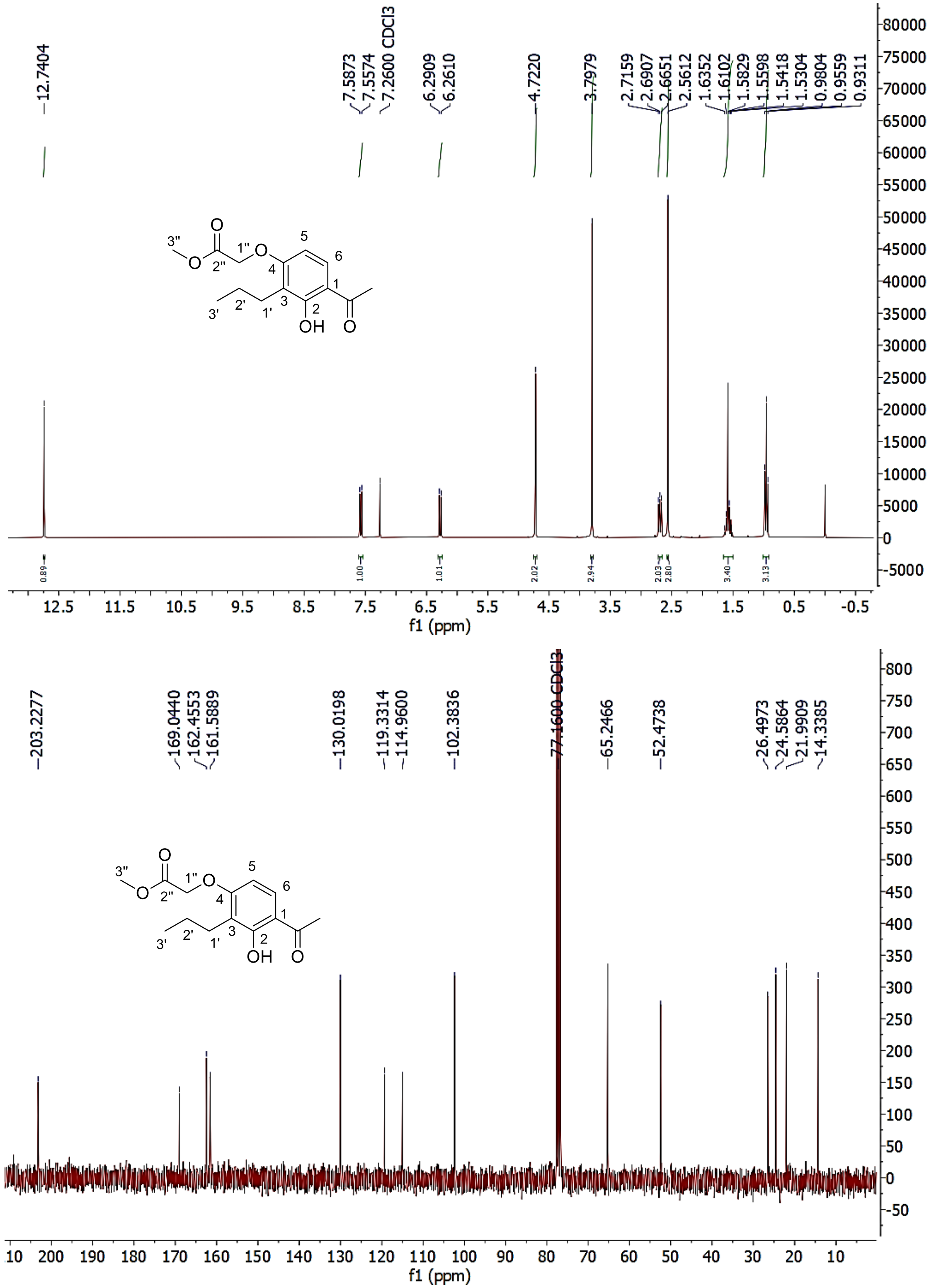

Figure S7. ^{1}H and ^{13}C NMR spectra of compound **9**.

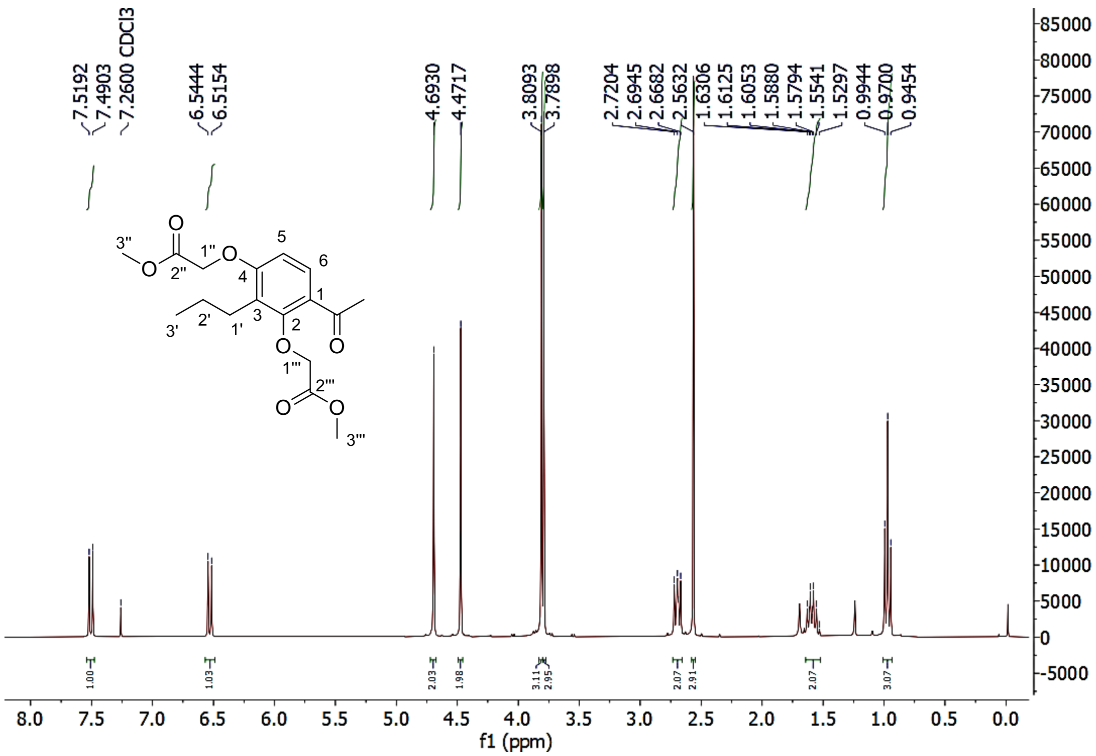

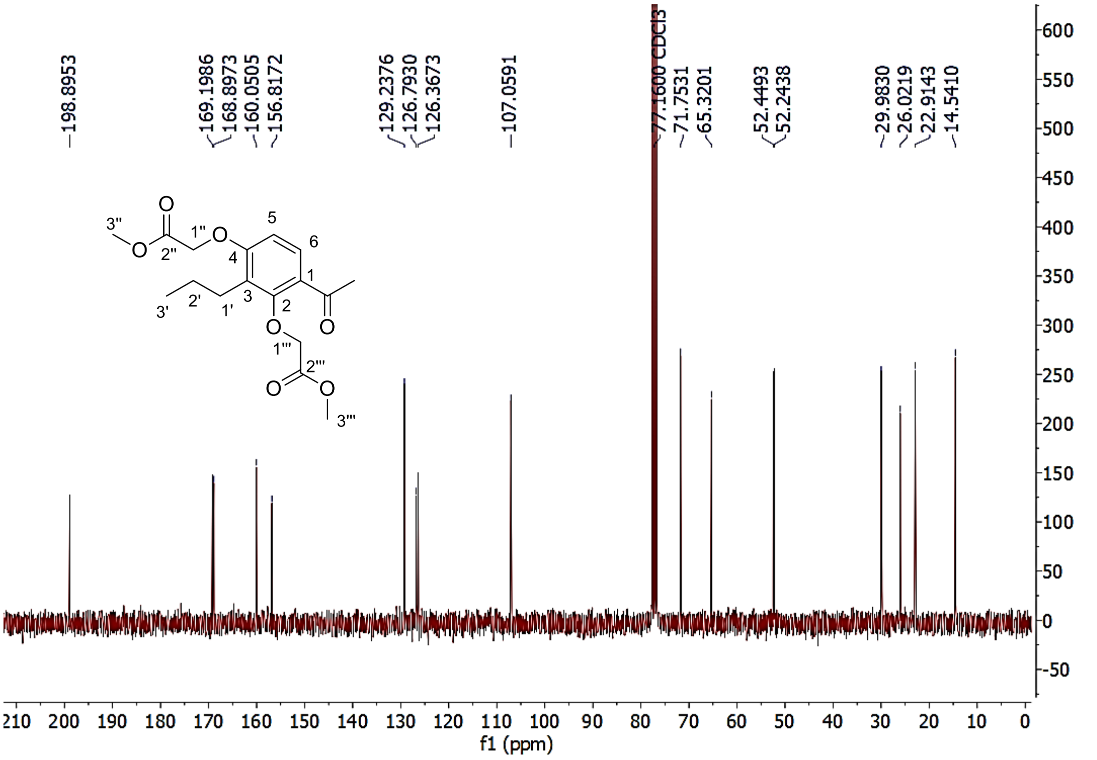

Figure S8. ^{1}H and ^{13}C NMR spectra of compound **10**.

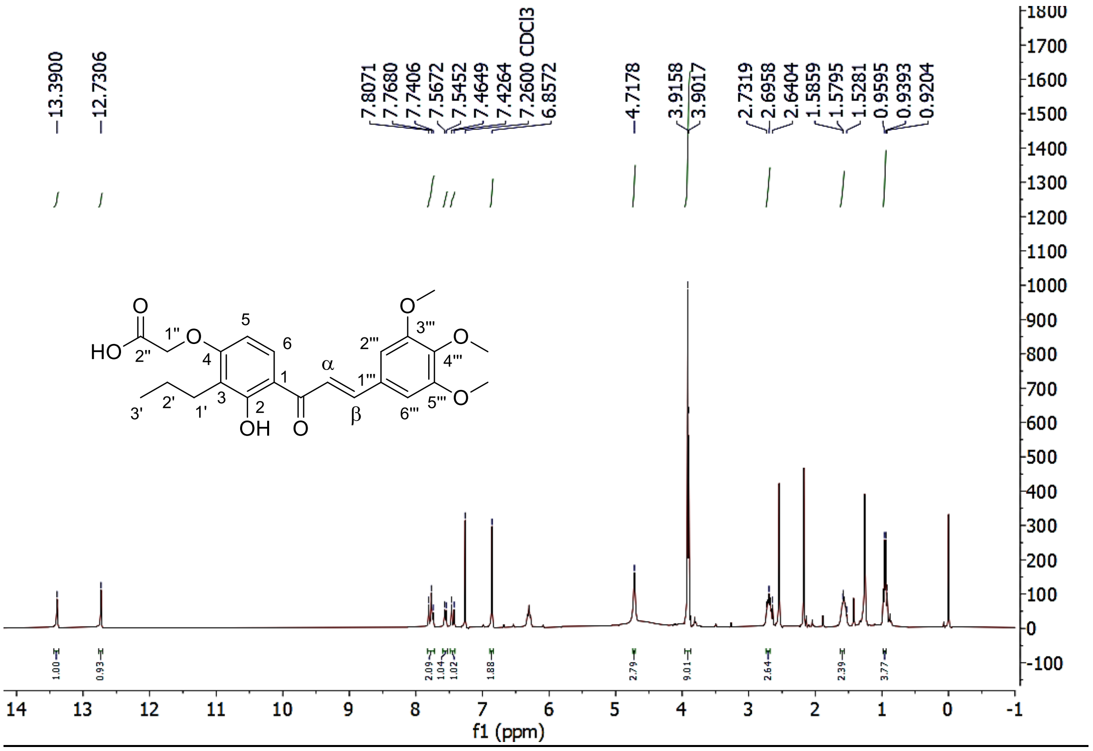

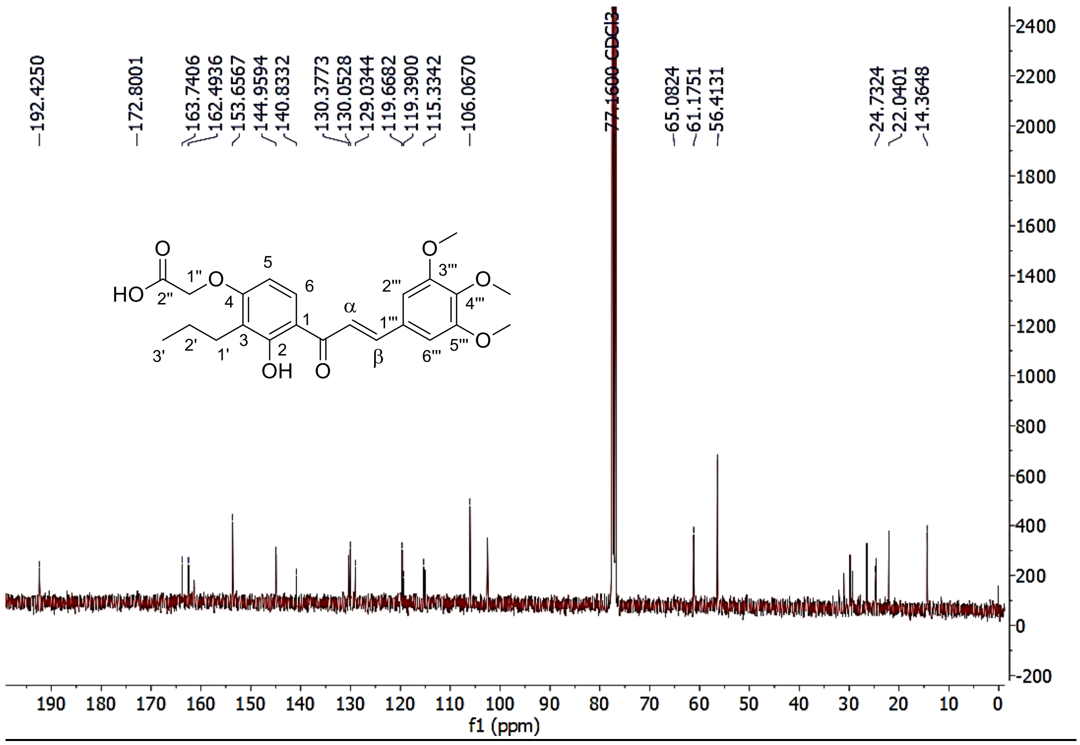

Figure S9. ^{1}H and ^{13}C NMR spectra of compound **11**.

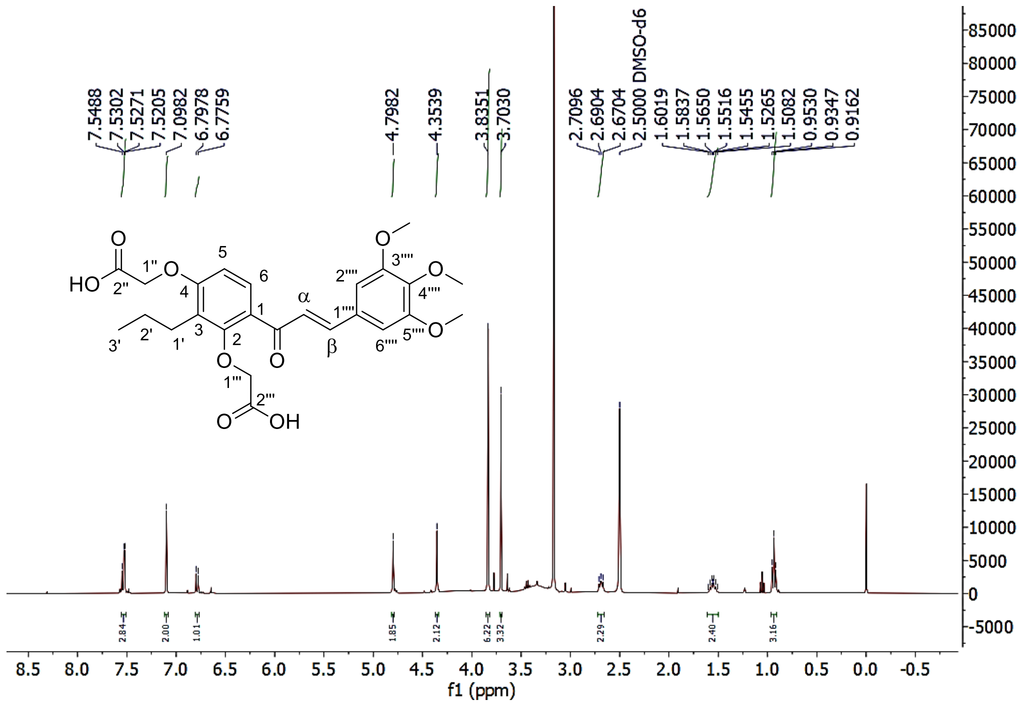

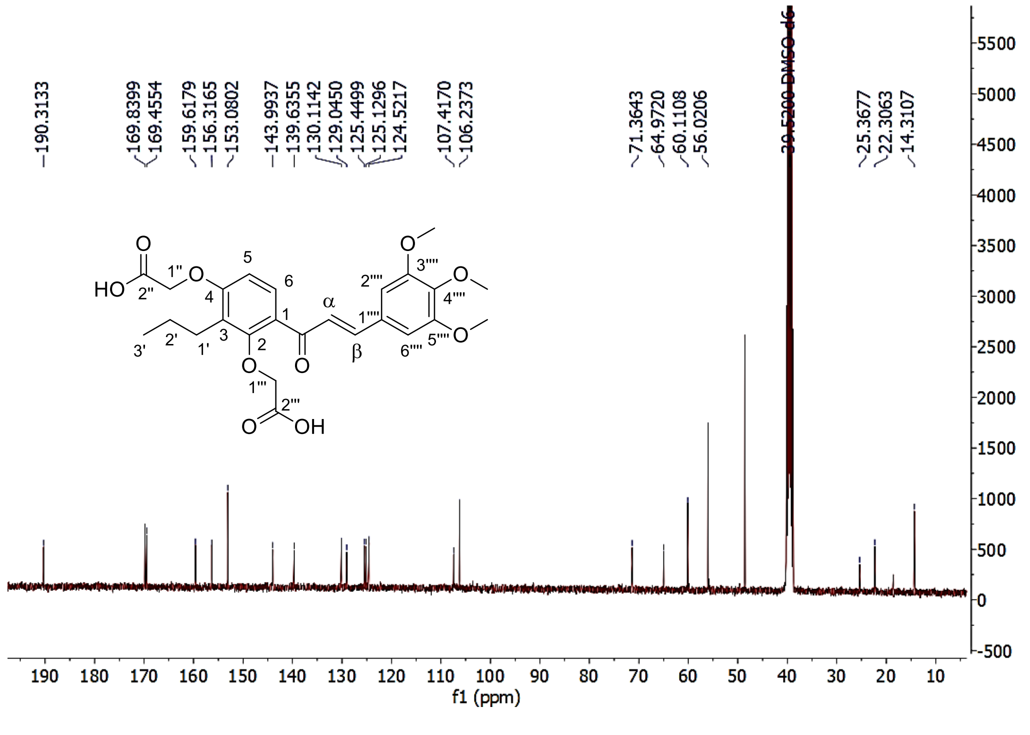

Figure S10. ^{1}H and ^{13}C NMR spectra of compound **12**.

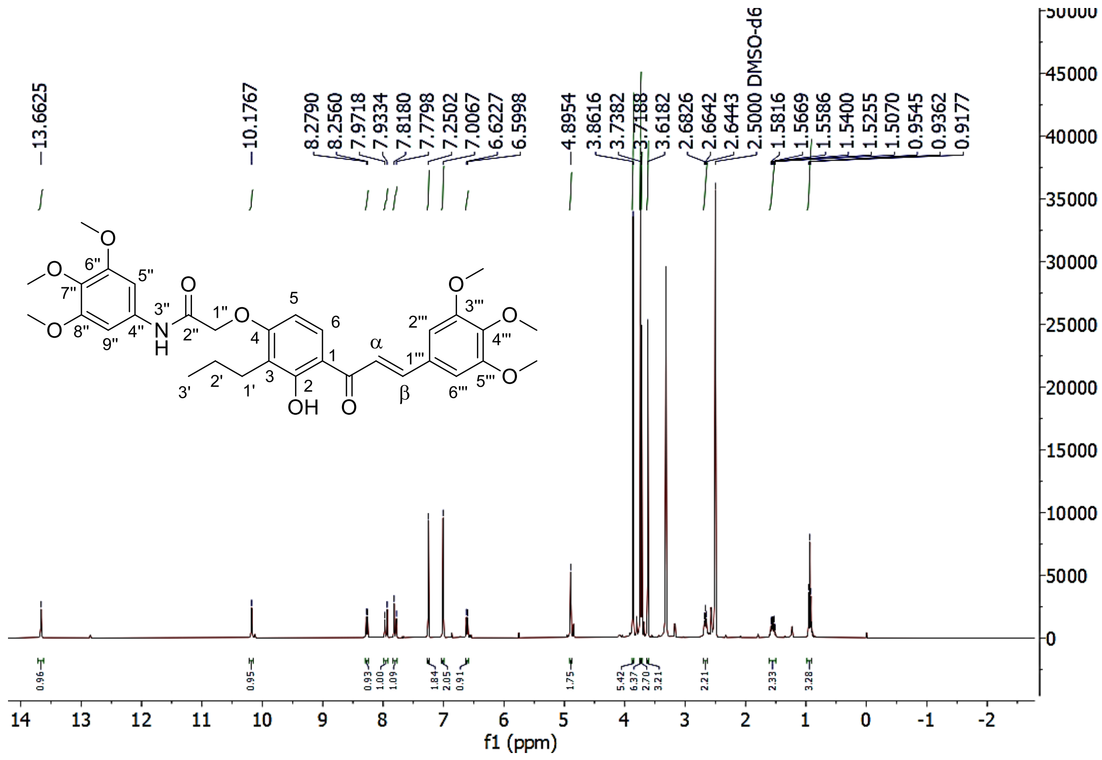

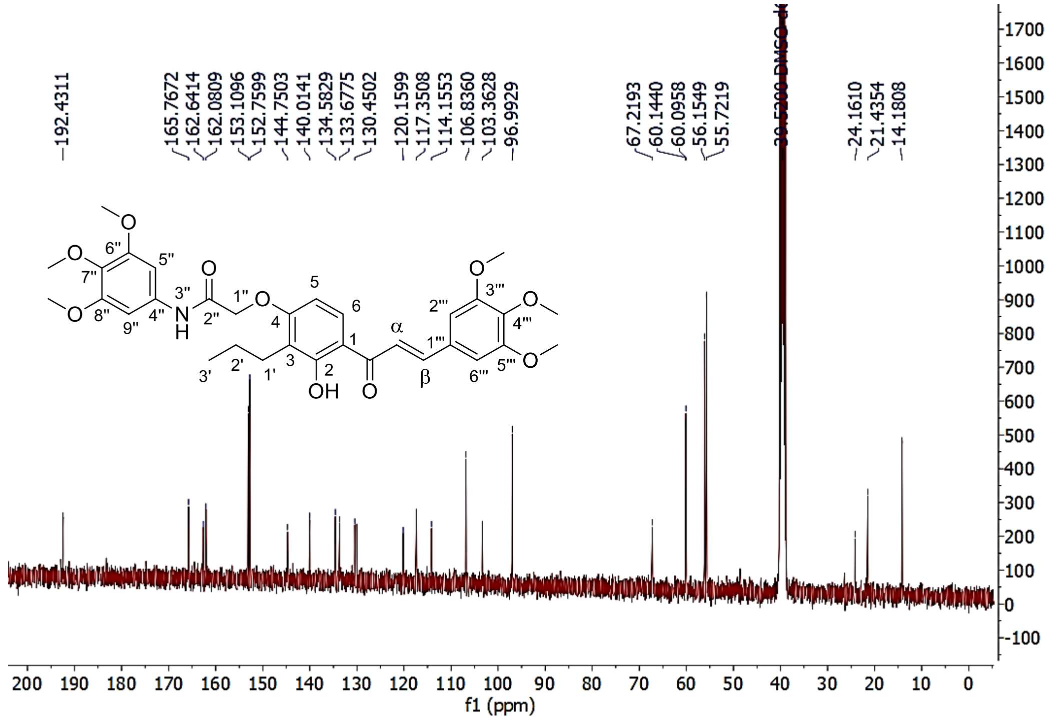

Figure S11. ^{1}H and ^{13}C NMR spectra of compound **13**.

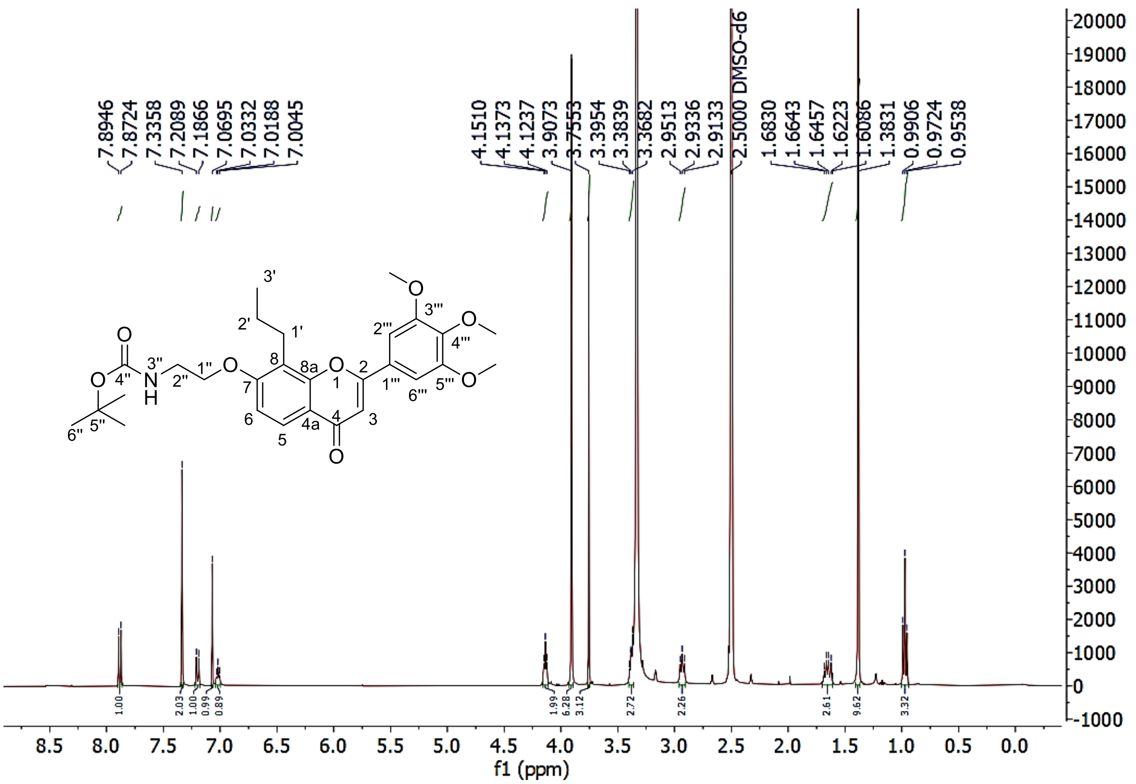

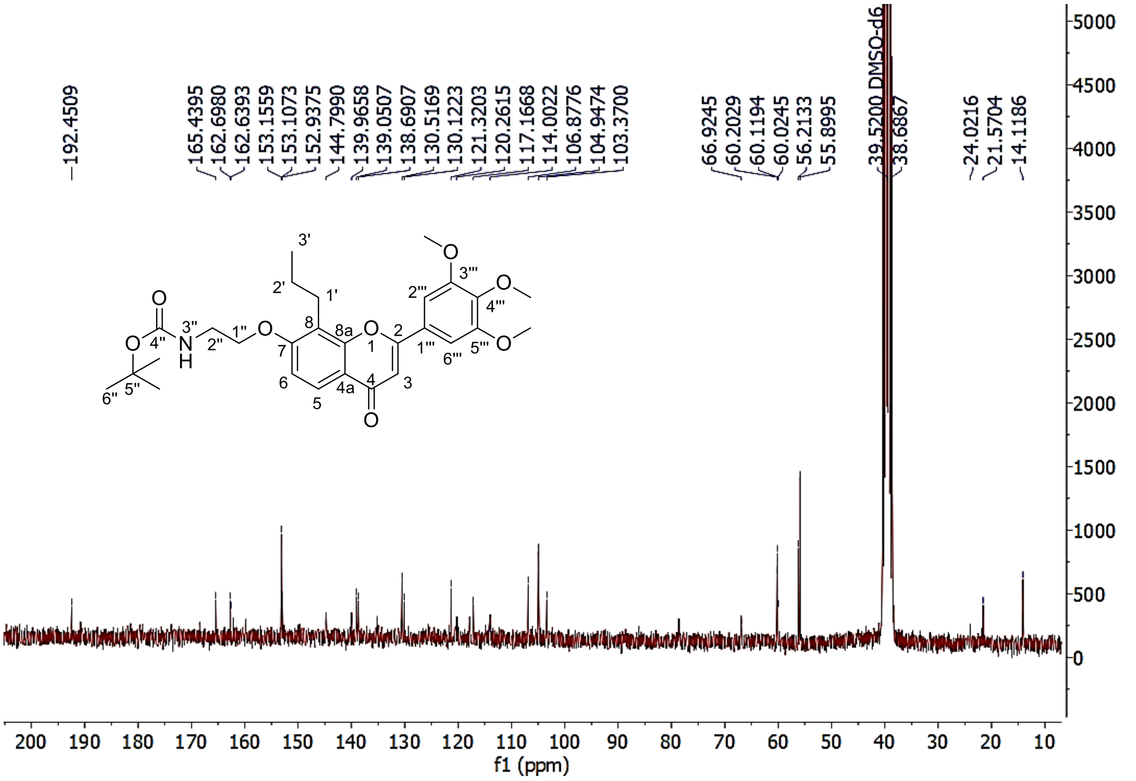

Figure S12. ^{1}H and ^{13}C NMR spectra of compound **14**.

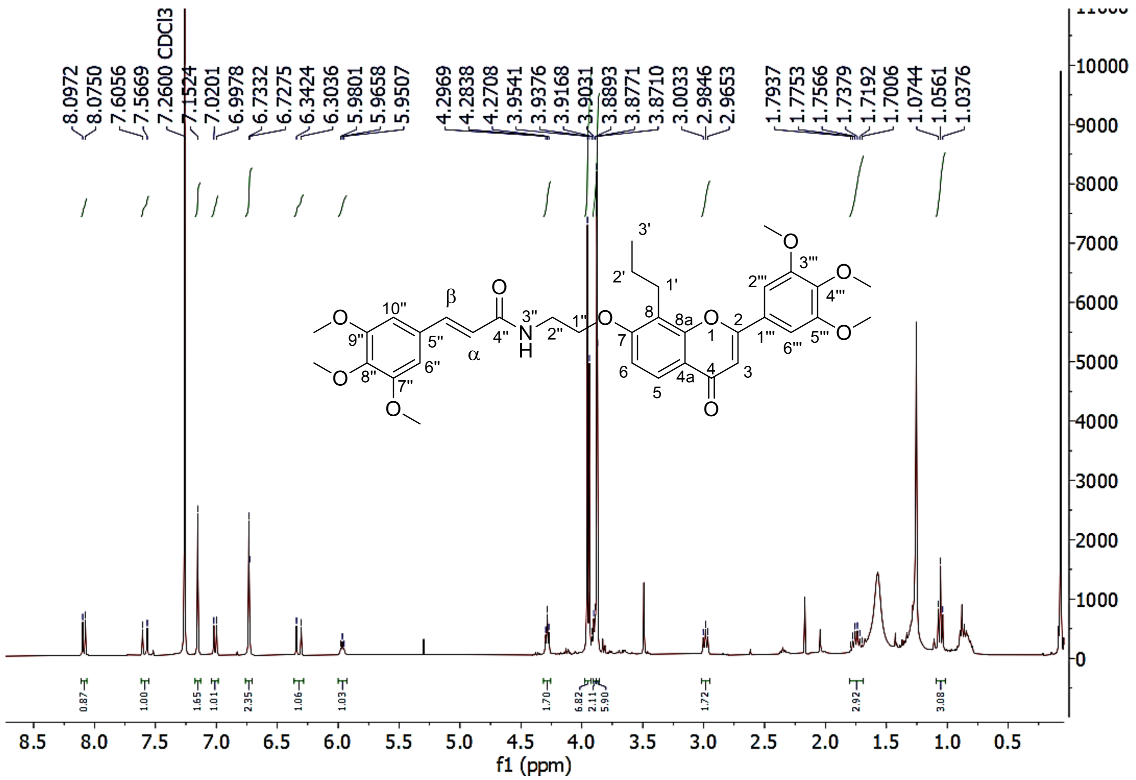

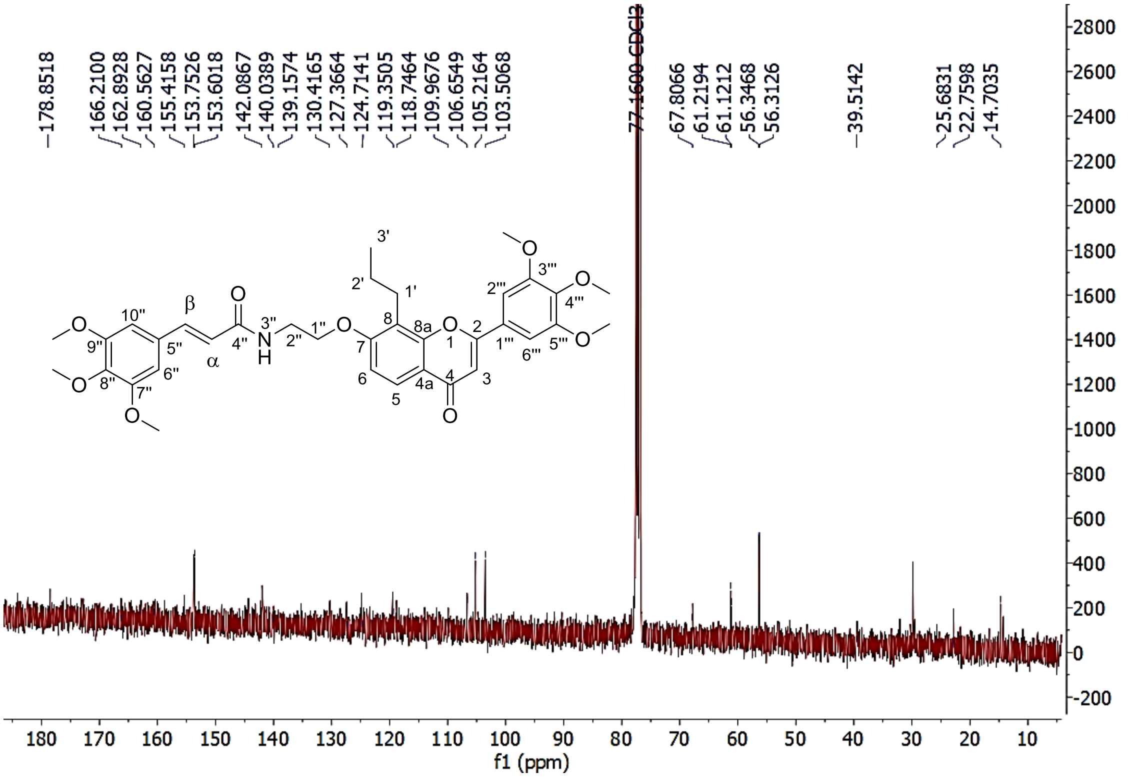

Figure S13. ^{1}H and ^{13}C NMR spectra of compound **15**.

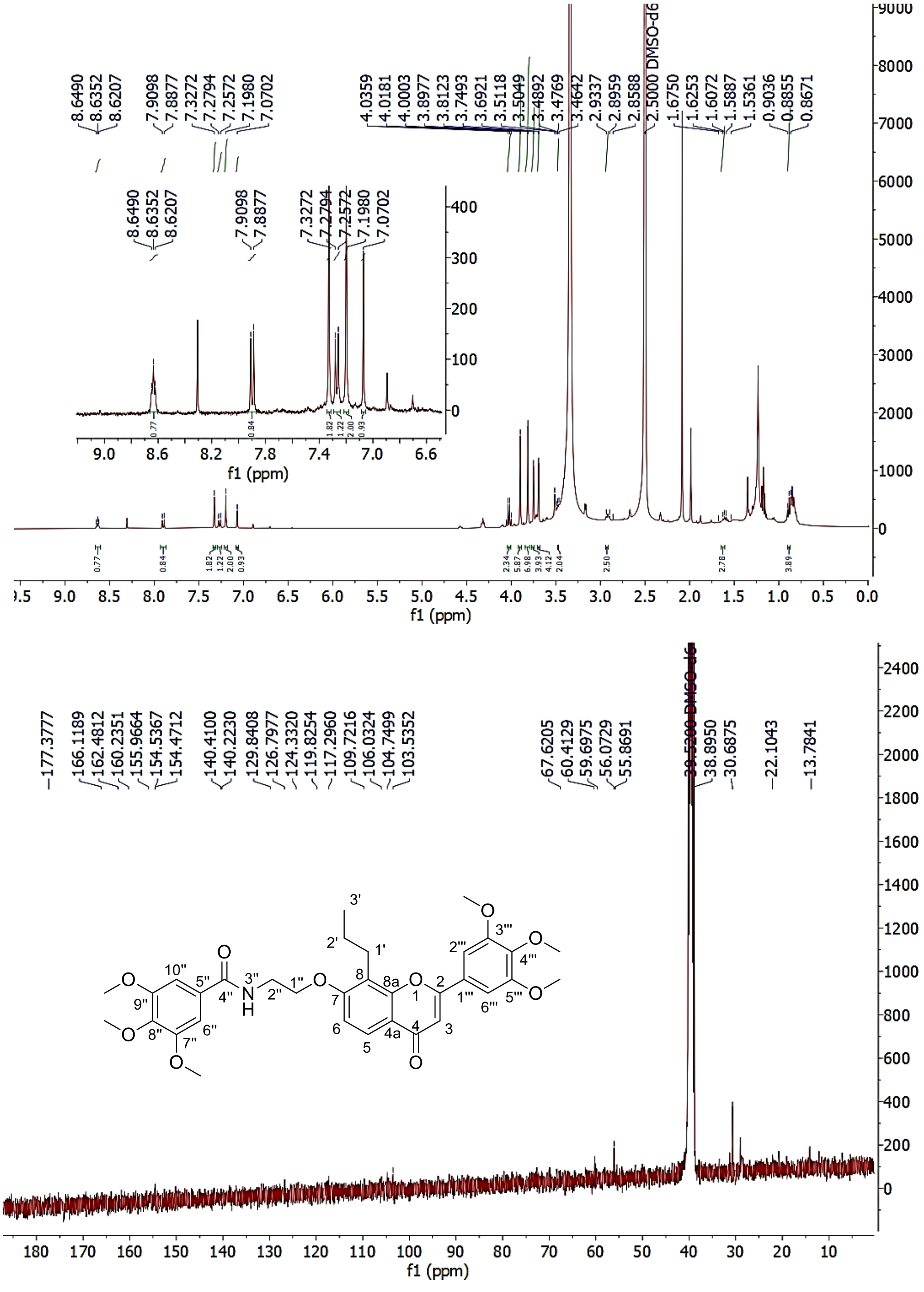

Figure S14. ^{1}H and ^{13}C NMR spectra of compound **16.**

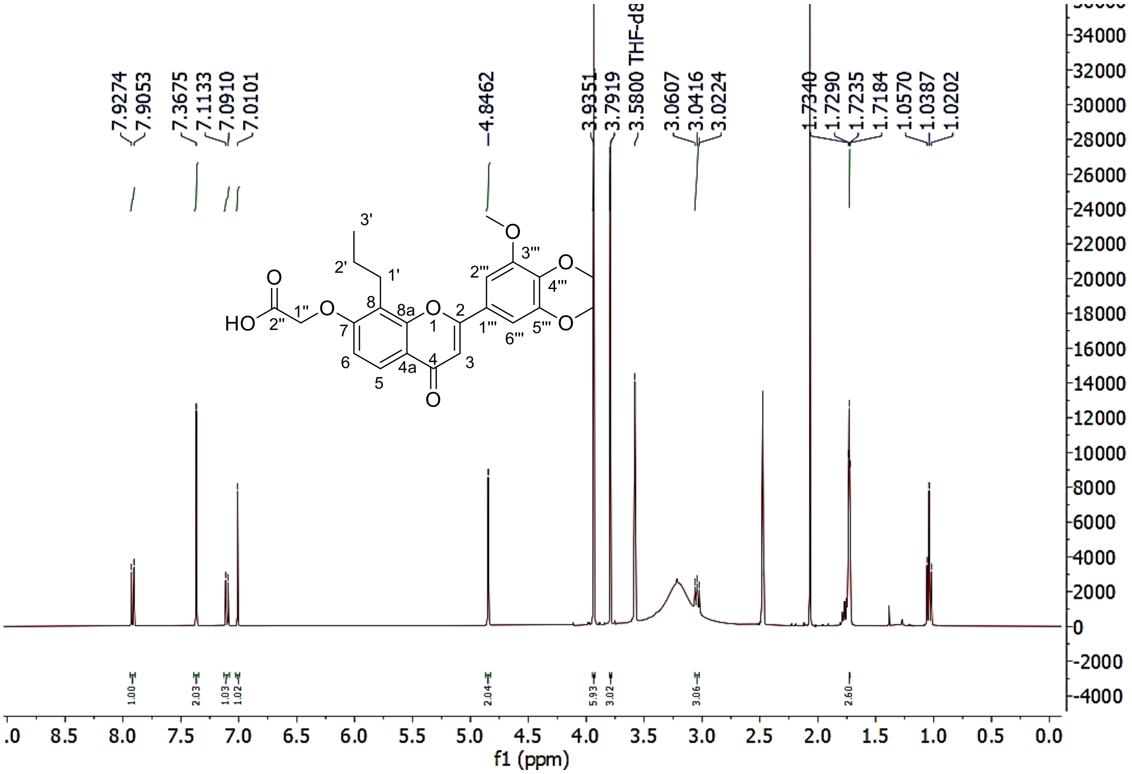

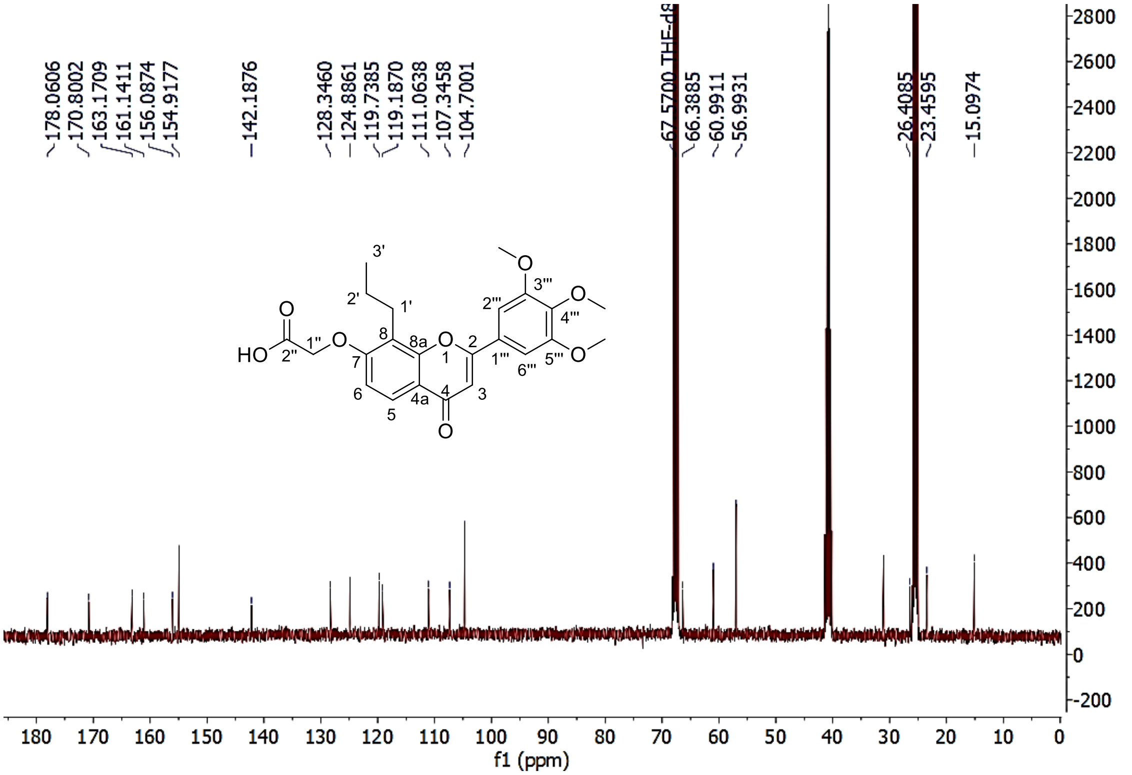

Figure S15. ^{1}H and ^{13}C NMR spectra of compound **17**.

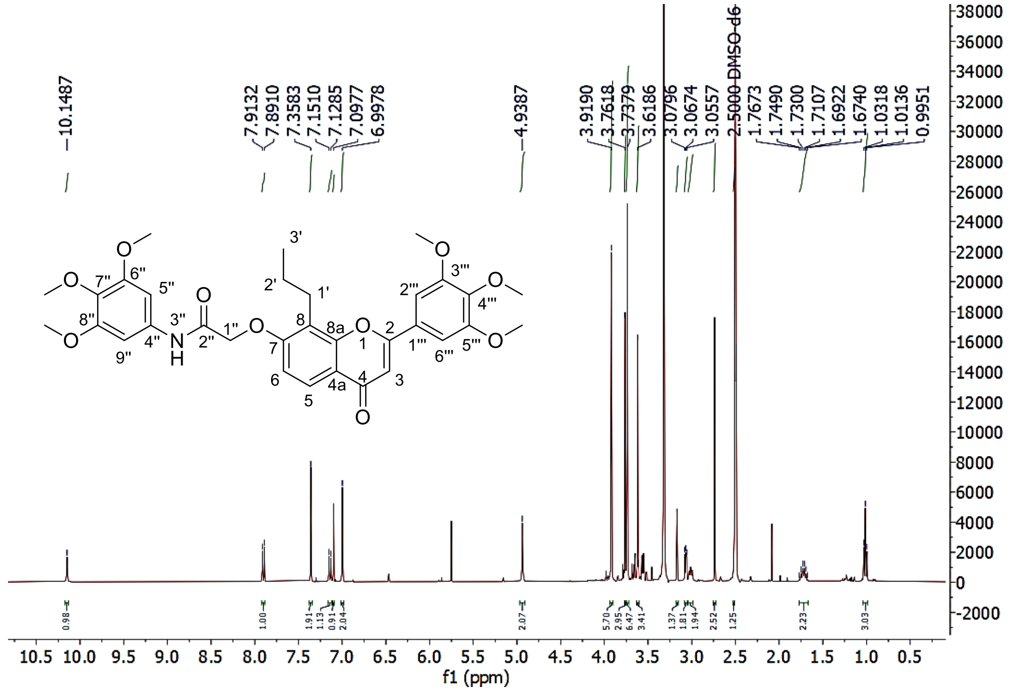

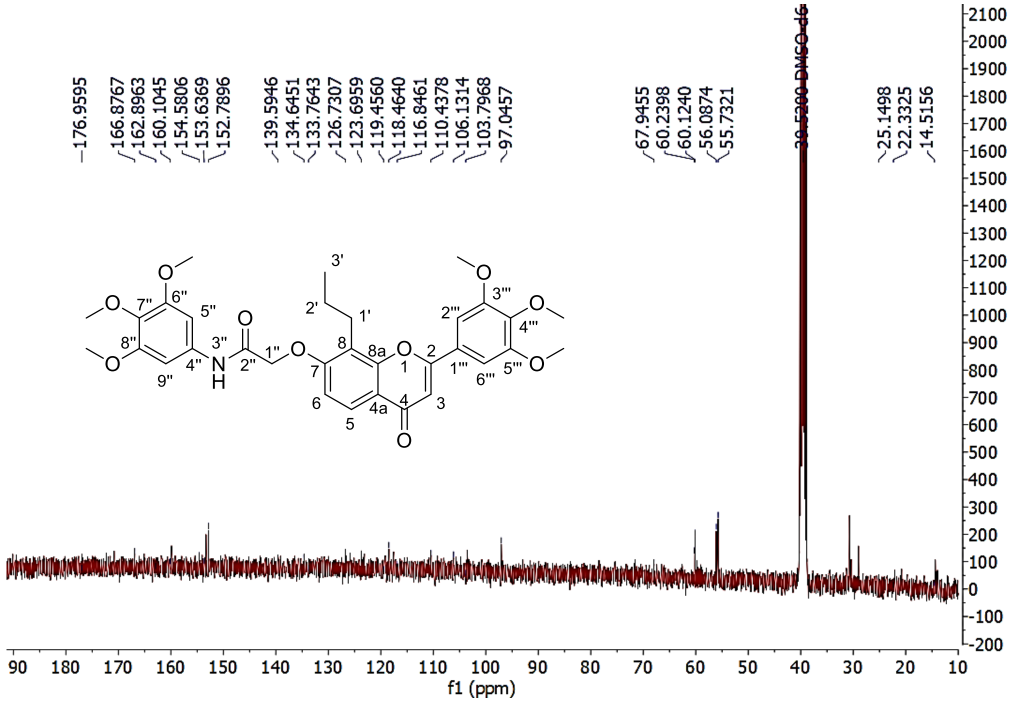

Figure S16. ^{1}H and ^{13}C NMR spectra of compound **18**.

HRMS spectra

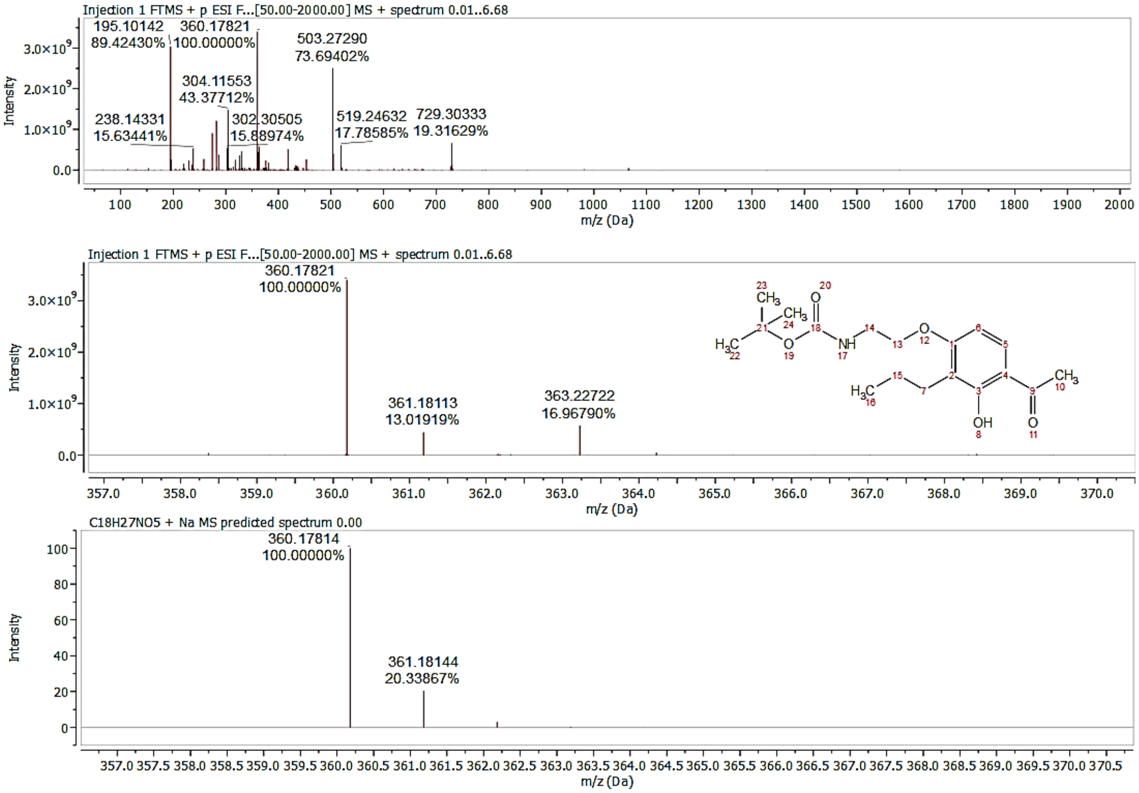

Meas. m/z	Ion formula	m/z	Err [ppm]	Err [mDa]
360.17821	$C_{18}H_{27}NO_5$	360.17814	-0.18	-0.07

Figure S17. HRMS of compound **5**.

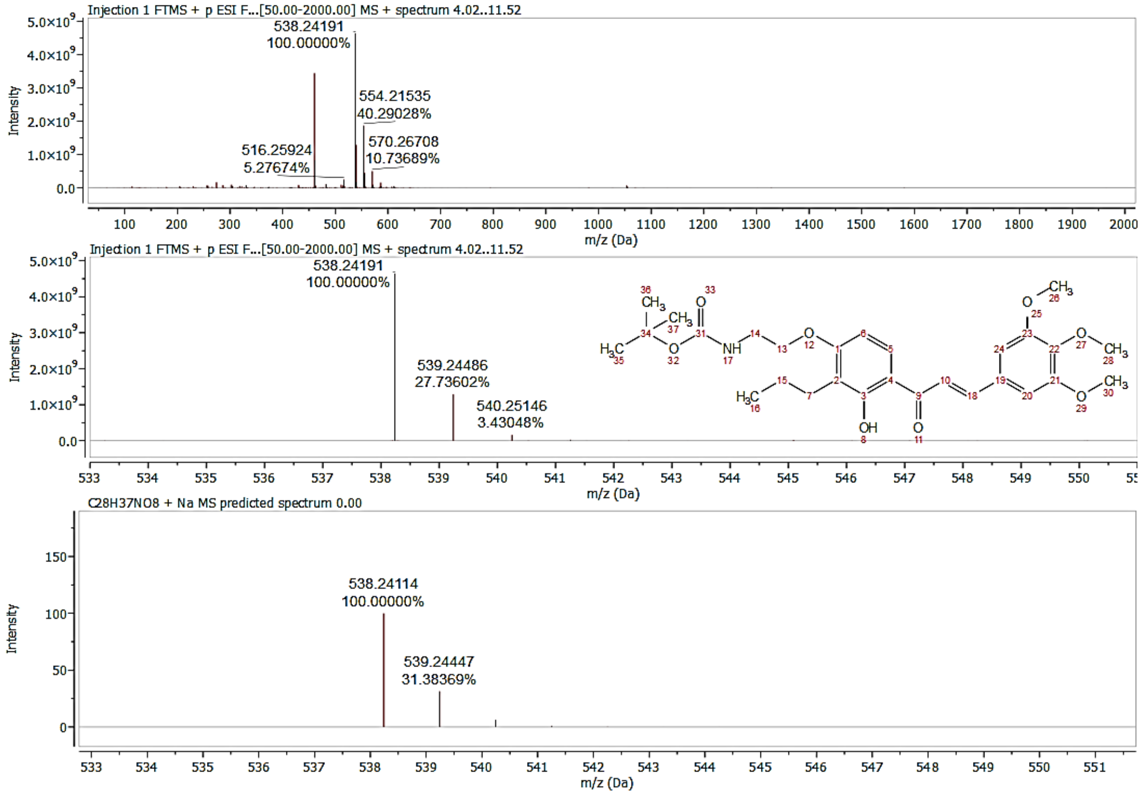

Meas. m/z	Ion formula	m/z	Err [ppm]	Err [mDa]
538.24191	$C_{28}H_{37}NO_8$	538.24114	-1.43	-0.77

Figure S18. HRMS of compound **6**.

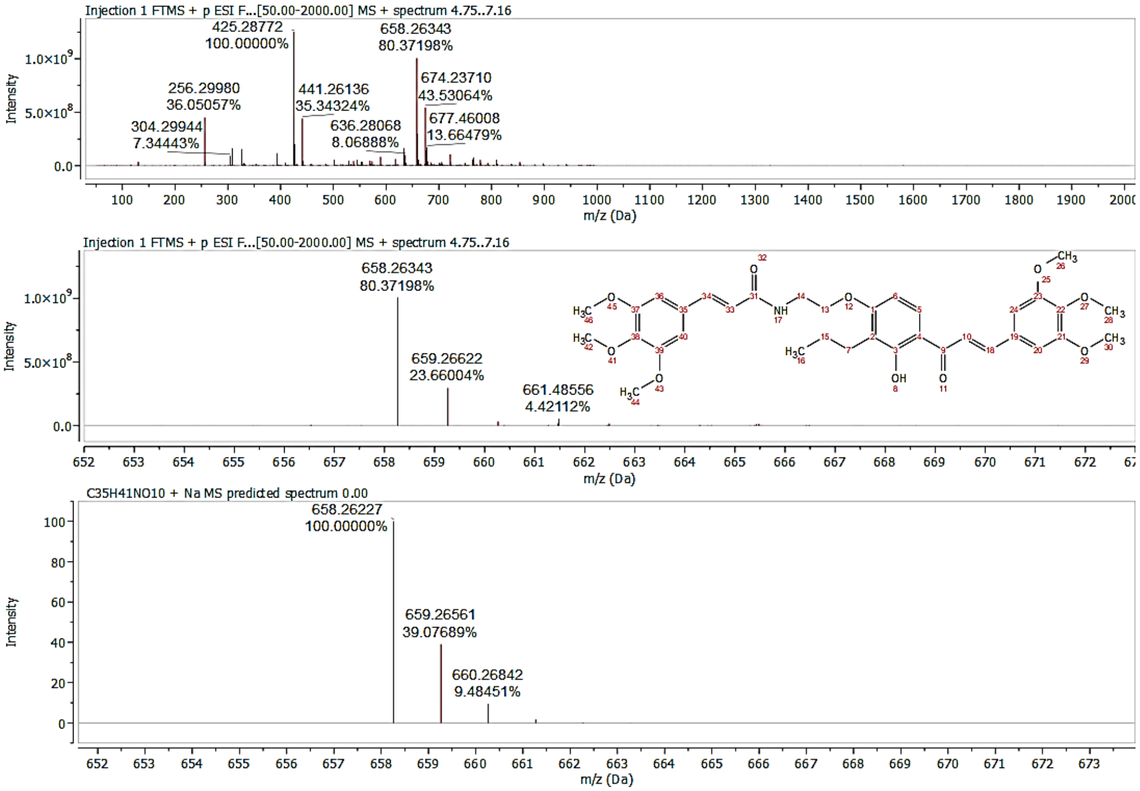

Meas. m/z	Ion formula	m/z	Err [ppm]	Err [mDa]
658.26343	$C_{35}H_{41}NO_{10}$	658.26227	-1.76	-1.16

Figure S19. HRMS of compound **7**.

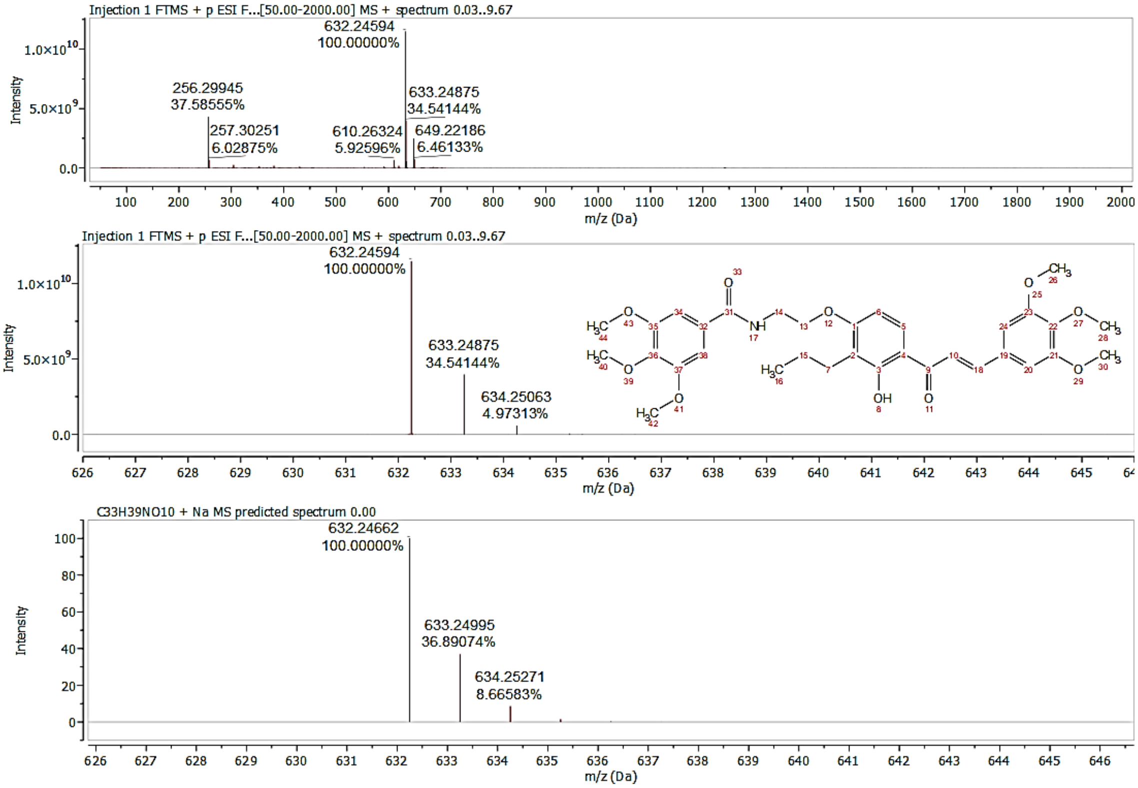

Meas. m/z	Ion formula	m/z	Err [ppm]	Err [mDa]
632.24594	$C_{33}H_{39}NO_{10}$	632.24662	1.07	0.68

Figure S20. HRMS of compound **8**.

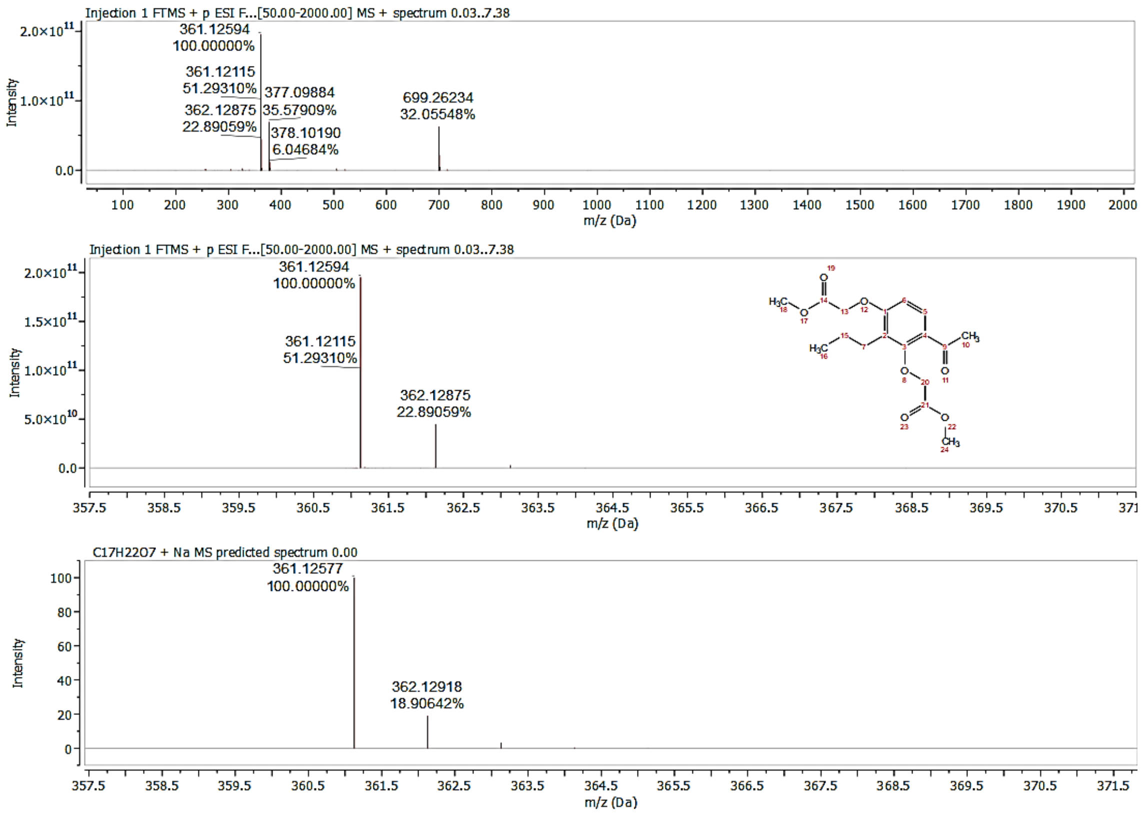

Meas. m/z	Ion formula	m/z	Err [ppm]	Err [mDa]
361.12594	$C_{17}H_{22}O_7$	361.12577	-0.46	-0.16

Figure S21. HRMS of compound **10**.

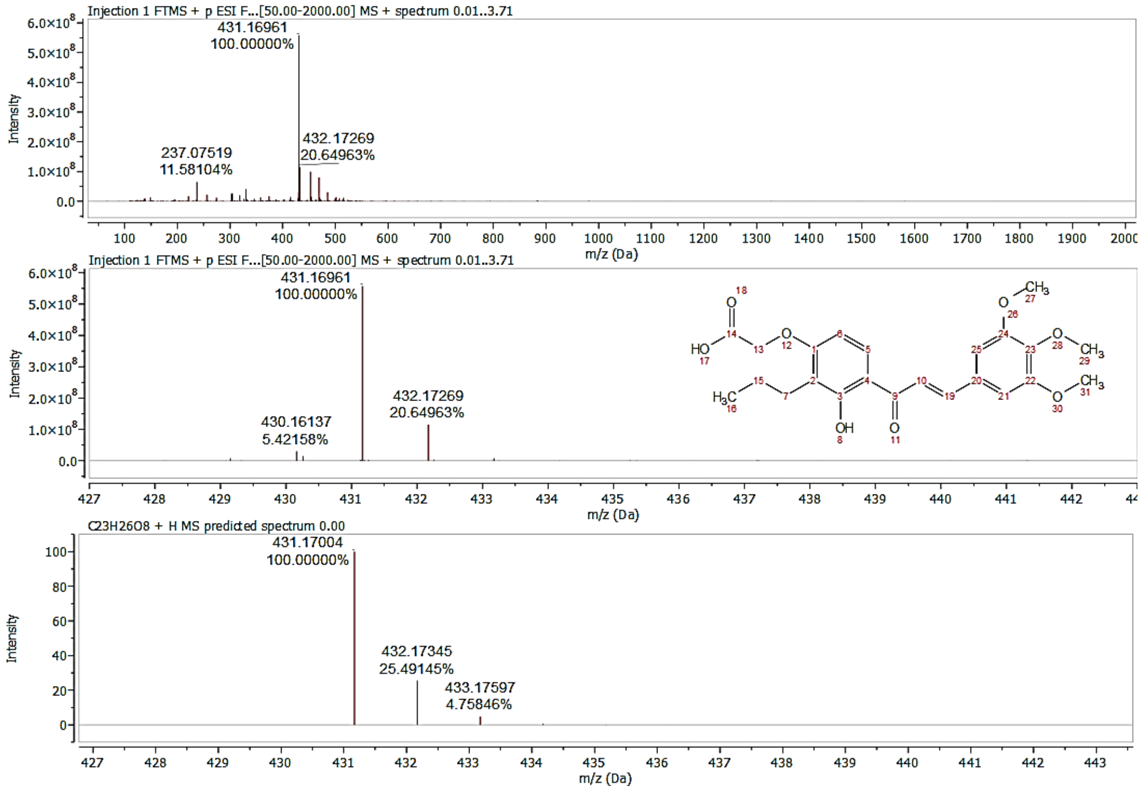

Meas. m/z	Ion formula	m/z	Err [ppm]	Err [mDa]
431.16961	$C_{23}H_{26}O_{10}$	431.17004	1.01	0.44

Figure S22. HRMS of compound **11**.

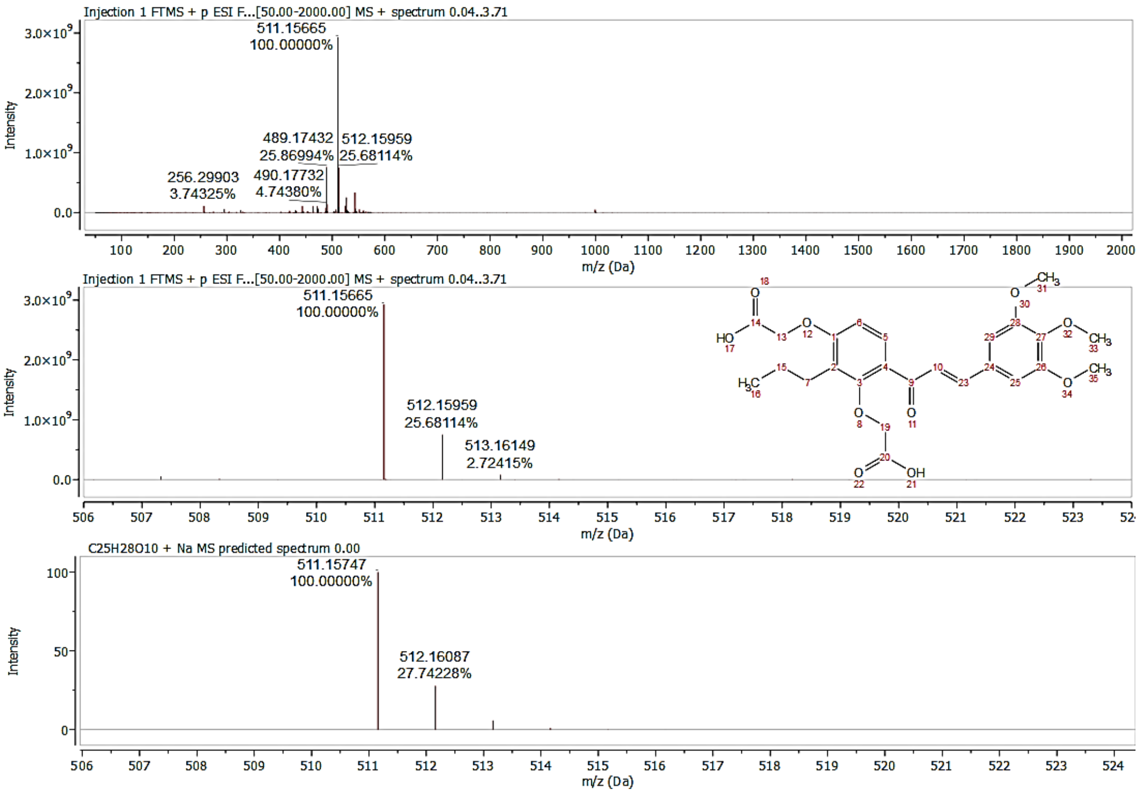

Meas. m/z	Ion formula	m/z	Err [ppm]	Err [mDa]
511.15885	$C_{25}H_{28}O_{10}$	511.15747	-1.43	-0.77

Figure S23. HRMS of compound **12**.

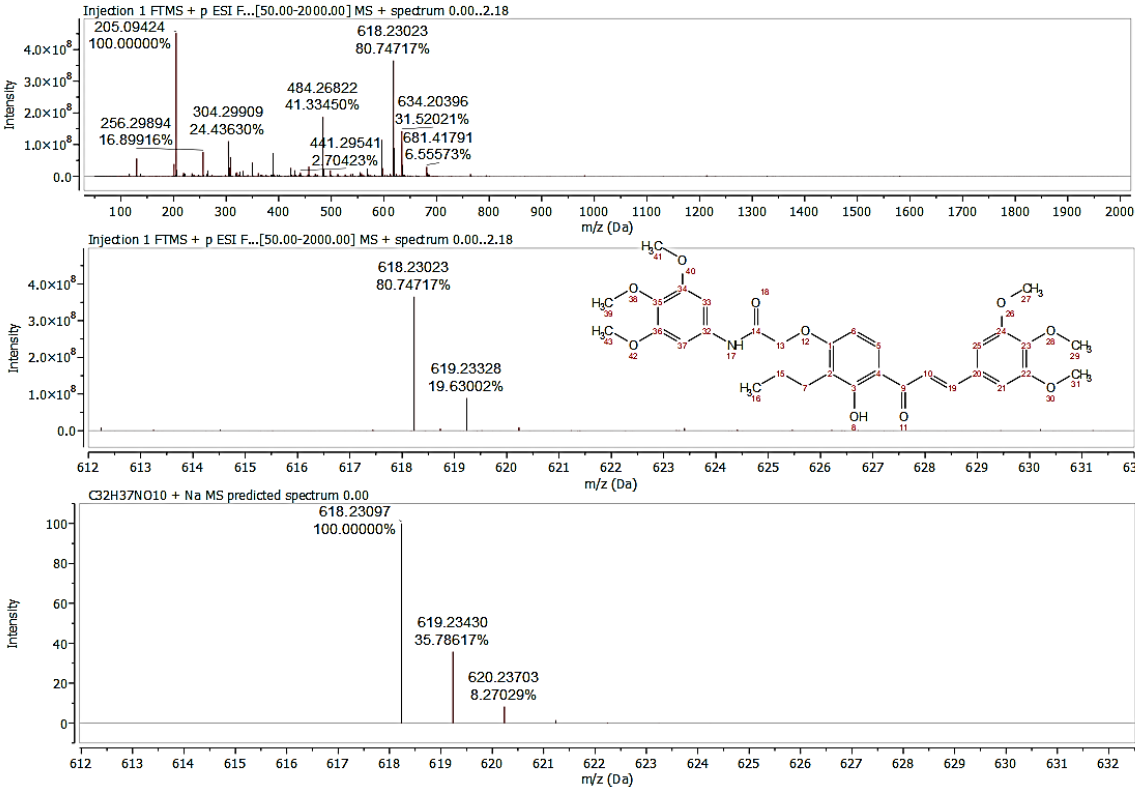

Meas. m/z	Ion formula	m/z	Err [ppm]	Err [mDa]
618.23023	$C_{32}H_{37}NO_{10}$	618.23097	1.07	0.68

Figure S24. HRMS of compound **13**.

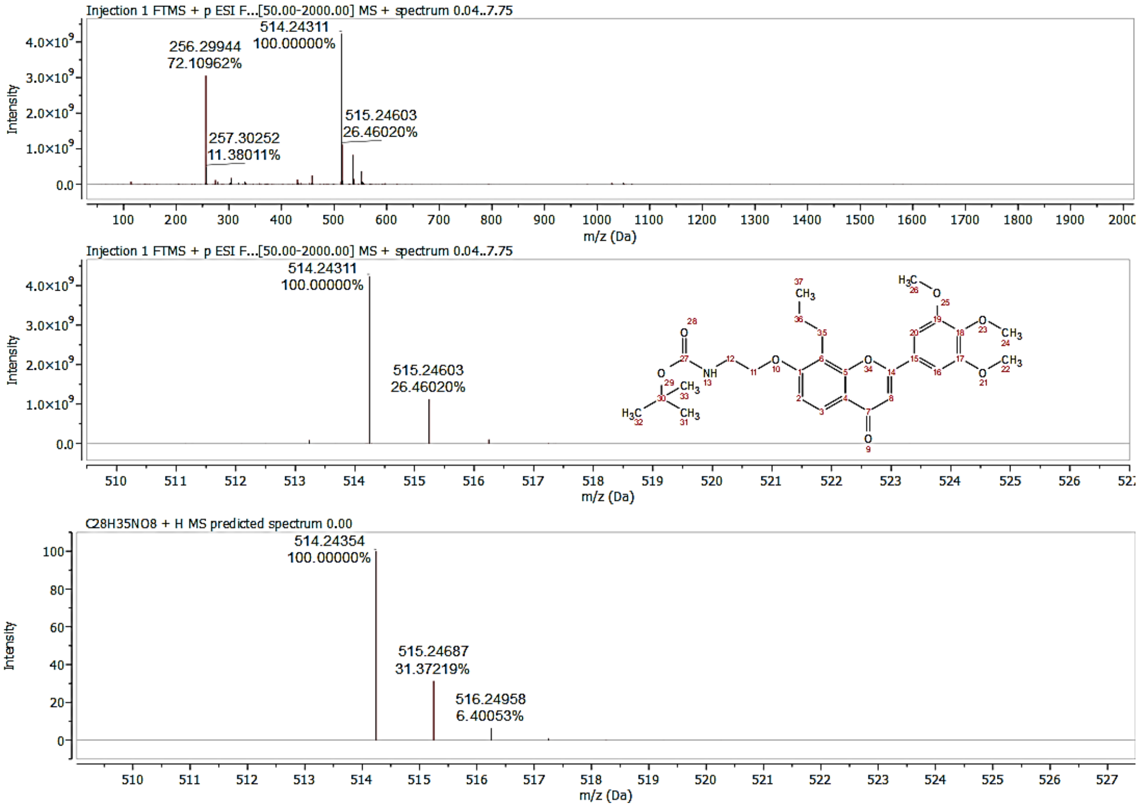

Meas. m/z	Ion formula	m/z	Err [ppm]	Err [mDa]
514.24311	$C_{28}H_{35}NO_8$	514.24354	0.85	0.44

Figure S25. HRMS of compound **14**.

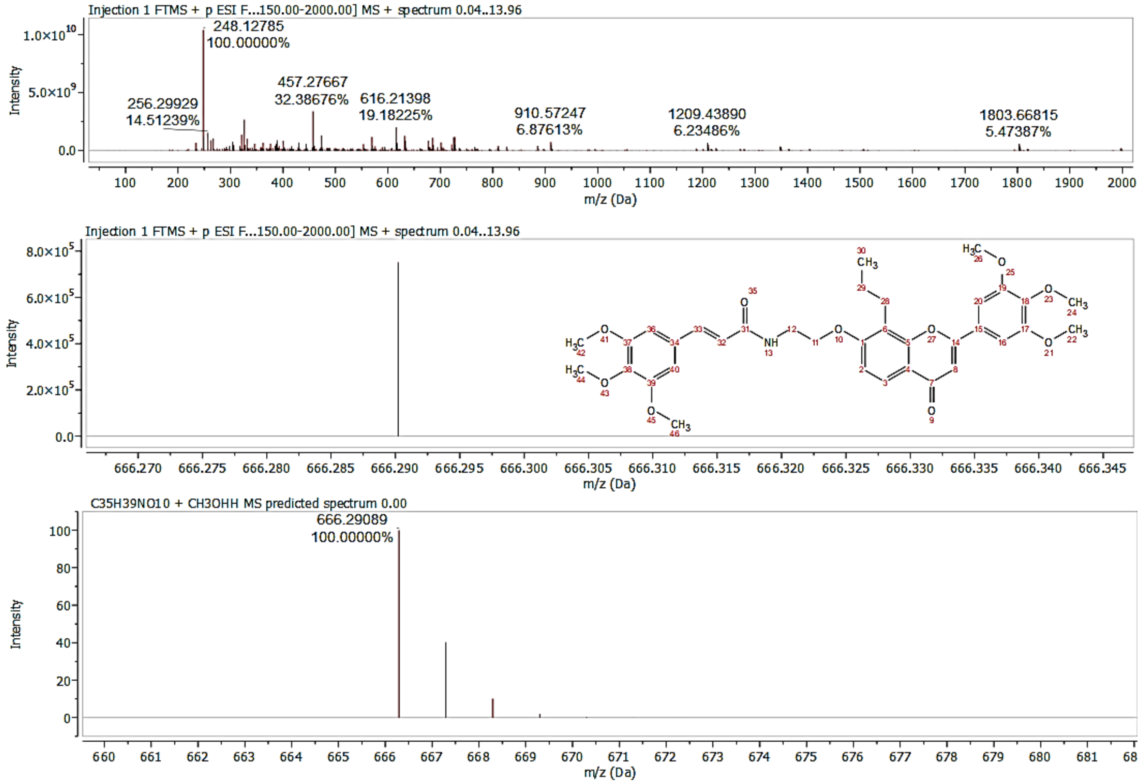

Meas. m/z	Ion formula	m/z	Err [ppm]	Err [mDa]
666.29022	$C_{35}H_{39}NO_{10}$	666.29089	1.01	0.67

Figure S26. HRMS of compound **15**.

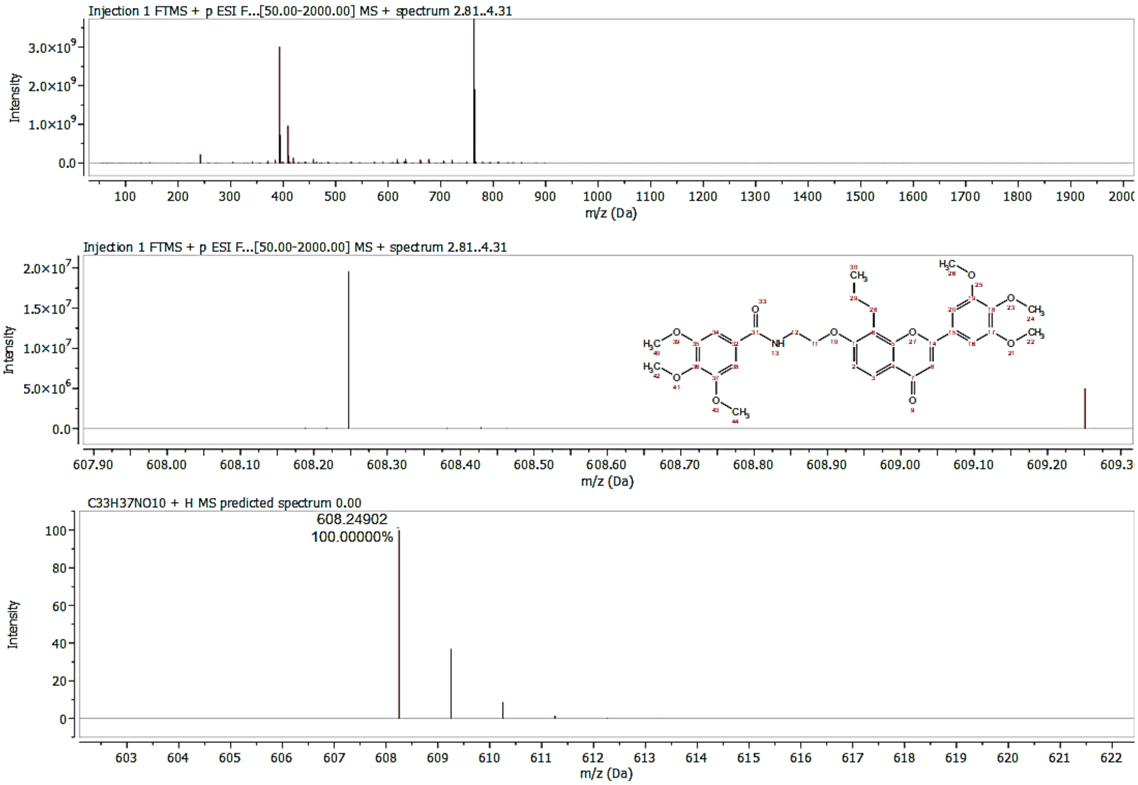

Meas. m/z	Ion formula	m/z	Err [ppm]	Err [mDa]
608.24725	$C_{33}H_{37}NO_{10}$	608.24902	2.92	1.78

Figure S27. HRMS of compound **16.**

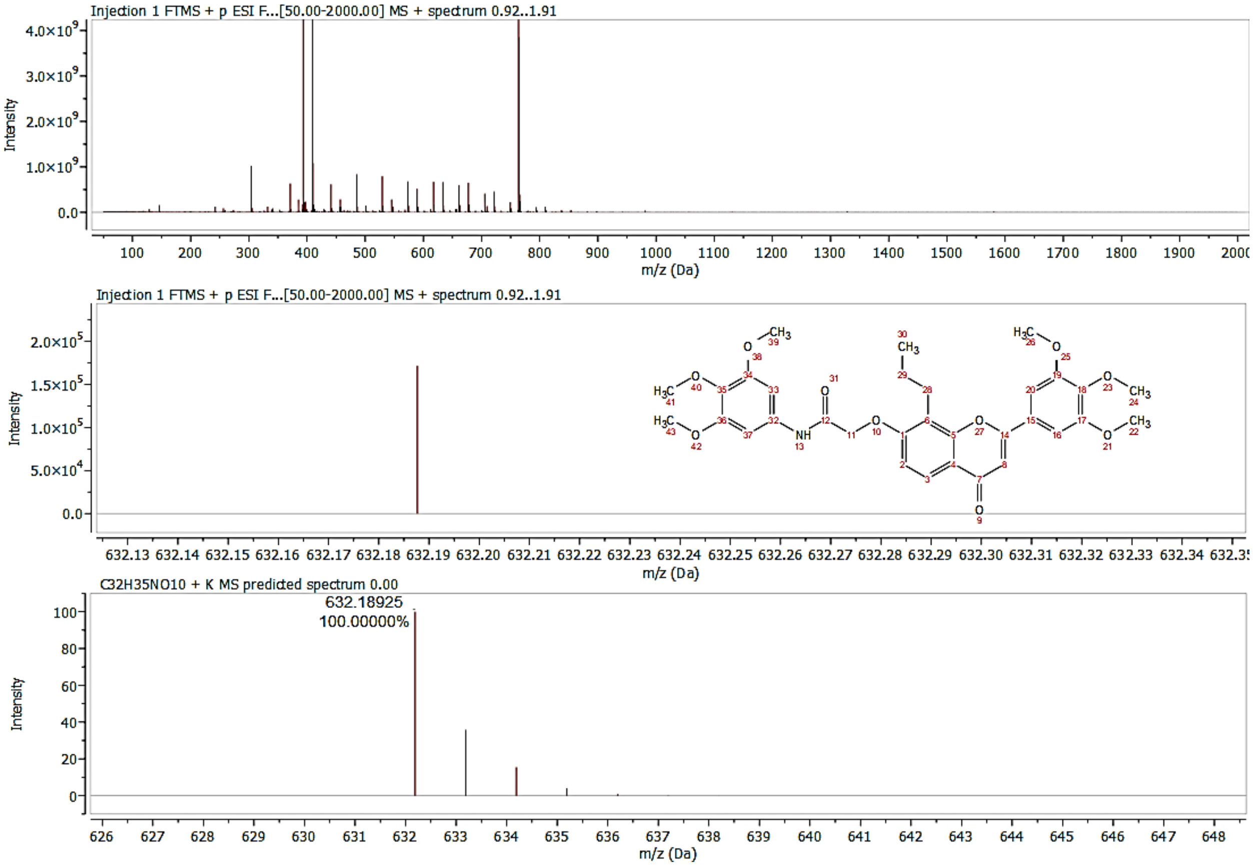

Meas. m/z	Ion formula	m/z	Err [ppm]	Err [mDa]
632.18769	$C_{33}H_{37}NO_{10}$	632.18925	2.48	1.57

Figure S28. HRMS of compound **17**.

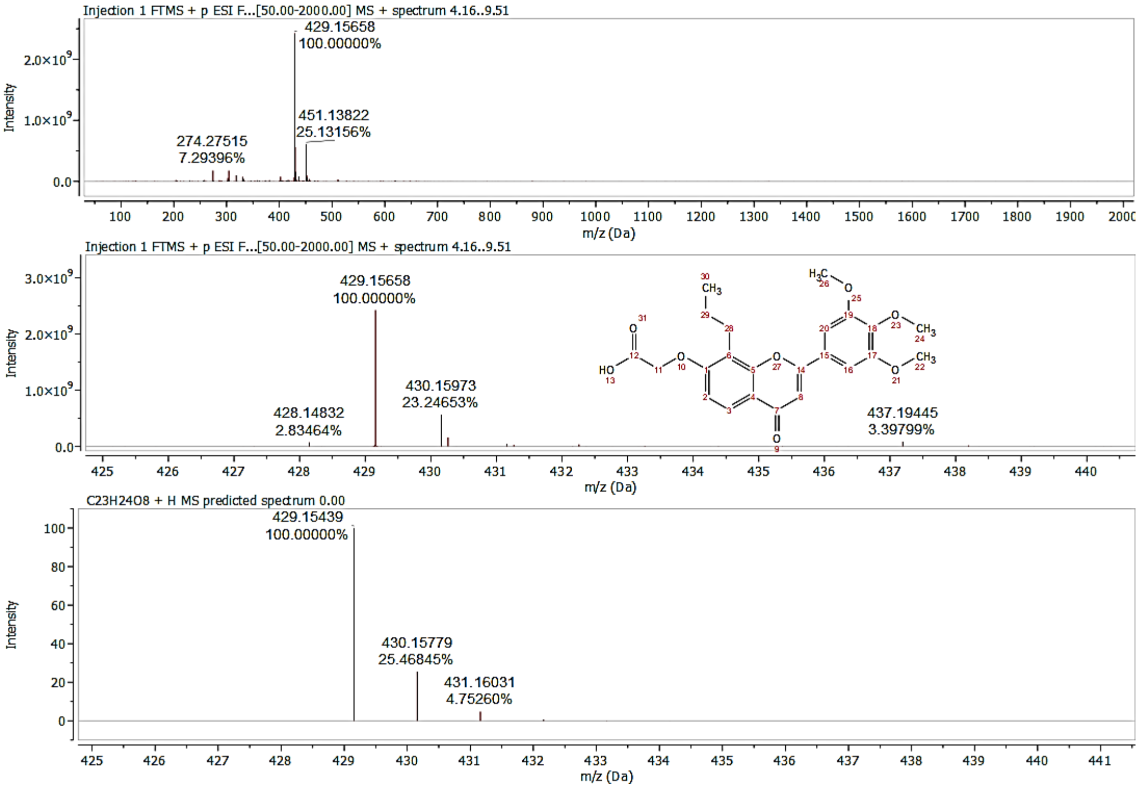

Meas. m/z	Ion formula	m/z	Err [ppm]	Err [mDa]
429.15658	$C_{23}H_{24}O_8$	429.15439	-5.09	-2.18

Figure S29. HRMS of compound **18**.

HPLC analysis

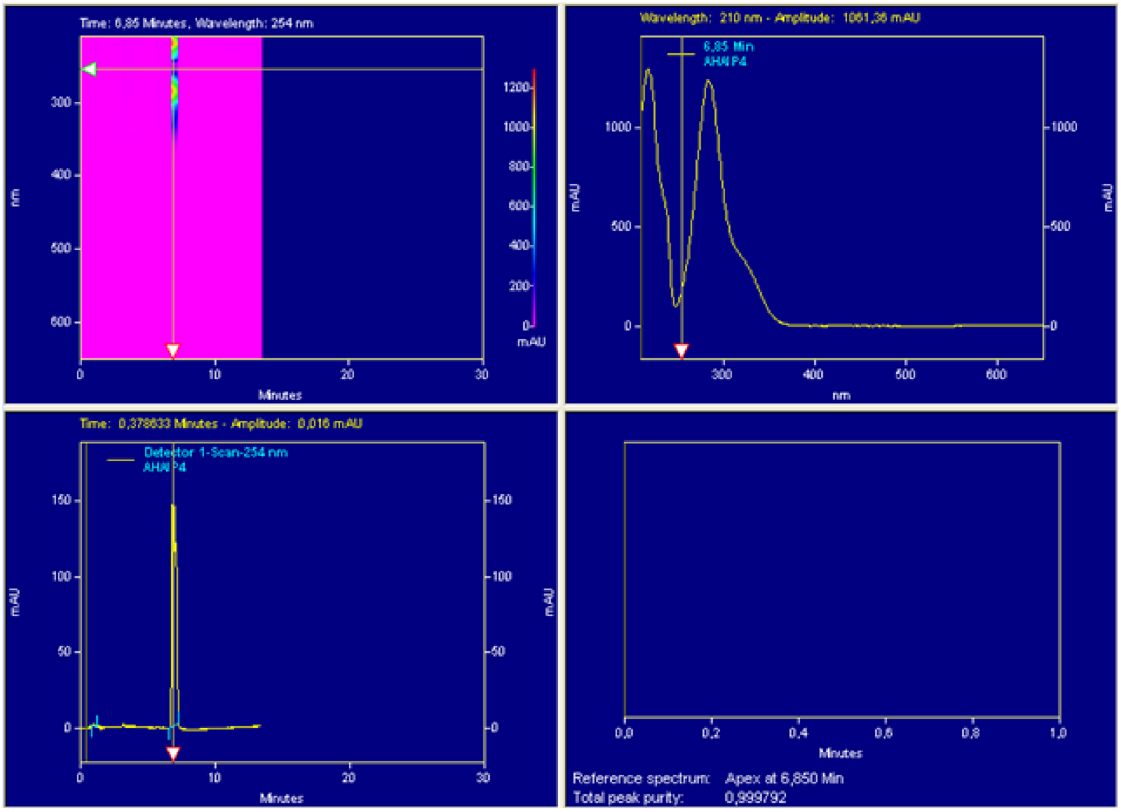

Retention Time	Area	Area %	Height	Height %
0.950	10236	0.30	1253	0.75
6.850	3353498	99.70	165637	99.25
Totals	3363734	100.00	166890	100.00

Figure S30. Peak purity of compound **5**.

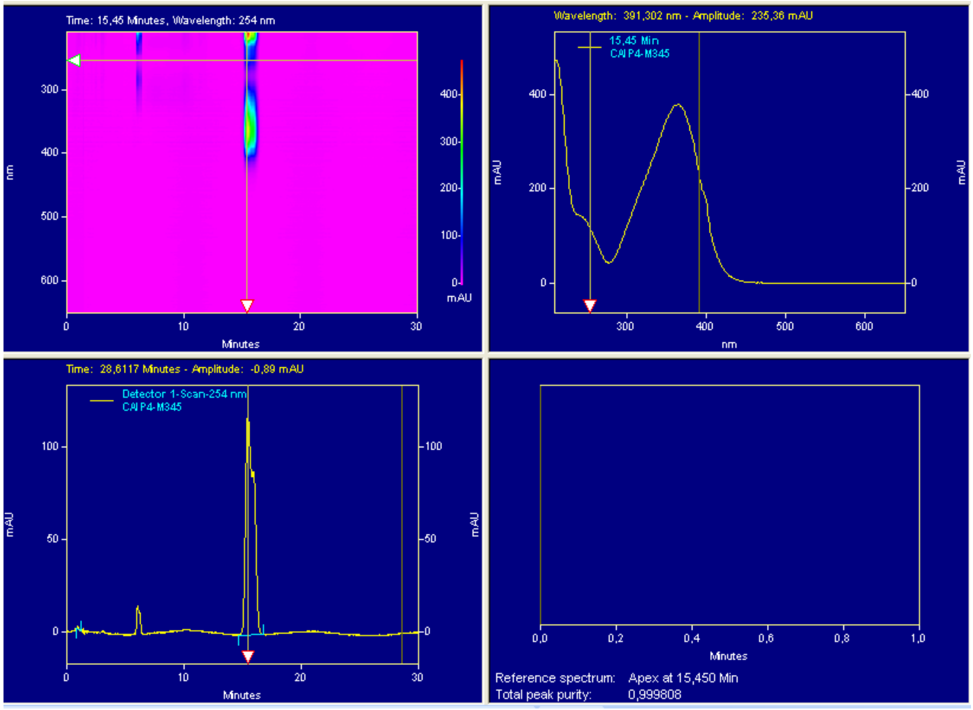

Retention Time	Area	Area %	Height	Height %
0.883	13211	0.23	1325	1.09
15.450	5785383	99.77	119752	98.91
Totals	5798594	100.00	121077	100.00

Figure S31. Peak purity of compound **6**.

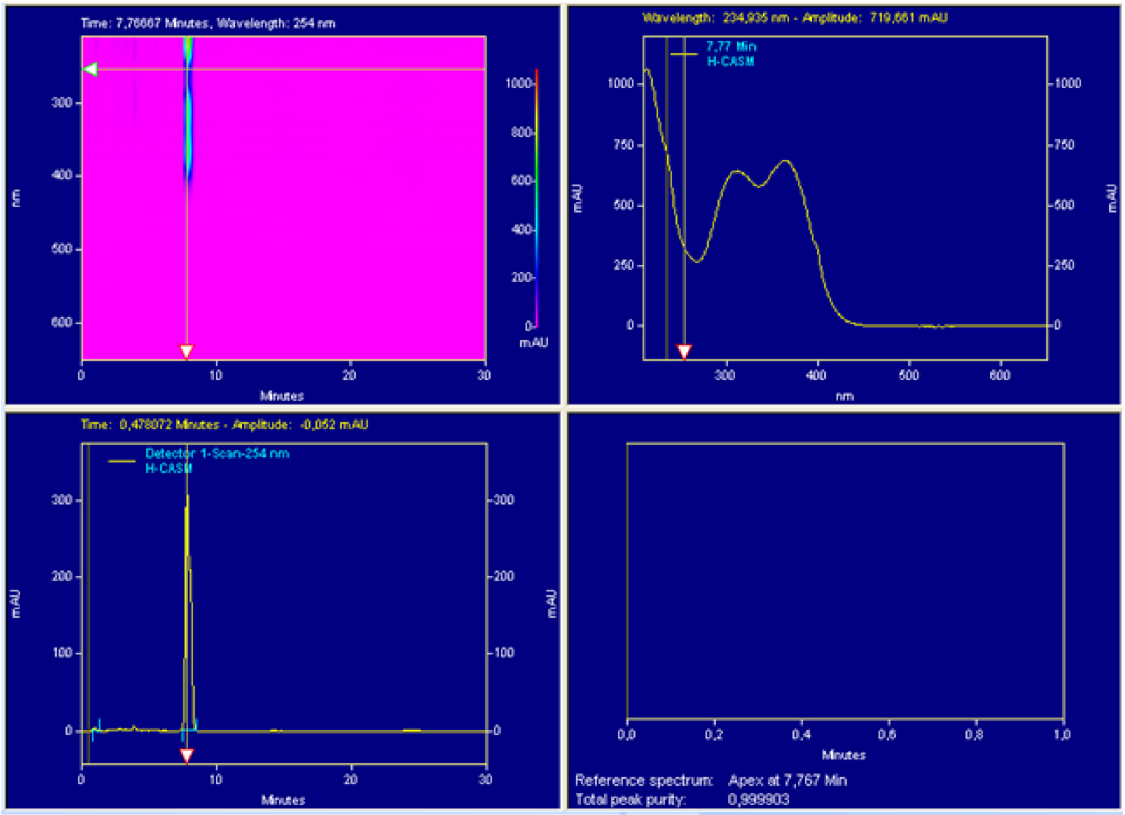

Retention Time	Area	Area %	Height	Height %
0.983	31507	0.38	3757	1.13
7.767	8166820	99.62	329769	98.87
Totals	8198327	100.00	333526	100.00

Figure S32. Peak purity of compound **7**.

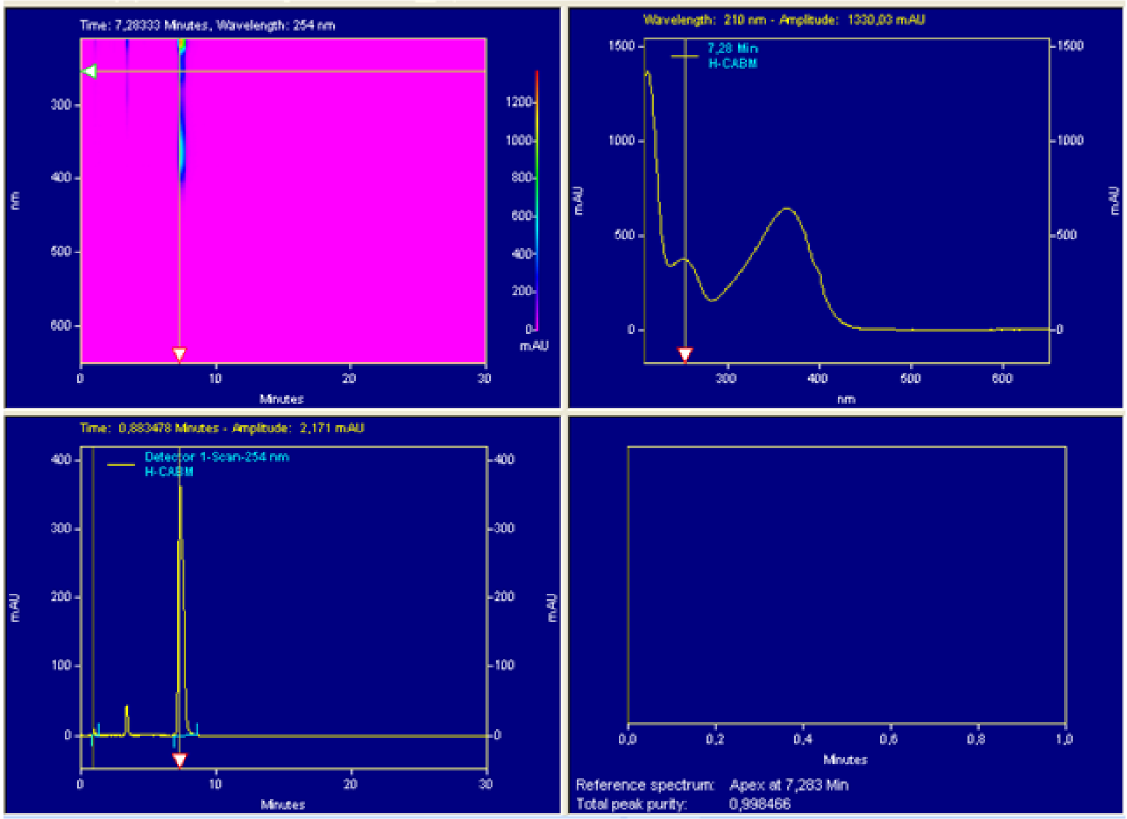

Retention Time	Area	Area %	Height	Height %
1.000	45940	0.49	8348	2.19
7.283	9297982	99.51	372387	97.81
Totals	9343922	100.00	380735	100.00

Figure S33. Peak purity of compound **8**.

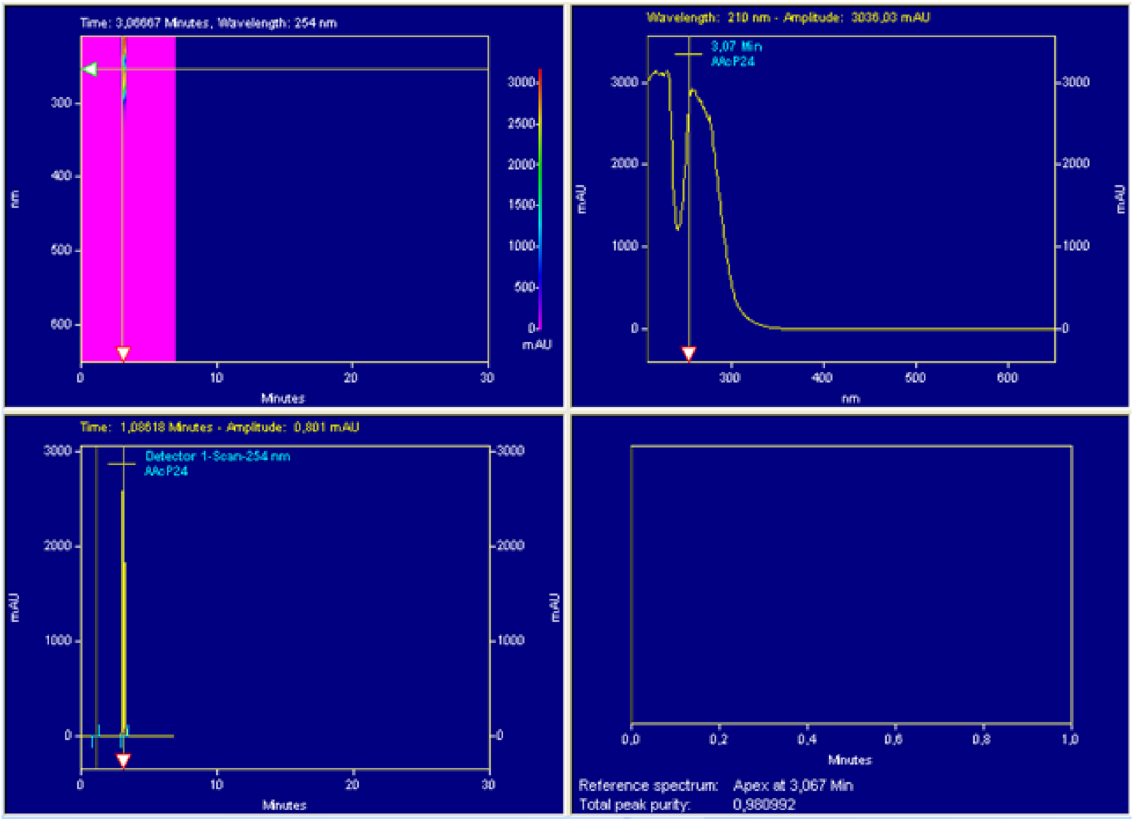

Retention Time	Area	Area %	Height	Height %
1.000	33939	0.27	2999	0.28
4.033	12632138	99.73	1069640	99.72
Totals	25022596	100.00	2718131	100.00

Figure S34. Peak purity of compound **9**.

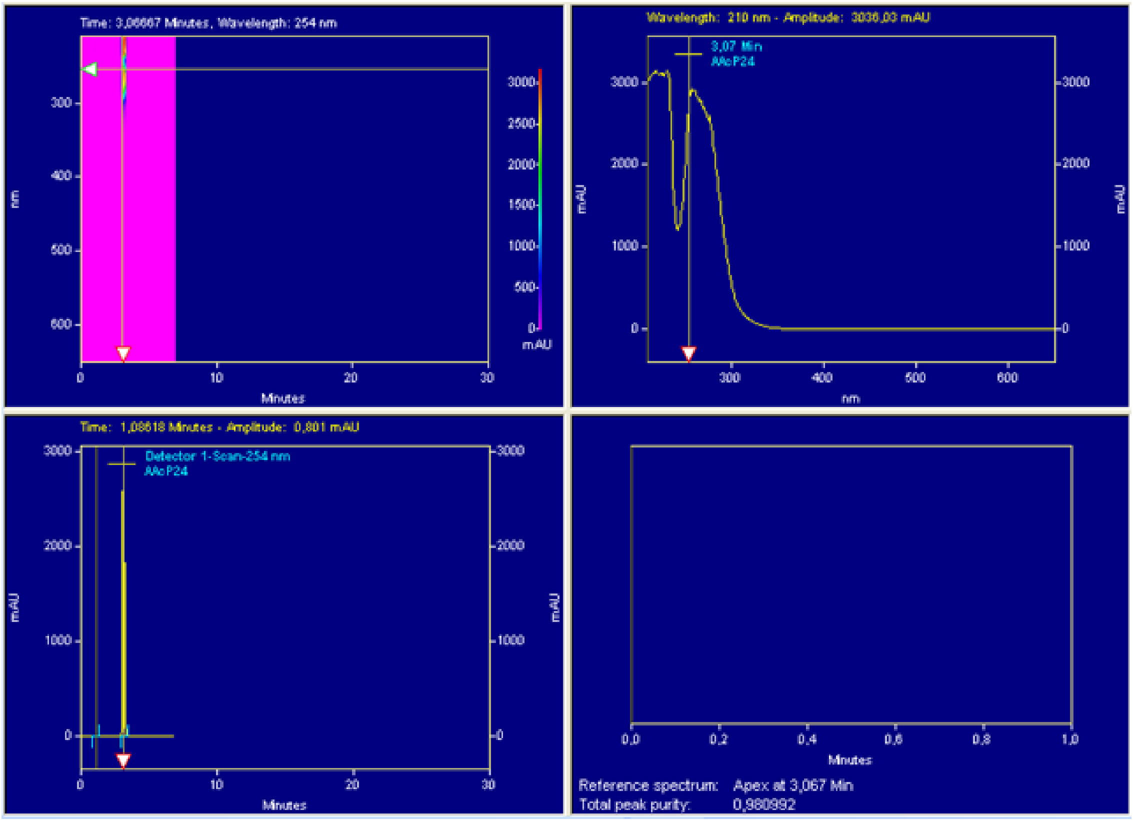

Retention Time	Area	Area %	Height	Height %
1.000	33939	0.27	2999	0.28
4.033	12632138	99.73	1069640	99.72
Totals	25022596	100.00	2718131	100.00

Figure S35. Peak purity of compound **10**.

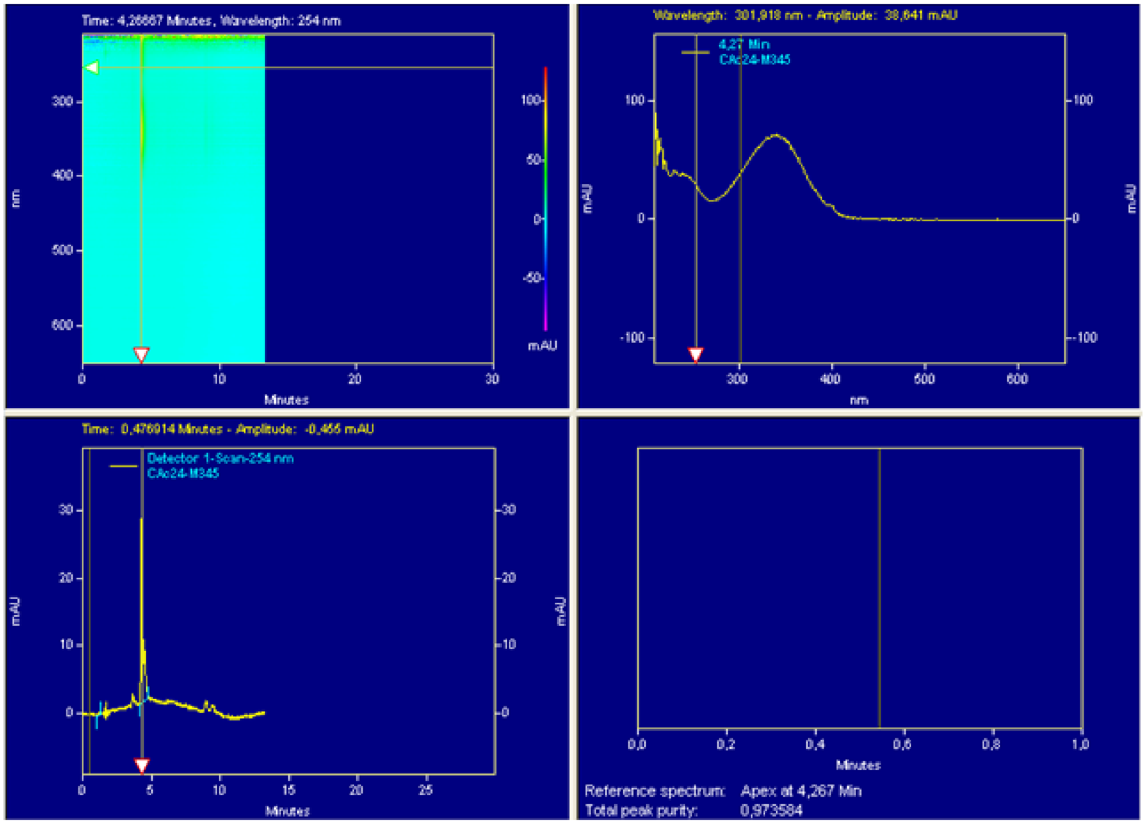

Retention Time	Area	Area %	Height	Height %
1.250	4403	1.38	358	1.28
4.267	314016	98.62	27620	98.72
Totals	318419	100.00	27978	100.00

Figure S36. Peak purity of compound **11**.

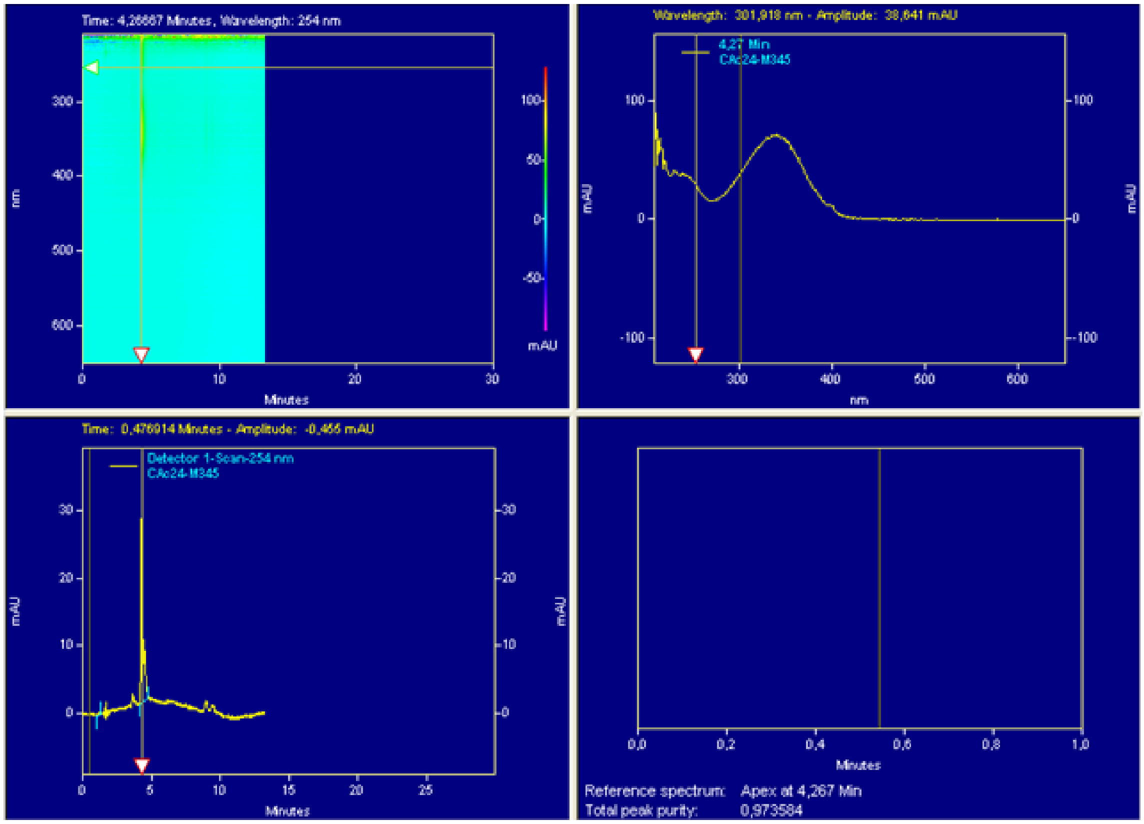

Retention Time	Area	Area %	Height	Height %
1.250	4403	1.38	358	1.28
4.267	314016	98.62	27620	98.72
Totals	318419	100.00	27978	100.00

Figure S37. Peak purity of compound **12**.

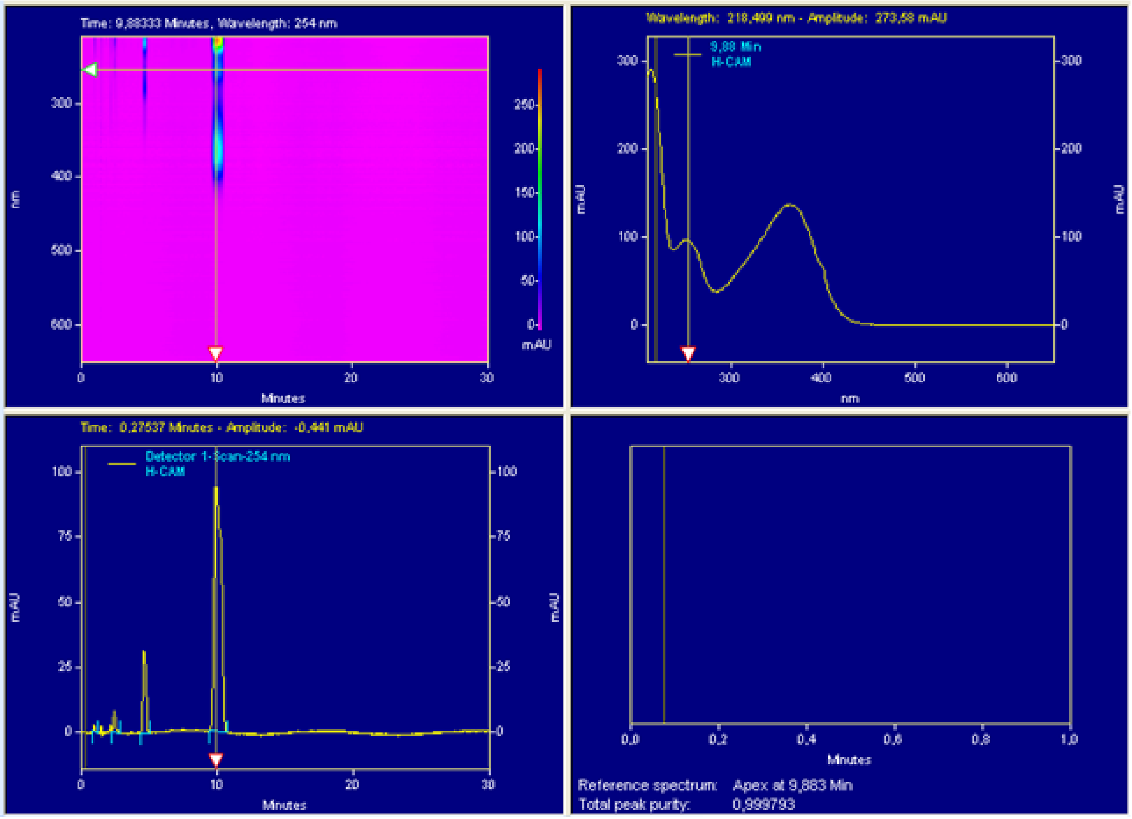

Retention Time	Area	Area %	Height	Height %
2.417	22154	0.53	2034	1.46
4.567	129499	3.12	7886	2.65
10.816	4007327	96.35	129506	95.89
Totals	4158980	100.00	139426	100.00

Figure S38. Peak purity of compound **13**.

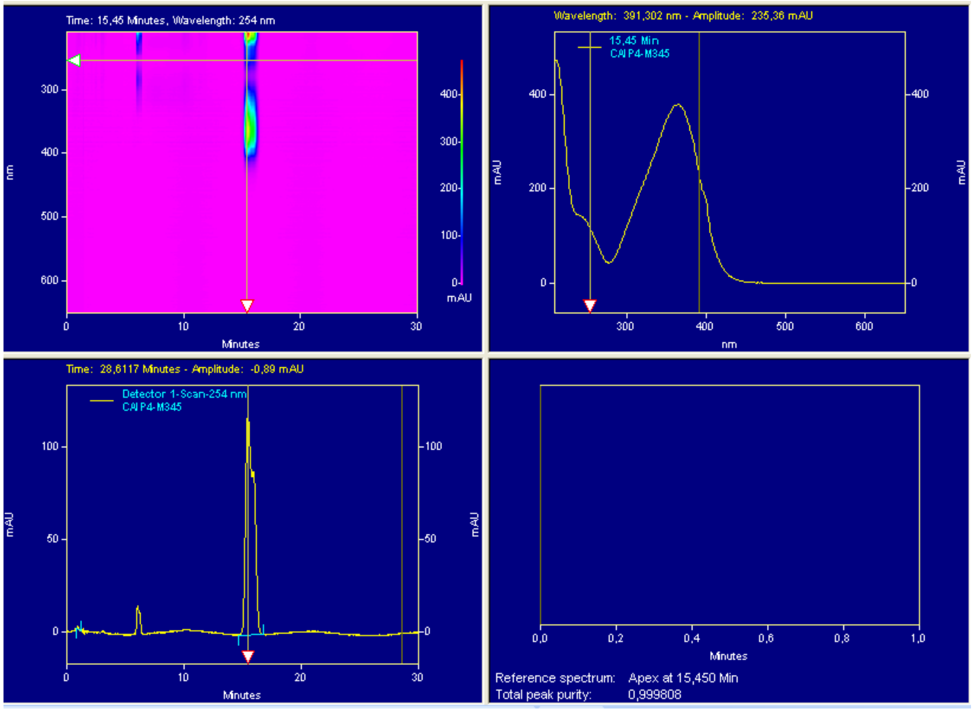

Retention Time	Area	Area %	Height	Height %
0.883	13211	0.23	1325	1.09
15.450	5785383	99.77	119752	98.91
Totals	5798594	100.00	121077	100.00

Figure S39. Peak purity of compound **14**.

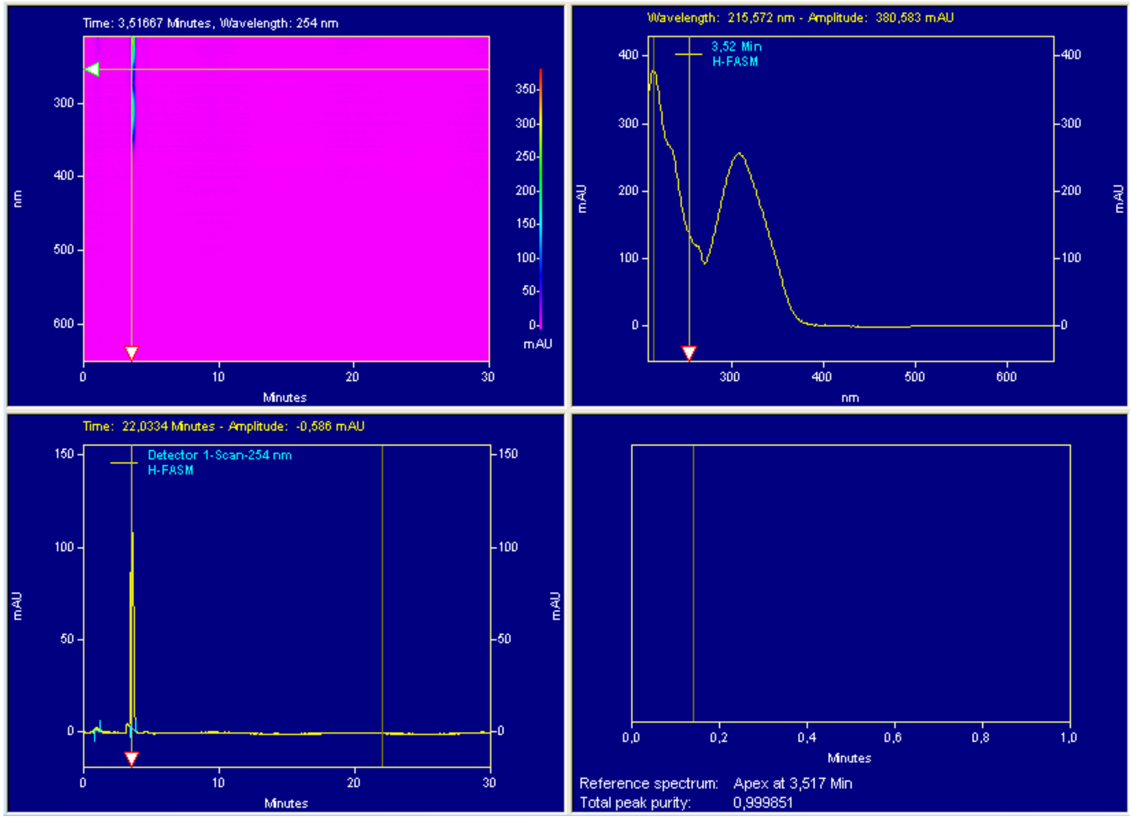

Retention Time	Area	Area %	Height	Height %
0.967	22330	1.39	2137	1.56
3.517	1583229	98.61	135235	98.44
Totals	1605559	100.00	137372	100.00

Figure S40. Peak purity of compound **15**.

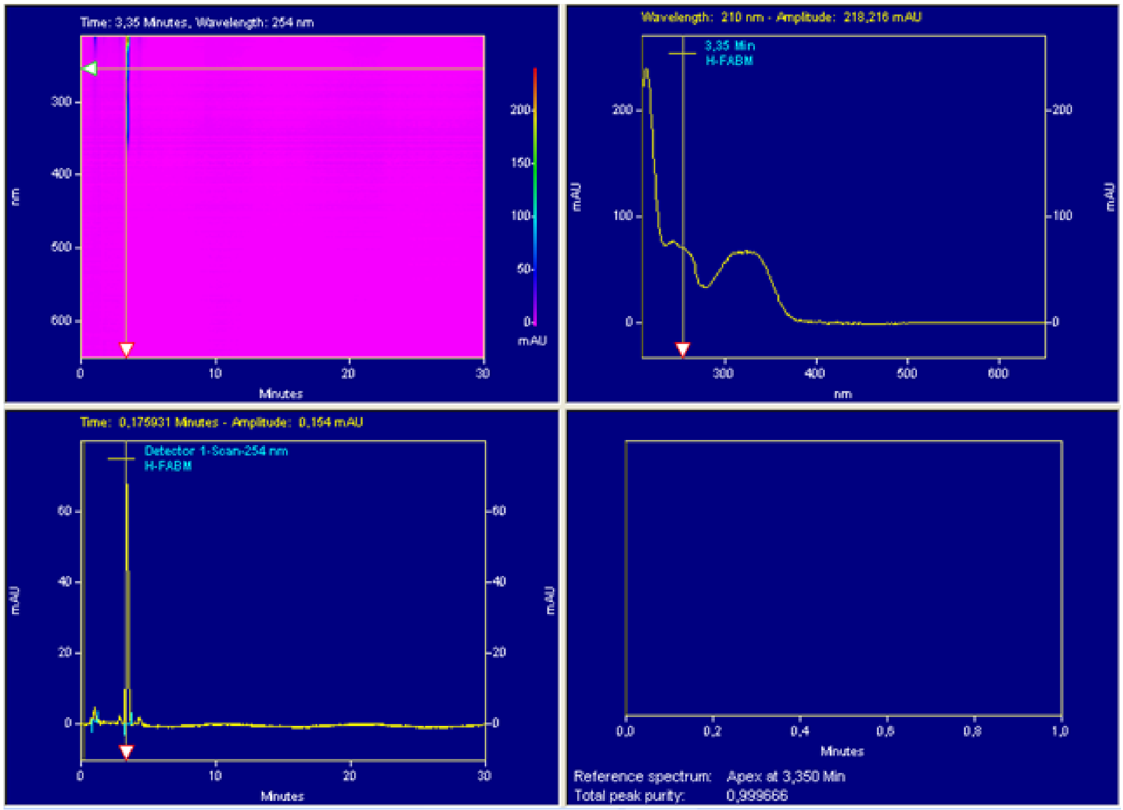

Retention Time	Area	Area %	Height	Height %
0.983	33784	4.10	3861	4.19
3.350	789853	95.90	70521	95.81
Totals	823637	100.00	74382	100.00

Figure S41. Peak purity of compound **16**.

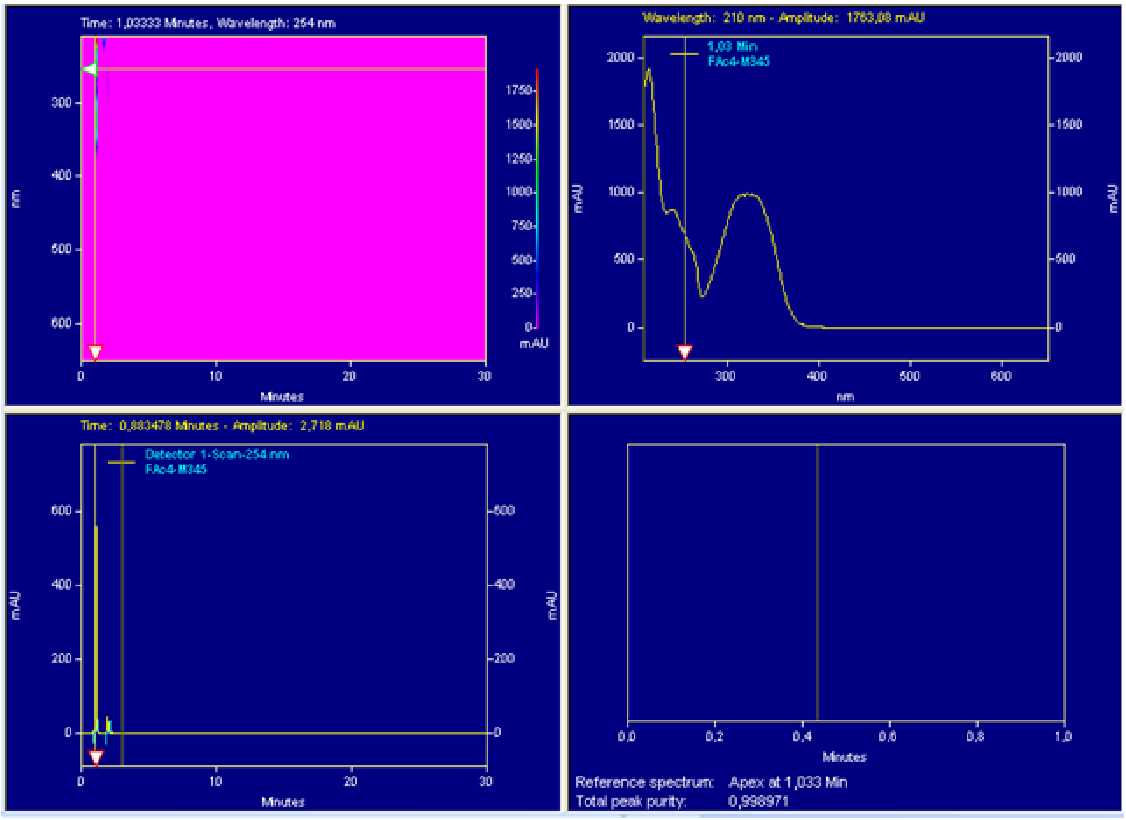

Retention Time	Area	Area %	Height	Height %
1.000	11156	0.45	1933	1.00
2.283	16269	0.66	2249	1.17
4.067	2297070	94.29	180967	96.63
5.450	51952	2.13	2747	1.42
Totals	2436810	100.00	193238	100.00

Figure S42. Peak purity of compound **17**.

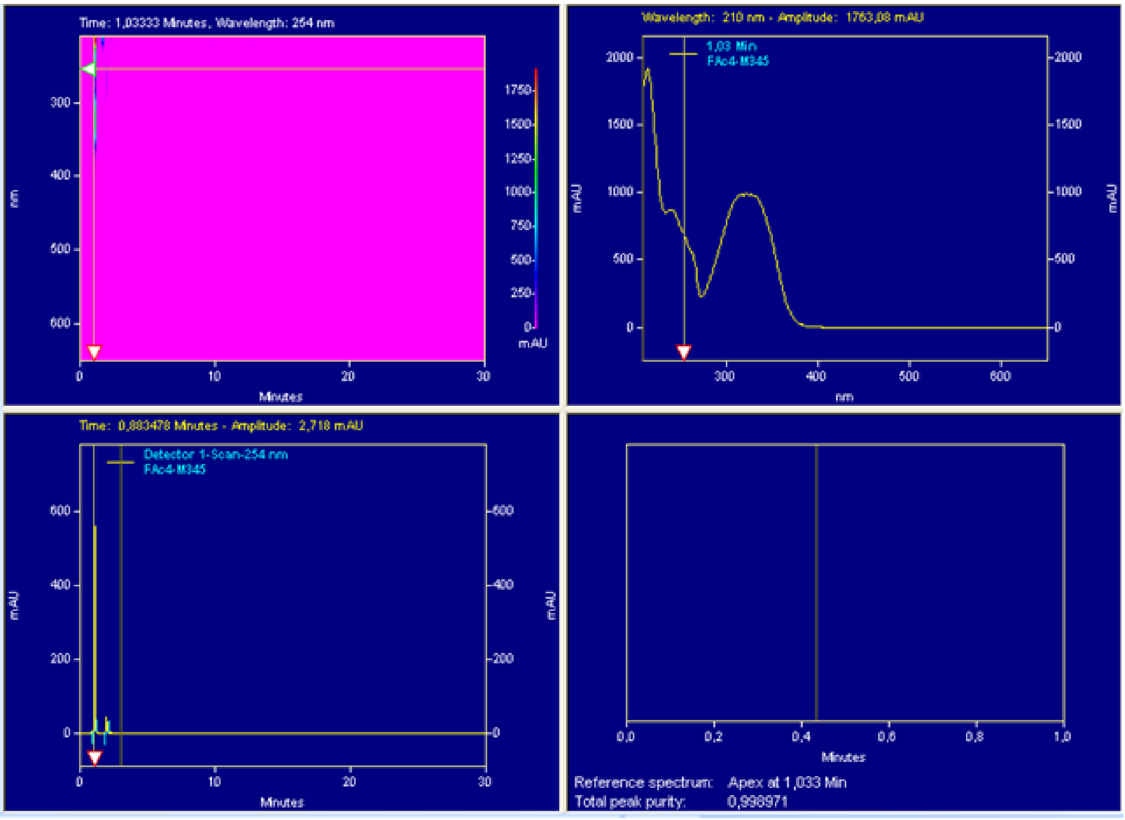

Retention Time	Area	Area %	Height	Height %
1.033	2838521	95.93	701659	95.93
1.900	120429	4.07	29769	4.07
Totals	2958950	100.00	731428	100.00

Figure S43. Peak purity of compound **18**.
